# Structural variant landscapes reveal convergent signatures of evolution in sheep and goats

**DOI:** 10.1186/s13059-024-03288-6

**Published:** 2024-06-06

**Authors:** Ji Yang, Dong-Feng Wang, Jia-Hui Huang, Qiang-Hui Zhu, Ling-Yun Luo, Ran Lu, Xing-Long Xie, Hosein Salehian-Dehkordi, Ali Esmailizadeh, George E. Liu, Meng-Hua Li

**Affiliations:** 1 State Key Laboratory of Animal Biotech Breeding, China Agricultural University, Beijing, 100193 China; 2 College of Animal Science and Technology, China Agricultural University, Beijing, 100193 China; 3 CAS Key Laboratory of Animal Ecology and Conservation Biology, Institute of Zoology, Chinese Academy of Sciences (CAS), Beijing, 100101 China; 4 College of Life Sciences, University of Chinese Academy of Sciences (UCAS), Beijing, 100049 China; 5 Department of Animal Science, Faculty of Agriculture, Shahid Bahonar University of Kerman, Kerman, 76169-133 Iran; 6 Animal Genomics and Improvement Laboratory, BARC, USDA-ARS, Beltsville, MD 20705 USA

**Keywords:** Structural variant, Genome assembly, Whole-genome sequence, Convergent evolution, Production traits, Domestication, Sheep, Goat

## Abstract

**Background:**

Sheep and goats have undergone domestication and improvement to produce similar phenotypes, which have been greatly impacted by structural variants (SVs). Here, we report a high-quality chromosome-level reference genome of Asiatic mouflon, and implement a comprehensive analysis of SVs in 897 genomes of worldwide wild and domestic populations of sheep and goats to reveal genetic signatures underlying convergent evolution.

**Results:**

We characterize the SV landscapes in terms of genetic diversity, chromosomal distribution and their links with genes, QTLs and transposable elements, and examine their impacts on regulatory elements. We identify several novel SVs and annotate corresponding genes (e.g., *BMPR1B*, *BMPR2*, *RALYL*, *COL21A1*, and *LRP1B*) associated with important production traits such as fertility, meat and milk production, and wool/hair fineness. We detect signatures of selection involving the parallel evolution of orthologous SV-associated genes during domestication, local environmental adaptation, and improvement. In particular, we find that fecundity traits experienced convergent selection targeting the gene *BMPR1B*, with the DEL00067921 deletion explaining ~10.4% of the phenotypic variation observed in goats.

**Conclusions:**

Our results provide new insights into the convergent evolution of SVs and serve as a rich resource for the future improvement of sheep, goats, and related livestock.

**Supplementary Information:**

The online version contains supplementary material available at 10.1186/s13059-024-03288-6.

## Background

Domestic animals or crops undergo similar phenotypic transformations while diverging from their wild progenitors, i.e., a phenomenon known as “domestication syndrome” [1–3]. For instance, livestock have been selected for a variety of analogous phenotypes, including behavioral (e.g., tameness), morphological (e.g., coat color), and production (e.g., high fertility) traits compared with their wild ancestors [2, 4]. However, the genetic changes that have driven convergent evolution across species during domestication and subsequent genetic improvement remain unclear.

Increasing evidence has suggested large functional impacts of SVs (including copy number variations—CNVs) on genome evolution, local adaptation, and phenotypic variations in livestock [5–8], but whole-genome characterizations of SVs and their functions have been rare. The genus *Ovis* and *Capra* first diverged at the late Miocene (e.g., 10.96 ± 0.73 Ma) during the adaptive radiation of caprines [9]. Domestic sheep (*Ovis aries*) and goats (*Capra hircus*) were domesticated in parallel from Asiatic mouflon and Bezoar ~10,000 years ago in the Middle East region and were subsequently spread throughout the world [10, 11]. Thus, sheep and goats represent an ideal system for studies on convergent evolution between closely related domestic species because of similar selective pressures on production traits (e.g., meat, milk, and wool/hair) during their domestication and genetic improvement. Genomic signatures of convergent evolution have been less frequently detected between sheep and goats [12, 13].

**Fig. 1:**
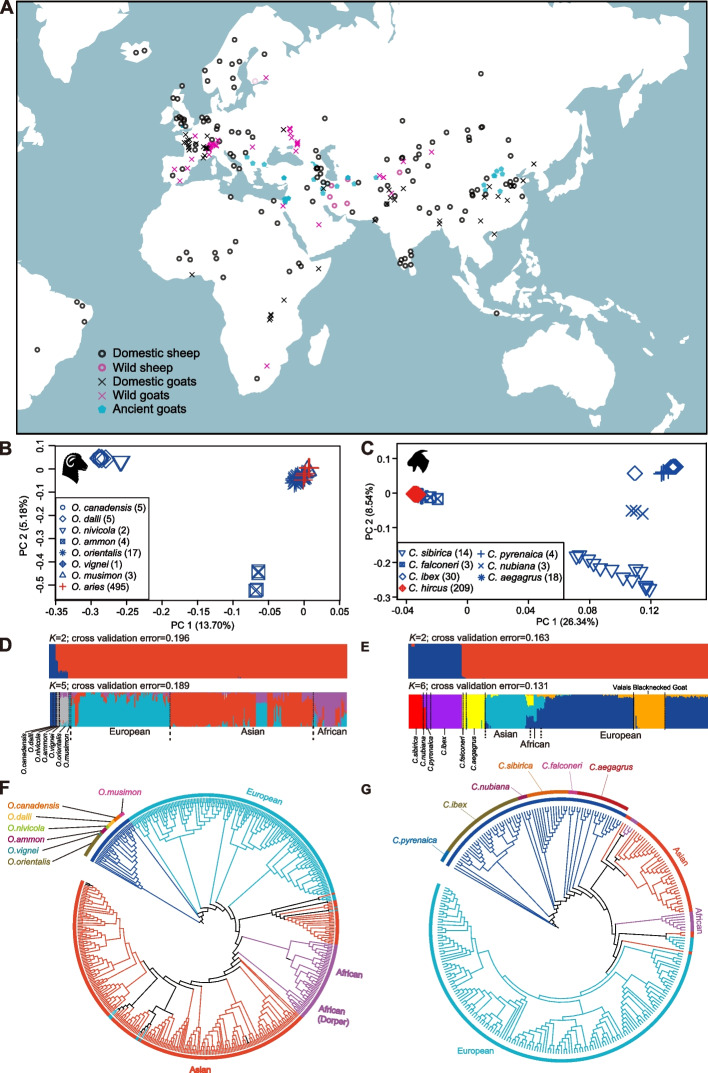
Geographic distribution and genetic structure of sheep and goat samples. **A** The geographic distribution of 532 modern sheep, 281 modern goats, and 84 ancient goats. **B**, **C** Principal component analysis (PCA) for sheep (**B**) and goats (**C**) based on SVs. **D**, **E** Population genetic structure analysis with assumed genetic cluster numbers of *K* = 2 and 5 for sheep (**D**) and *K* = 2 and 6 for goats (**E**) using SVs. **F**, **G** Phylogenetic tree of sheep (**F**) and goats (**G**) constructed by using the *p*-distances between individuals calculated from SVs

Here, we assembled a de novo high-quality reference genome of Asiatic mouflon (*Ovis orientalis*), the wild ancestor of sheep. We characterized the genome-wide landscape of SVs in a comprehensive dataset (sequencing depth > 15 ×) of worldwide wild and domestic populations of sheep and goats (Fig. 1A, Additional file 2: Table S1), which represent one of the largest datasets of SVs in mammals. Based on the dataset, we conduct a comprehensive study on the convergent evolution of mammals using SVs. We screened for the selection signatures of genomic SVs and relevant genes during domestication and the improvement of important agronomic traits such as those associated with reproduction. We also tested the genetic introgression of SVs from wild relatives to domestic populations and assessed the impacts of SVs on open chromatins (ATAC-seq peaks) and environmental adaptations. We aimed to reveal SV-genes (i.e., genes annotated with SVs) under convergent evolution in sheep and goats.

## Results

### De novo assembly and annotation of the Asiatic mouflon genome

We obtained the Asiatic mouflon chromosome-level genome assembly *Amuf_v1* (i.e., *CAU_Oori_1.0*, NCBI accession GCA_014523465.1), with a total size of 2.65 Gb, a contig N50 length of 42.16 Mb and a scaffold N50 length of 103.69 Mb, comprising 27 chromosomes of 44.04–282.18 Mb (Table 1). In the *Amuf_v1* genome, we predicted 20,042 genes based on gene structure, among which 18,790 (93.75%) were functionally annotated. Compared with the publicly available assemblies of wild sheep species at chromosome and scaffold level (Additional file 2: Table S2) [14–19], *Amuf_v1* has the longest scaffold N50 (103.69 Mb) and the second longest contig N50 (42.16 Mb) among the three chromosome-level genome assemblies of wild sheep species (argali, bighorn sheep, and Asiatic mouflon). Regarding the chromosome-level assemblies of domestic sheep, the assemble parameters (e.g., total length, contig N50, and scaffold N50) of *Amuf_v1* (Table 1) are well comparable to the latest reference genome of domestic sheep (*ARS-UI_Ramb_v3.0* in NCBI; total length of 2.65 Gb, contig N50 of 43.18 Mb, and scaffold N50 of 101.27 Mb) (Additional file 2: Table S2). These results indicated the good quality and high credibility of our Asiatic mouflon genome assembly.

**Table 1:** Assembly statistics of the Asiatic mouflon genome

**Genome assembly**	Asiatic mouflon
**Assembly statistics**	
Total length (Mb)	2652.57
Contig N50 (Mb)	42.16
Contig L50	23
Scaffold N50 (Mb)	103.69
Scaffold L50	8
**Annotation**	
Gene number	20,042
Gene density	7

Before starting the genome assembly process, we conducted an extensive genome survey, which revealed an estimated genome size of 2.49 Gb with a heterozygous ratio of 0.30%. This genome size is comparable to other *Ovis* species. Following the assemble of PacBio reads and subsequent polishing with Illumina reads, we obtained 343 contigs with a contig N50 length of 47.03 Mb. By using 321.12 Gb of BioNano clean data with an N50 of 317.60 kb, we successfully assembled the contigs into scaffolds. The mapping rate of the BioNano clean data reached 78.10%. We achieved a BioNano assembly with a total length of 2.81 Gb and an N50 of 95.97 Mb, and aligned the *Amuf_v1* contigs to the BioNano assembly. This resulted in a scaffold-level assembly of *Amuf_v1*, which had a size of 2.65 Gb and 193 scaffolds with a scaffold length of 107.10 Mb. In the final step, we utilized 298,676,845 valid Hi-C reads to anchor and orient the 193 scaffolds, culminating in the assembly of the *Amuf_v1* genome at the chromosome level which comprised a total of 27 chromosomes.

The mapping rate and genome coverage of the Illumina paired-end reads were estimated to be 99.89% and 99.73%, respectively. Benchmarking universal single-copy orthologue (BUSCO) analysis showed a high degree of completeness of the genome, and 98.57% of the complete eukaryotic universal genes covered most of the core conserved genic regions in the cetartiodactyla_odb10 database. The kmer analysis showed that the *Amuf_v1* had a kmer completeness of 93.15%. The BLAST analysis, which compared the 50-kb bins of the contigs against the NT database, indicated that 99.71% of the bins could be aligned with Metazoa. Together with that unbiased coverage and no contamination of the genome detected by the GC-depth analysis (GC content, 30–50%; reads depth, 50–70 × ; Additional file 1: Fig. S1A), these results again supported a high-quality genome assembly of *Amuf_v1*.

We conducted a detailed annotation of the *Amuf_v1* genome, revealing a total of 301,895 tandem repeats which comprise 0.40% of the genome. Furthermore, 4,920,791 transposable elements (TEs) were meticulously annotated, contributing 45.38% of the genomic content. By incorporating other unknown and simple repeats, our comprehensive annotation identified a total of 5,305,764 repeats in the *Amuf_v1* genome, which represent 46.07% of its entirety. Notably, Class I TEs emerged as the most abundant repeat type within the *Amuf_v1* genome, constituting 42.02% of the genomic composition. After masking the repeat content in the genome, we employed a combination of de novo, homology search, and transcript methods to annotate the gene structures. This resulted in the identification of 20,042 gene structures with an average length of 47.09 kb and an average coding sequence (CDS) length of 1.64 kb. Finally, we annotated the identified gene structures and obtained functional annotations for 18,790 genes, which constitute 93.75% of the entire gene sets.

### Genome features and synteny

We identified 18,777 gene families from the homologous protein sequences of Asiatic mouflon and six other species (i.e., sheep, goat, cattle, pig, horse, and mouse). Most (71–81%) of the gene families were single-copy genes (Additional file 1: Fig. S1B). Among the identified genes, 14,023 were shared by Asiatic mouflon, sheep, and goat (Additional file 1: Fig. S1C). The Pearson correlation analysis of GC content and gene density in the *Amuf_v1* genome revealed that higher gene density is moderately correlated to higher GC content (*r* = 0.37; Additional file 1: Fig. S1D, Additional file 2: Table S3). The distribution of presence and absence variation (PAV) indicated a similar count of insertions (23,896) and deletions (20,656) across the genome (Additional file 1: Fig. S1D). We observed a good collinearity (94.90%) between the reference genome of domestic sheep and Asiatic mouflon (Additional file 1: Fig. S1E), while a little lower collinearity (93.26%) occurred between *Amuf_v1* genome and the goat reference genome *ARS1* (NCBI accession GCA_001704415.1) (Additional file 1: Fig. S1E). In accordance with previous definition of synteny [20], our results revealed a remarkable synteny between the *Amuf_v1* genome assembly and the sheep reference genome *Oar_rambouillet_v2.0* (NCBI accession GCA_016772045.1), boasting a substantial ratio of 94.90%. This indicated that *Amuf_v1* was well assembled.

### SV discovery and characterization

The collected genomic data represented the most comprehensive samples, including 532 samples of 37 wild and 495 domestic sheep at a depth > 15 × and 281 samples of 72 wild and 209 domestic goats with most (255/281, 90.75%) depth > 15 × (Fig. 1A, Additional file 2: Table S1). Across wild and domestic sheep, the average sequencing depth was 18.32 × (15.02–30.11 ×), and the average genome coverage was 96.35% (95.50–97.02%) (Additional file 2: Table S4) [11]. The average depth was 21.45 × (5.97–41.06 ×), and the average coverage was 98.25% (94.43–98.92%) for wild and domestic goats (Additional file 2: Table S4). The genomic data of 84 ancient goat samples (*c*. 450–10,275 B.P.) [10, 21, 22] showed an average sequencing depth of 0.836 × (Fig. 1A, Additional file 2: Table S5).

**Fig. 2:**
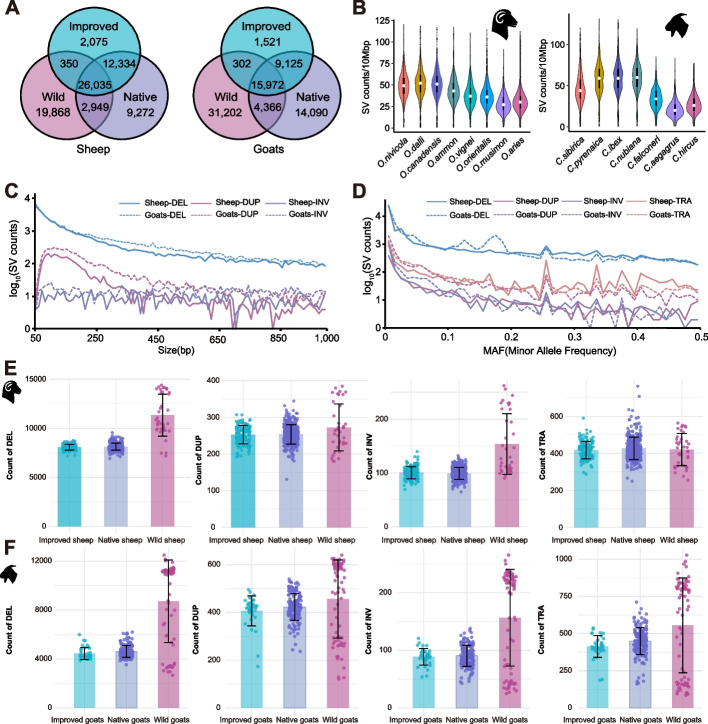
Characterization of structural variation call sets. **A** Venn diagrams of SV numbers among wild, native, and improved sheep and goats. **B** The distribution of SV numbers per 10 Mb among the species of the *Ovis* and *Capra* genera. **C** The size distribution (50–1000 bp) of SVs by variant types in sheep and goats. **D** Minor allele frequency distribution of SVs by variant types in sheep and goats. **E**, **F** The distribution of SV numbers in four SV types among wild, native, and improved groups of sheep (**E**) and goats (**F**). The colored dots represent different groups of sheep and goats. For detailed information about wild, native, improved sheep and goats, please see Additional file 2: Table S1

In the ovine and caprine samples, we identified a total of 72,883 (7452–15,608, an average of 9117 per individual) and 86,283 SVs (2918–14,375, an average of 6654 per individual) of 50 bp–1 Mb by at least two of the three analytical tools (see “Methods”), respectively (Fig. 2, Additional file 1: Fig. S2, Additional file 2: Table S6). We observed similar SV distribution patterns between the two taxa at the species and population levels (Additional file 1: Supplementary Results).

Within each taxon, a larger amount of SVs were detected in the wild (sheep, 49,202; goat, 51,842) and native (sheep, 50,590; goat, 43,553) populations than in the improved populations (sheep, 40,794; goat, 26,920). Wild populations harbored much more unique SVs (sheep, 19,868; goat, 31,202) than those in the native/landraces (sheep, 9272; goat, 14,090) and improved populations (sheep, 2075; goat, 1521) (Fig. 2A). We observed more SVs shared between native and improved populations in both sheep (38,369) and goats (25,097) (Fig. 2A).

Regarding the various types of SVs, deletions were the most prevalent type in both sheep and goats (Fig. 2C–F, Table 2). The distribution of SV frequencies was skewed towards rare alleles, with 26,157 SVs (35.89%) in sheep and 28,160 SVs (32.51%) in goats exhibiting a minor allele frequency (MAF) < 0.01 (Fig. 2D). Additionally, MAF spectra of different SV types in sheep and goats differed in their distributions (Fig. 2D), similar to what was reported in humans previously [23]. Regarding the distribution of SV length, most SVs were shorter than 1 kb (Fig. 2C, Additional file 1: Supplementary Results and Fig. S3, Additional file 2: Table S7).

**Table 2:** Summary information of sheep and goat genomes used in this study

**Organism**	**Sample**	**Deletion**	**Duplication**	**Inversion**	**Insertion**	**Translocation**	**Total**
**Sheep**							
Domestic sheep	495	40,850	2737	1011	1	8416	53,015
European mouflon	3	9412	404	163	0	805	10,784
Asiatic mouflon	17	30,418	1156	585	0	2501	34,660
Urial	1	10,483	235	99	0	335	11,152
Argali	4	15,290	430	260	0	902	16,882
Snow sheep	2	14,932	378	2237	0	602	16,149
Thin horn sheep	5	17,061	558	348	0	1009	18,976
Big horn sheep	5	16,798	538	323	0	978	18,637
**Goats**							
Domestic goats	209	33,743	3909	1237	0	6851	45,740
Bezoar	18	16,236	1438	370	0	1484	19,528
Markhor	3	8799	782	269	0	1188	11,038
Siberian ibex	14	16,547	1225	406	0	1911	20,089
Alpine ibex	30	13,858	1222	378	0	2810	18,268
Nubian ibex	3	14,026	882	324	0	1564	16,796
Iberian ibex	4	13,033	883	322	0	1550	15,788

### Novel SVs and experimental validation

**Fig. 3:**
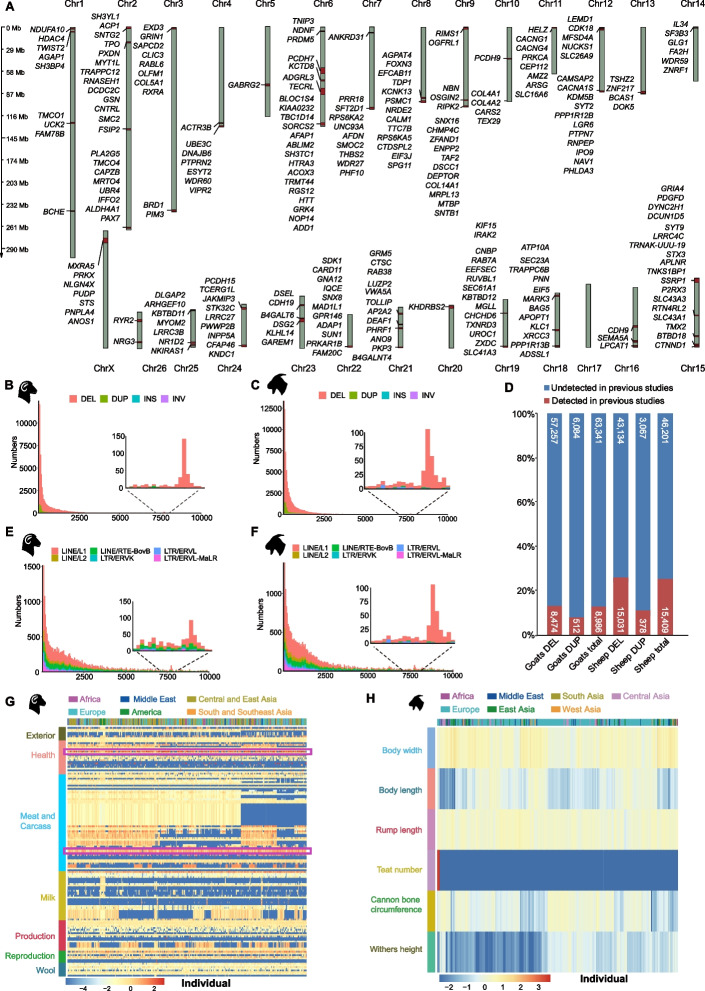
Genomic landscape of SVs in sheep and goats. **A** Chromosomal distribution of SV hotspot regions in the sheep genome and 250 commonly annotated SV-genes of sheep and goats in hotspot regions. **B**, **C** The distribution of whole SVs with lengths of 1–10,000 bp in the domestic sheep (**B**) and goat (**C**) genomes. **D** Comparisons of the identified SVs with those reported in previous studies. **E**, **F** The distribution of TEs with a length of 1–10,000 bp in the domestic sheep (**E**) and goat (**F**) genomes. **G**, **H** Enrichment of SVs within different QTLs of sheep (**G**) and goats (**H**). The log_2_(fold enrichment) scores of SVs in each QTL for each animal are visualized in a heatmap. The most highly enriched QTLs of sheep are indicated with purple rectangular boxes

We compared the SVs identified herein with published SV catalogs (Additional file 2: Table S8). After the conversion of genome coordinates, we found that substantial numbers of deletions (DELs) (ovine: 43,134, 74.16%; caprine: 57,257, 87.11%) and duplications (DUPs) (ovine: 3,067, 89.03%; caprine: 6,084, 92.24%) had gone undetected in previous studies (Fig. 3D, Additional file 2: Table S9). In total, 74.99% (46,201) of the ovine DEL and DUP variants and 87.58% (63,341) of the caprine DEL and DUP variants identified here were novel SVs (Fig. 3D, Additional file 2: Table S9).

Furthermore, 17 DELs and 6 DUPs were randomly selected and experimentally inspected in 12–15 sheep samples via PCR or qPCR. The experimental results showed a validation rate of 76.69% concordant genotypes (212/249 deletions and 38/77 duplications; Additional file 1: Fig. S4, Additional file 2: Table S10), comparable to the rates (78.05–85.4%) reported in previous studies [24–26].

### Distribution of SV hotspots

Based on the genomic positions of SV breakpoints, we identified 260 and 191 SV hotspots (i.e., the regions with the top 10% of SV breakpoints) on chromosomes in sheep and goats, which covered a total length of 392 and 346 Mb, respectively (Fig. 3A, Additional file 1: Fig. S5). The SVs in the hotspot regions were annotated to 1547 and 1591 genes in sheep and goats, respectively, among which 250 genes were shared between the two ruminants and distributed across all chromosomes of the genomes (Fig. 3A). By comparing the hotspots with known QTLs, we identified 120 hotspots overlapping with 401 QTLs for production traits such as milk yield and loin yield in sheep and 7 hotspots overlapping with 7 QTLs for body length, udder depth, teat number, bone quality, and teat placement in goats (Additional file 2: Table S11).

Furthermore, we calculated the numbers of SV breakpoints located in the telomeric regions and nontelomeric regions. We observed 16,111 (11.07%) and 25,849 (15.83%) of the SV breakpoints in the telomeres of sheep (269 Mb) and goats (290 Mb), respectively (Additional file 2: Table S12). Statistical tests indicated that SV breakpoints were significantly enriched in telomeres in both sheep (Wilcoxon rank-sum = 472,165, *P* = 1.21 × 10^−8^) and goats (Wilcoxon rank-sum = 2,544,203, *P* = 5.95 × 10^−41^).

### SV-associated genes and transposable element-associated SVs

We annotated the SVs and found that the majority of SVs (sheep, 57.38%, 43,216; goats, 55.97%, 49,629) were located in the intergenic regions, followed by intronic (sheep, 32.14%, 24,207; goats, 34.27%, 30,384) and exons (sheep, 2.55%, 1,919)/upstream (goats, 2.15%, 1908) (Table 3, Additional file 2: Tables S13 and S14).

**Table 3:** SV features in the whole and common SV-annotated genes of sheep and goats (sheep/goats)

	**Annotation**	**Deletion**	**Duplication**	**Inversion**	**Translocation**	**Total**
**SVs annotated on the whole genes**	Intronic	20,231/23,960	1062/2437	448/829	2466/3158	24,207/30,384
	Exonic	1702/1308	70/186	58/65	89/83	1919/1642
	Upstream	1079/1404	44/136	15/57	308/311	1446/1908
	Downstream	1106/1323	71/128	29/68	252/289	1458/1808
	3′ UTR	322/277	17/48	63/43	455/314	857/682
	5′ UTR	211/194	14/23	2/7	209/151	436/375
	**Total**	24,651/28,466	1278/2958	615/1069	3779/4306	30,323/36,799
**SVs annotated on common genes**	Intronic	15,995/18,587	846/1893	364/624	2126/2417	19,331/23,521
	Exonic	873/685	49/82	32/33	61/38	1015/838
	Upstream	492/524	24/54	11/15	166/140	693/733
	Downstream	505/534	32/49	11/24	135/112	683/719
	3′ UTR	199/150	9/28	47/31	349/207	604/416
	5′ UTR	105/99	8/12	1/6	130/83	244/200
	**Total**	18,169/20,579	968/2118	466/733	2967/2997	22,570/26,427

We identified 10,310 and 11,746 functional genes containing at least one SV among the sheep and goat genomes, respectively (Additional file 2: Tables S13 and S14). Among these SV-associated genes, the majority of genes (5,904) were shared between sheep and goats (Additional file 1: Fig. S5, Additional file 2: Table S15). Of the 10,310 SV-gene identified for sheep, 1420 (13.77%) and 7768 (75.34%) genes had SVs in the exon and intron, respectively. The proportion of genes having SVs in the intron and exon is 5.47. Among the 11,746 SV-gene identified for goats, 1439 (12.25%) and 8235 (70.11%) genes had SVs in the exon and intron, respectively. The proportion of genes having SVs in the intron and exon is 5.72.

To further reveal the functional implications of the SV-genes (i.e., the genes overlapped with SVs), we compared SV locations with QTL regions in sheep and goats. We identified a total of 4564 SVs in sheep and 342 SVs in goats that overlapped with 342 and 7 QTLs, respectively (Additional file 2: Table S16). These QTLs were largely associated with body weight-, carcass-, and fiber-related traits in sheep and body size-related traits in goats. Furthermore, the enrichment analysis of SVs among different QTLs revealed that the SVs were mostly enriched in one disease-related QTL (scrapie susceptibility) and two meat-related QTLs (longissimus muscle area and longissimus muscle depth) in sheep and two QTLs for morphological traits (body width and rump length) in goats (Fig. 3G, H, Additional file 2: Tables S17 and S18), implying potentially important roles of SV-genes in production traits.

In the ovine and caprine genomes, the most abundant transposable element (TE) families associated with the identified structural variants were L1, L2, and RTE-BovB of LINEs (long interspersed nuclear element), tRNA-Core-RTE, Core-RTE, and MIR of SINEs (short interspersed nuclear element), ERVL-MaLR, ERVL and ERVK of LTRs (long terminal repeat), and hAT-Charlie of DNA transposons (Fig. 3B, C, E, F, Additional file 1: Fig. S6, Additional file 2: Table S19). In both the *Ovis* and *Capra* genera, the distribution of the SV and TE families showed increased numbers of SVs with lengths of 100–150 bp and 7500–8000 bp, which could probably come from the tRNA-Core-RTE and L1/RTE-BovB families, respectively (Fig. 3B, C, E, F, Additional file 1: Fig. S6).

### Genetic diversity and population structure

**Fig. 4:**
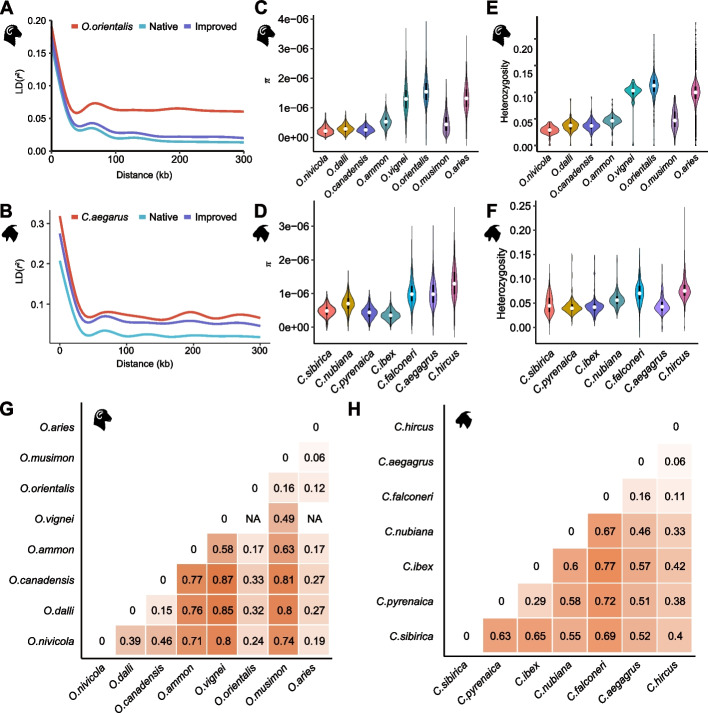
Genetic diversity of sheep and goat samples based on structural variations. **A** The pattern of linkage disequilibrium (LD) decay in the genomes of Asiatic mouflon, native sheep, and improved sheep. **B** The pattern of linkage disequilibrium (LD) decay in the genomes of bezoar, native goat, and improved goat. **C** Genome-wide nucleotide diversity (*π*) of the eight sheep species in the genus *Ovis*. **D** Genome-wide nucleotide diversity (*π*) of the seven goat species in the genus *Capra*. **E** The heterozygosity ratio of SV sites in the eight sheep species in the genus *Ovis*. **F** The heterozygosity ratio of SV sites in the seven goat species in the genus *Capra*. **G** Pairwise *F*_ST_ values between the eight sheep species in the genus *Ovis*. **H** Pairwise *F*_ST_ values between the seven goat species in the genus *Capra*

The estimation of linkage disequilibrium (LD, measured as *r*^2^) between SVs showed similar patterns of LD decay between the ovine and caprine species. The lowest decay rate and the highest LD level were observed in the wild species, followed by those in the improved and native populations (Fig. 4A, B). The LD estimates between SNPs exhibited similar LD pattern in the wild, native, and improved sheep/goats (Additional file 1: Fig. S7). The nucleotide diversity (*π*) measured based on SVs of both domestic sheep (1.32e−06) and goats (1.32e−06) was close to that of their wild ancestors Asiatic mouflon (1.55e−06) and bezoar (1.00e−06), respectively (Fig. 4C, D). The heterozygosity value of domestic sheep (0.098) was lower than that of Asiatic mouflon (0.111), but domestic goats exhibited a higher value (0.078) than bezoar (0.045) (Fig. 4E, F). Pairwise genome-wide *F*_ST_ values calculated based on SVs were 0.06–0.87 between wild and domestic sheep and 0.06–0.77 between wild and domestic goats (Fig. 4G, H). Lower estimated *F*_ST_ values were observed between the domestic species and their wild ancestors (sheep versus Asiatic mouflon, 0.12; domestic goats versus bezoar, 0.06), implying close phylogenetic relationships [11, 12].

After controlling for the missing genotypes, 47,092 SVs in sheep and 58,279 SVs in goats were retained for population genetic structure analysis. PCA of 532 modern sheep showed that domestic sheep were more closely related to Asiatic mouflon and its close relative European mouflon [11] than the other wild sheep species (Fig. 1B). Similarly, PCA of 281 modern goats revealed close relationships between domestic goats and bezoar and their close relative markhor (Fig. 1C) [27]. Additional PCAs of only domestic sheep or domestic goats separated the European, Asian, and African populations into distinct groups (Additional file 1: Fig. S8). Model-based structure analysis showed separate clusters of wild species and Asian, African, and European populations of domestic sheep (*K* = 5) and goats (*K* = 6) (Fig. 1D, E, Additional file 1: Fig. S9), largely congruent with the population divergence inferred by PCA. In line with the PCA and structure analyses, the phylogenetic tree showed that domestic sheep or goat populations could be divided into three major groups of different continental origins (i.e., Europe, Asia, and Africa), while the clades of wild species were located beyond domestic populations (Fig. 1F, G). Additionally, African sheep were divided into two different lineages (i.e., Dorper and the other African sheep populations) in the phylogenetic tree (Fig. 1F), as reported previously based on whole-genome SNPs [24].

### Selection signatures of SVs during domestication and improvement

To identify the signatures of convergent selection on SVs and associated genes during domestication, we compared the genomes of indigenous domestic populations (native sheep and goats in the Middle Eastern domestication center) with their wild ancestors (Asiatic mouflon and bezoar). Based on the SVs with the *P* values of *F*_ST_ < 0.05 and the top 5% of DI_SV_ values, we detected 445 and 409 candidate genes associated with sheep and goat domestication, respectively (Additional file 1: Fig. S10, Additional file 2: Table S20). Functional annotation of the 445 domestication-associated genes in sheep revealed significantly (FDR < 0.1) enriched GO terms and pathways associated with mucin type O-glycan biosynthesis, long-term depression, and inflammatory mediator regulation of TRP channels (Additional file 2: Table S21). In goats, the 409 domestication-related genes were significantly (FDR < 0.1) enriched in similar GO terms and pathways associated with neural system and signalling processes, such as neurotransmitter secretion, signal release from synapse, and cell-cell signalling (Additional file 2: Table S21). Notably, we detected 31 common domestication-related genes between sheep and goats (Additional file 2: Table S22), implying convergent selection signatures during their domestication. These common genes (e.g., *GRID2*, *PRKG1*, *BMPR2* and *TMEM117*) had important functions in temperament regulation, environmental adaptation, reproduction, and composition traits (Additional file 2: Table S23) [28–31].

Taking advantage of publicly available ancient goat genomes, we explored whether common genes were selected during the different stages of domestication and early development (stage I—from bezoar to ancient domestic goat, and stage II—from ancient domestic goat to modern native goat populations). We genotyped a total of 84 ancient goat samples and detected one or more alleles in the samples. We combined the SVs from ancient and modern goats and performed pairwise *F*_ST_ estimates between populations of bezoar and ancient goats to identify SVs and genes associated with domestication stage I (Additional file 1: Fig. S11A). We annotated 72 genes associated with the SVs with the top 5% of *F*_ST_ values, among which 8 genes (*C7H5ORF63*, *LOC102174140*, *DNER*, *KANK1*, *FRMPD1*, *MDGA2*, *LRRC36*, and *SCFD2*) overlapped with the domestication-related genes identified above based on whole-genome SVs (Additional file 2: Tables S20 and S24). To reveal the SVs and genes involved in subsequent development (stage II), we conducted pairwise *F*_ST_ tests between ancient and modern native goat genomes (Additional file 1: Fig. S11B). We identified 160 genes based on the top 5% of *F*_ST_ values, including 16 genes (*PRKCI*, *DNER*, *LOC108637685*, *CACNA2D1*, *DGKB*, *TRNAC-GCA*, *KCTD8*, *KANK1*, *SCFD2*, *MDGA2*, *MYO16*, *GPC5*, *TRNAS-GGA*, *LRRC36*, *PRKCB*, and *LOC102181832*) shared with the domestication-related genes (Additional file 2: Tables S20 and S24). Altogether, we identified 5 common SV-genes (*DNER*, *KANK1*, *MDGA2*, *LRRC36*, and *SCFD2*) under selection during the two stages (stages I and II) of domestication and early development. These genes showed important functions related to neurodevelopment and neurological function (*DNER*, *KANK1*, and *MDGA2*) [32–34], implying that the transformation of the neutral system may have consistently been selected during the domestication and subsequent development of sheep and goats.

To identify the SVs and relevant genes that were potentially selected during recent genetic improvement, we estimated the global *F*_ST_ values across the genomes of domestic populations. Based on the top 5% of *F*_ST_ estimates, we annotated 348 and 118 candidate genes associated with the recent improvement of sheep and goats, respectively (Additional file 1: Fig. S12, Additional file 2: Table S25). Functional annotation of the 348 candidate genes in sheep revealed significantly (FDR < 0.1) enriched GO terms involved in lipid metabolisms, such as lipid metabolic process (e.g., *ACER2*, *DGKI*, *PIP4K2A*) (Additional file 2: Table S21). In goats, the 118 candidate genes were significantly (FDR < 0.05) enriched in GO terms associated with transmembrane transporter activity (e.g., *ABCC1*, *SLC30A7*) (Additional file 2: Table S21). Additionally, we detected 6 candidate genes that were selected in both sheep and goats (e.g., *FAF1*, *MSRB3*, *SORCS2*, *TRAPPC12*, *TRNAW-CCA*, *USH2A*; Additional file 2: Table S22), which were related to disease resistance, climatic adaptation, and production traits [35–38].

### Candidate SV-genes selected for important agronomic traits

**Fig. 5:**
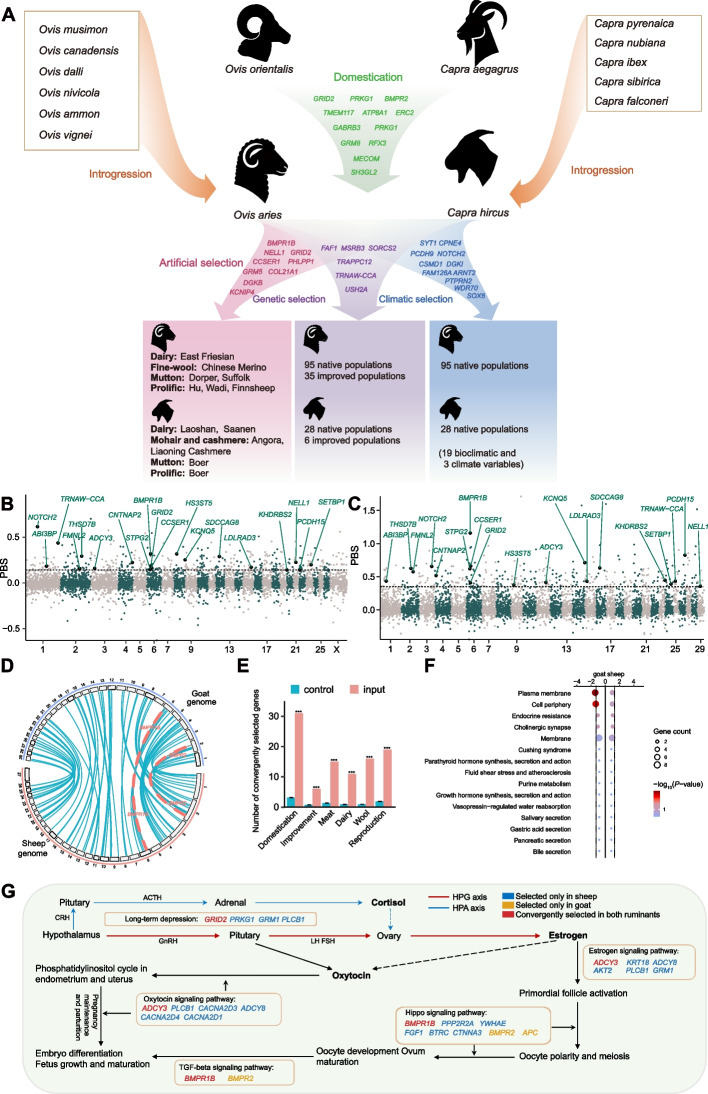
Overview of convergent evolution in sheep and goats at the scale of structural variants. **A** Convergent evolution through molecular parallelism of SV-genes involved in the domestication, genetic selection, climatic selection, and artificial selection of sheep and goats. The genes presented in the modules “Domestication”, “Genetic selection”, “Climatic selection”, and “Artificial selection” are the common candidate genes (i.e., orthologous genes) with important functions identified in the domestication, improvement, environment, and agronomic trait associated analyses of sheep and goats, respectively. **B** Genome-wide PBS values for reproduction traits between prolific sheep (WDS, HUS, SXW, FIN, GOT) and non-prolific sheep (BSB, SSS) populations. **C** Genome-wide PBS values for reproduction traits between prolific goat (BAR, BEE, BOE, DDP, KAM, NAC, TED) and non-prolific goat (CAG, PEC) populations. **D** The genomic distribution of the genes under convergent selection in sheep (red) and goat (blue) genomes. The peacock blue lines show convergently selected genes that are syntenic (e.g., under molecular parallelism) in the sheep and goat genomes. The red lines highlight the positions of the considered genes (*BMPR2* and *BMPR1B*) under continuous convergent selection for reproduction traits during domestication (*BMPR2*) and breeding (*BMPR1B*) processes. **E** Convergent selection acts on the identified genes more often than expected by chance in the different datasets of sheep and goats (*P* < 0.001, pairwise comparison via permutation test). **F** Enriched pathways and GO terms in sheep or goats identified using g:Profiler among the genes for reproduction traits under convergent selection. Circle size indicates the number of genes from the common gene hit list included in each enrichment item, and circle color and *x*-axis position indicate the *P* value. The vertical dashed lines indicate the significance threshold of FDR < 0.05. **G** Detailed molecular representation of convergent pathways and genes implicated in the female reproduction process. Detailed information for these genes is listed in Additional file 2: Table S22. In panels **B** and **C**, the horizontal dotted line represents the threshold of the top 5% of PBS values for each selective test. The genes convergently selected in sheep and goats are shown in the panels, and those reported previously to be associated with reproduction traits are represented in green font. For detailed information of the populations involved in the selective tests, please see Additional file 2: Table S1

To reveal the SVs and associated genes involved in important agronomic traits, we calculated PBS estimates between domestic populations with differential phenotypes (Additional file 2: Table S26). For the prolificacy trait, the SVs with the top 5% of PBS values between prolific and non-prolific sheep populations overlapped with 403 genes (Fig. 5B, Additional file 2: Table S27). Functional annotation of these genes revealed their important roles in the nervous system and the effect of oxytocin (e.g., *ADCY8*, *BMPR1B*, *GRID2*, and *PLCB1*) (Additional file 1: Fig. S13A, Additional file 2: Table S21). In goats, we detected 282 SV-genes under selection for the prolific phenotype (Fig. 5C, Additional file 2: Table S27). These candidate selected SV-genes showed important functions related to the development of animal organs and the nervous system (e.g., *BMPR1B*, *BMPR2*, and *GRID2*) (Additional file 1: Fig. S13B, Additional file 2: Table S21). We observed 19 genes (e.g., *BMPR1B*, *NELL1*, *CCSER1*, and *GRID2*) under convergent selection for the prolific phenotype in sheep and goats (Additional file 2: Table S22), which played essential roles in regulating follicular growth, embryo development, and litter size (e.g., *BMPR1B* and *GRID2*) (Additional file 2: Table S23) [39–41].

Interestingly, we identified 580 and 750 candidate genes for the prolificacy traits of sheep and goats, respectively, based on the top 1% of PBS values using genome-wide SNPs between the same prolific and non-prolific sheep/goat populations involved in the SV analysis (Additional file 2: Tables S28 and S29). Among the SNP-based candidate genes, 38 and 31 genes were overlapped with the SV-genes identified in sheep and goats respectively (Additional file 1: Fig. S14), implying that 365 and 251 SV-genes discovered here are novel candidates for fertility. Furthermore, the famous fecundity gene *BMPR1B* was only identified as the candidate gene for prolificacy traits in the SV-based selection tests of sheep and goats in our data (Additional file 2: Tables S27–S29), highlighting the important role of SVs in detecting the fecundity genes.

Similarly, we identified 272, 241, 287 and 205, 230, 261 SV-genes for the traits of wool/hair fineness, dairy, and meat in sheep and goats, respectively (Additional file 1: Figs. S15–S17, Additional file 2: Table S27), of which 16, 11, and 15 genes showed signals of convergent selection (Additional file 2: Table S22). These convergently selected genes appeared to play important roles in regulating the wool/hair (*DGKB*, *KCNIP4*, *COL21A1*) [42], dairy (*PLEKHA5*, *RALYL*, *FAM155A*) [43, 44], and meat traits (*GRM5*, *LRP1B*, *PHLPP1*, *THSD7A*) [45–47] (Additional file 1: Supplementary Results). Overall, our results provide compelling evidence for convergent selection on an array of SV-genes for the production traits of sheep and goats (Fig. 5D, Additional file 2: Table S22).

### Molecular parallelism of functional genes under convergent selection

There is non-consensus on the precise distinction between convergent and parallel evolution [48], and considering that the two genera correspond to different lineages, we regarded the similarities between sheep and goats as resulting from convergent evolution (e.g., convergent selection). At the molecular level, following Woodhouse and Hufford [49] we considered that molecular parallelism occurred when convergent traits are caused by modification of the same genes. It is worth exploring the occurrence of genes that were selected at the genome-wide scale to understand to what extent molecular parallelism was responsible for the convergent selection signatures between sheep and goats. By integrating all the common candidate genes identified above, we found a total of 79 orthologous genes under convergent selection in the sheep and goat genomes, which was significantly greater (*P* < 0.001) than the number expected by chance (Fig. 5E, Additional file 2: Table S22). These orthologous genes accounted for 5.07% (79/1559) and 7.29% (79/1083) of all the identified candidate selected genes in sheep and goats, respectively. This indicated that only a small proportion of candidate genes were parallel selected and related to molecular parallelism in sheep and goats. Among the 79 genes, 43 have been reported in previous scans of selection signatures during the domestication and improvement of sheep, goats, and other animals based on SNPs or CNVs (Additional file 2: Table S30), indicating important functional roles of the genes and their potential convergent selection across additional taxa of domestic animals.

Interestingly, the convergently selected orthologous genes appeared to be significantly enriched in identical pathways and GO terms associated with reproduction traits in sheep and goats, such as long-term depression, the estrogen signalling, oxytocin signalling, hippo signalling, and TGF-beta signalling pathway (Fig. 5G, Additional file 2: Table S23), which regulate estrogen production and reproduction traits through the hypothalamic-pituitary-gonadal (HPG) and hypothalamic-pituitary-adrenal (HPA) axis [50, 51]. The estrogen signalling pathway is well known for its primary roles in regulating the female reproductive cycle, and the hippo signalling pathway participates in the regulation of oocyte polarity, meiosis and development, and ovum maturation (Fig. 5G) [52–54]. In addition, the oxytocin signalling and TGF-beta/BMP signalling pathway interact with the estrogen and hippo pathway in the postfertilization process [53, 55], regulating embryo differentiation and development, fetus growth and maturation, and endometrium and uterus function during pregnancy (Fig. 5G) [55–58]. Of the genes that are under selection for reproduction trait (Additional file 2: Tables S22 and S27), we found three convergently selected orthologous genes (*BMPR1B*, *ADCY3*, and *GRID2*) functioned in the above signalling pathways, while 14 and 2 genes selected in the sheep and goats, respectively, were also active in these pathways (Fig. 5G). Notably, previous studies of these genes (e.g., *BMPR1B*, *PLCB1*, *CTNNA3*, and *BMPR2*) in GeneRIF (Gene References Into Functions) and MGI (Mouse Genome Informatics) database offered substantial phenotypical and knockout evidences for their crucial functions in reproductive system, such as oocyte meiosis, early embryonic development, and state of trophoblasts during placentation (Additional file 2: Table S31) [59–89]. Our proposed molecular representation of convergent pathways and genes could provide new insights into human selection on prolific traits and promote molecular breeding in livestock.

### Molecular analysis of *BMPR1B* and *BMPR2* deletions

**Fig. 6:**
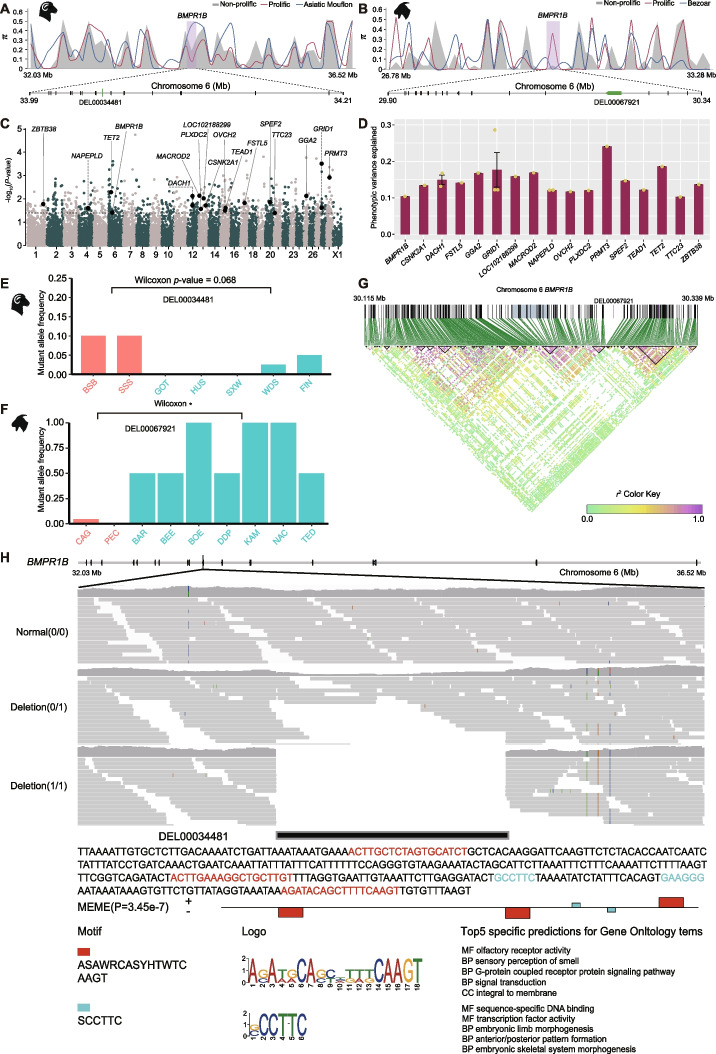
Evolution and functional analysis of the deletions in *BMPR1B*. **A**, **B** Nucleotide diversity across the *BMPR1B* locus and adjacent regions in sheep (**A**) and goats (**B**). The region of *BMPR1B* is shaded in light purple. **C** Manhattan plot of GWAS results for goat litter size. The horizontal dotted line represents the threshold of the top 5% of −log_10_(*P* values). The genes reported previously to be associated with reproduction are shown in the figure. **D** Phenotypic variance in goat litter size explained by the significantly (*P* < 0.05) associated SV loci and 17 annotated genes. The data are presented as the mean ± SD. **E** The distribution of the mutant allele frequency in deletion DEL00034481 of the *BMPR1B* gene is obviously different (Wilcoxon test, *P* = 0.0678) between prolific and non-prolific sheep populations. **F** The distribution of the mutant allele frequency in deletion DEL00067921 of the *BMPR1B* gene is significantly different (Wilcoxon test, *P* = 0.0429) between prolific and non-prolific goat populations. **G** Linkage disequilibrium and haplotype block analysis of the SVs and SNPs in *BMPR1B* of goats. The DEL00067921 deletion is not in linkage with the selected SNPs in the gene. The genomic regions under selection in the SNP analysis are shown in light blue. The SNPs located in and outside the haplotype blocks are indicated as gray and black lines, respectively. The DEL00067921 deletion is shown in purple. **H** IGV visualization of the location, sequence, and motifs of DEL00034481 in sheep *BMPR1B* and GO enrichment analysis of the motifs in DEL00034481

Among the convergently selected genes, we found two genes *BMPR1B* and *BMPR2* of the bone morphogenetic protein (*BMP*) family and the transforming growth factor β (*TGF-β*) superfamily, which were associated with the reproductive performance of sheep and goats during domestication and improvement. *BMPR1B* (*FecB*), one of the major functional genes affecting fecundity [39, 40], was found to be under convergent selection associated with fertility in sheep and goats (Fig. 5B, C, Additional file 2: Table S22). In *BMPR1B*, a 350 bp deletion (DEL00034481) within the intron located at chr6:34,034,777–34,035,127 in sheep and a 24,341 bp deletion (DEL00067921) located in the intron at chr6:30,214,561–30,238,902 in goats were identified to be under selection between prolific and non-prolific populations (Additional file 2: Table S27). We observed simple tandem repeats in the DEL00034481 deletion of sheep *BMPR1B* and many mobile elements (e.g., LINE/L1, SINE/MIR, DNA/hAT-Charlie, LINE/RTE-BovB) in the DEL00067921 deletion of goat *BMPR1B* from the UCSC genome browser. The deletions were clearly visualized through the IGV visualization (Fig. 6H, Additional file 1: Fig. S18B). We calculated nucleotide diversity in *BMPR1B* and its upstream and downstream regions, and observed a reduction in nucleotide diversity in DEL00034481 in prolific relative to non-prolific sheep populations but an increase of nucleotide diversity in DEL00067921 in prolific relative to non-prolific goat populations (Fig. 6A, B). The frequency distribution of the SV alleles showed nearly significant differences (Wilcoxon test, *P*_adj_ = 0.0678) between the prolific and non-prolific sheep and significant differences (Wilcoxon test, *P*_adj_ = 0.0429) between the prolific and non-prolific goat populations (Fig. 6E, F, Additional file 2: Tables S32 and S33). Notably, we found a peak at chr6:34,039,320–34,039,587 located within 5 kb of the downstream region of DEL00034481, indicating that the deletion potentially overlapped with enhancer region. PCR validation showed an average validation rate of 94.59% for the SV genotypes in the sheep populations (Additional file 1: Fig. S19, Additional file 2: Table S34).

**Fig. 7:**
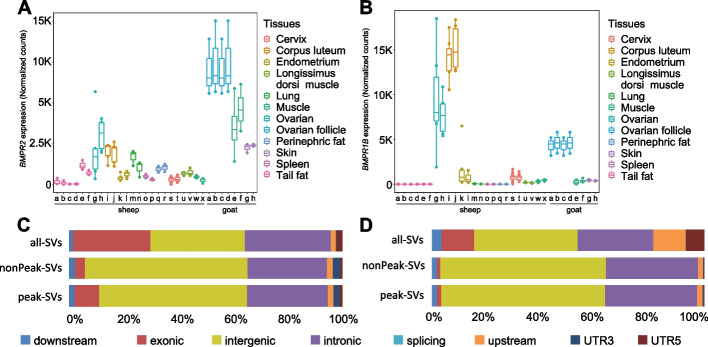
Impacts of SVs on regulatory elements. **A** The gene expression level of *BMPR2* across different tissues of sheep (a–x) and goats (a–h). **B** The gene expression level of *BMPR1B* across different tissues of sheep (a–x) and goats (a–h). **C** Comparisons of proportions between peak-SVs, nonPeak-SVs, and all SVs situated in different genomic regions of the domestic sheep genome. **D** Comparisons of proportions between peak-SVs, nonPeak-SVs, and all SVs situated in different genomic regions of the domestic goat genome. Please see Additional file 2: Tables S44 and S48 for the details of the pairwise group comparisons involved in the ATAC-seq and RNA-seq analyses

Phylogenic analysis showed that the DEL00034481 sequence within *BMPR1B* of sheep was also present in the Bovinae, Caprinae, Hippotraginae, Odocoileinae, and Cervinae genomes. The presence of the DEL00034481 sequence in both Cervidae and Bovidae suggested an earliest origin in the last common ancestor ~23 million years ago (Additional file 1: Fig. S20). We predicted two kinds of transcription factor binding motifs (ASAWRCASYHTWTCAAGT and SCCTTC) in the DEL00034481 sequence, which are functionally related to the sensory perception of smell, embryonic limb morphogenesis, and embryonic skeletal system morphogenesis (Fig. 6H). RNA-seq analysis indicated that the expression of *BMPR1B* in the ovary, corpus luteum, endometrium, and cervix of sheep was higher than that in the other tissues (Fig. 7B). Linkage disequilibrium analysis showed that the DEL00034481 deletion was closely linked to several adjacent selected SNPs in the same haplotype block, but was not in linkage (*r*^2^ = 0.052) with the causal SNP (c.A746G) reported for litter size at the 746 site of the coding region (Additional file 1: Fig. S21). These results suggest that DEL00034481 might introduce transcription factor binding motifs that increase the expression of *BMPR1B* in the reproductive tissues of sheep, which could be associated with the high fertility of prolific sheep independent of the effect of causal SNP. The DEL00067921 sequence in *BMPR1B* of goats occurs in the Bovinae, Caprinae, Hippotraginae, Odocoileinae, and Cervinae genomes. The presence of DEL00067921 in both Cervidae and Bovidae suggests its origin in the last common ancestor ~23 million years ago (Additional file 1: Fig. S18A). Two kinds of transcription factor binding motifs (GGAGGAGAAGGGGACRACAGAGGATGAGATGGYTGGATGGC and ATTTCATGGCTGCAVTCACCATCTGCAGTGATTTTGGAGCC) predicted in the DEL00067921 were associated with growth factor, spleen development, and myoblast fusion (Additional file 1: Fig. S18B). Transcriptome data showed that the expressions of *BMPR1B* in the ovarian follicle of goats were higher than in muscle and skin (Fig. 7B). Linkage disequilibrium analysis indicated that the DEL00067921 deletion was not in linkage with selected SNPs (Fig. 6G). These results imply that transcription factor binding motifs in DEL00067921 could decrease the expression of *BMPR1B* in the reproductive tissues of goats, which was probably associated with the high fertility of prolific goats independent of the effect of SNPs.

*BMPR2* was revealed to have been under convergent selection during domestication (Additional file 2: Table S22). In *BMPR2*, a 197 bp deletion (SV_w_15555) in the intron located at chr2:218,822,797–218,822,993 in sheep and an 85 bp deletion (DEL00018513) in the 3′ UTR located at chr2:44,898,207–44,898,291 in goats showed signatures of selection (Additional file 1: Fig. S10A, B, Additional file 2: Tables S20). We validated the two deletions based on IGV visualization (Additional file 1: Figs. S22C and S23C) and the genotypes of SV_w_15555, with an average validation rate of 93.75% according to PCR analysis (Additional file 2: Table S35). We demonstrated that *BMPR2* in goats could be a novel candidate gene only selected by SV analysis, because there were only SVs (e.g., DEL00018513) but no SNP under selection in the gene (Additional file 1: Fig. S24). The two deletions exhibited similar patterns to those observed in *BMPR1B* in sheep and goats (Additional file 1: Supplementary Results) in terms of variations in nucleotide diversity (Additional file 1: Figs. S22A and S23A), differences in SV frequencies between domestic populations and their wild ancestor, phylogenetic origins (Additional file 1: Figs. S22B and S23B), functions of the predicted transcription factor binding motifs (Additional file 1: Figs. S22C and S23C), and differential expression among tissues (Fig. 7A). In addition, we identified a peak at chr2:218,821,915–218,822,146 located within 1 kb of the upstream region of the deletion of *BMPR2* gene in sheep, implying that the deletion likely situated in enhancer region. Collectively, our results demonstrated the essential roles of deletions in *BMPR1B* and *BMPR2* in determining the high fertility of sheep and goats, providing new insights into the molecular mechanism of prolific traits.

### GWAS for the litter size trait in goats

In the association analysis of litter size and whole-genome SVs in Yunshang black goats (Additional file 2: Table S36), we identified 700 SVs significantly (*P* < 0.05) related to the litter size trait, which overlapped with 203 genes (Additional file 2: Table S37). In particular, we identified the DEL00067921 deletion in *BMPR1B* (Fig. 6C), supporting the major effect of the convergently selected *BMPR1B* gene and a previously hidden deletion in determining reproductive performance in goats [39]. We validated the DEL00067921 deletion in the Yunshang black goat population with IGV visualization (Additional file 1: Fig. S25). Moreover, we identified 14 additional significant (*P* < 0.05) genes (Fig. 6C) that are reported to be associated with reproduction, such as embryo death and infertility (*PRMT3*, *TET2*, and *ZBTB38*), egg laying performance (*GGA2*), and sperm vitality (*SPEF2* and *CSNK2A1*) (Additional file 2: Table S38) [90–104]. For the litter size of goats, the phenotypic variance explained by the SVs in the above 15 genes ranged from 10.4 to 24.1% (Fig. 6D, Additional file 2: Table S38).

### Wild introgression of SVs into domestic populations

The allele frequency distribution of SVs in domestic sheep/goat populations and their wild relatives revealed 95 and 36 candidate introgressed SVs in domestic sheep and goats, respectively (Additional file 1: Fig. S26, Additional file 2: Tables S39 and S40). These introgressed SVs were most observed in Bashibai sheep and Longlin goat, and most enriched on chromosomes 1, 2, 23, and 24 of the sheep genome and 2, 6, 13, and 14 of the goat genome (Additional file 1: Fig. S26).

The introgressed SVs in domestic sheep overlapped with 41 genes (Additional file 2: Table S41). These genes are functionally related to female fertility (*TRPC1*, *SYCP1*, *MACROD1*, and *SLC44A4*), immune function (*PAK5*, *ZYG11B*, and *TSPAN32*), and pigmentation (*BNC2*) [105–107]. In goats, the introgressed SVs intersected with 17 genes related to meat traits (*MATN2* and *RYR2*), reproduction (*ABCC1*, *SLC22A18*, and *FAM135B*), and pigmentation (*BEND7*) (Additional file 2: Tables S42 and S43) [108, 109]. The introgression signals of SVs from wild species may have influenced fecundity, skin color, immunity, and production traits of domestic sheep and goats.

### Impacts of SVs on regulatory elements

We collected 66 ATAC-seq data from Hu sheep and Tibetan sheep and 5 ATAC-seq data from Alpine goats to evaluate the effects of SVs on regulatory elements (Additional file 2: Table S44). The integrated analysis of ATAC-Seq and SV data showed that the percentages of Peak-SVs (i.e., SVs with at least 50% of length overlapping with peaks) in exon, upstream, downstream, and 5′ UTR regions were higher than those of nonPeak-SVs in sheep (Fig. 7C) and goats (Fig. 7D), suggesting stronger impacts of SVs on the open chromatin in exons and regulatory regions than in the other genic regions. Furthermore, as previous studies indicated that peak regions revealed from ATAC-Seq data can represent functional elements in the genome, peaks have been used to annotate promoter (or transcription start site) and putative enhancer [110, 111]. Taking advantage of the same sheep samples of ATAC-Seq data and whole-genome sequencing data (Additional file 2: Table S45), we investigated potential enhancer regions based on a combination of peaks and SVs. We identified a total of 706,742 peaks in sheep genome. After excluding 60,621 peaks annotated as promoters, there were 646,121 peaks as potential enhancers, in which 75,449 were overlapped with SVs (i.e., at least 50% of SV length). The 75,449 candidate enhancers could be annotated with Peak-SVs and considered as the most probable enhancer regions in sheep genome.

### Genome-wide associations of SVs and environmental variables

We performed genome-wide environmental association analysis (GWEAS) using the latent factor mixed model (LFMM) based on 22 environmental variables (EVs) and SVs in native sheep and goats. In sheep and goat landraces, we detected 1246 and 1455 significant SVs (*P*_adj_ < 0.05) intersecting with 558 and 632 genes, respectively. Among the significant genes, 45 were detected in both sheep and goats (Additional file 1: Fig. S27, Additional file 2: Tables S46 and S47). Significant associations were observed for the intronic deletions of *SYT1* (DEL00020254 at chr3:122,442,902–123,075,902 in sheep; DEL00052389 at chr5:8,236,497–8,861,733 in goats), *CPNE4* (DEL00007674 at chr1:280,850,768–281,427,050 in sheep; DEL00011900 at chr1:137,317,163–137,966,558 in goats), and *PCDH9* (DEL00053386 at chr10:41,893,112–43,073,231 in sheep; DEL00146347 at chr12:46,066,326–47,242,354 in goats) (Additional file 2: Table S46). *SYT1* functions in maintaining plasma membrane integrity under abiotic stresses such as high salt and freezing [112]. *CPNE4* is a clock-linked gene that helps regulate early and late migratory chronotypes in American kestrels [113]. *PCDH9* was identified to play an important role in the location adaptation of Mediterranean sheep and goats [114].

## Discussion

Sheep and goats are two small ruminant livestock species that provide products such as meat, wool, milk, and hide. Elucidating the similar patterns of genomic SV signatures during the domestication and improvement of these species provides important insights into the evolution of small ruminants and facilitates the future genetic improvement of these and other closely related livestock. In this study, we de novo assembled a chromosome-level reference genome of Asiatic mouflon—the wild ancestor of domestic sheep—for the first time. High-quality reference genome and third-generation sequencing data could improve SV detection. Here we used the third-generation sequencing reads from the assembly of Asiatic mouflon to obtain high accurate SVs, and explored the selection signatures for sheep domestication based on these assembly-derived SVs. The first chromosome-level assembly of Asiatic mouflon will be of great importance in deeply exploring the genetic mechanisms of sheep domestication. Also, the assembly will provide a valuable resource to advance our knowledge of speciation and chromosome evolution in the genus *Ovis*, and can be integrated with assemblies of other wild sheep and domestic sheep (Additional file 2: Table S2) to conduct the pangenome analysis of the ovine species. Nevertheless, recent advances in ultralong-read sequencing technology have enabled the achievement of telomere-to-telomere (T2T) genome assemblies in human and several model species [115, 116]. Since T2T technology can accurately assemble previously unresolved regions (PURs, such as centromeres, telomeres, and Y chromosome), we expect that future T2T assembly of Asiatic mouflon and domestic sheep will discover a plenty of novel SVs in PURs, which could provide completely new knowledge about the selection signatures, genomic variations, and functional genes associated with sheep domestication. On the other hand, we also took advantage of a comprehensive collection of available genomes to generate high-quality SV datasets in sheep and goats. The high number of included genomes in our study (532 sheep and 281 goats) could ensure to detect most of the SVs in the genome because the numbers of identified SVs were close to saturation with our sample size (Additional file 1: Fig. S28). Compared to the SVs (primarily CNVs) identified previously from SNP BeadChip and whole-genome sequences (Additional File 2: Tables S8 and S9), we identified a large number of previously undetected SVs, corresponding to increases of approximately threefold and sixfold over the sheep and goat SV datasets, respectively.

To minimize potential errors and obtain high-quality SVs from short-read sequencing data, we adopted the following strategies to ensure the reliable identification of SVs. First, we used only the genomes with a relatively high sequencing depth (e.g., > 15 ×) for SV detection. Second, we used three programs with different algorithms (Manta, Delly, and Smoove) to detect SVs independently and retained only the common SVs detected by at least two programs. Then, we merged the SVs across all the samples with SURVIVOR. Additionally, we kept only the SVs of < 1 Mb for SV characterization and downstream population genomics analysis. Finally, we validated randomly selected SVs and SVs in several well-known genes through molecular experiments and IGV visualization. Based on these strategies, we believe that the SVs identified here are reliable [117, 118] and that our integrated strategies are particularly suitable for SV identification from short-read data in a large number of samples. We identified much more deletions than other types of structural variants, consistent with the previous studies [8, 13]. This could be resulted from the use of a single reference genome assembly of sheep or goats for SV detection, in which the specific genomic architectures (e.g., the quantity and distribution of MEIs) may lead to a bias for detecting insertion, duplication, and inversion in our dataset from the short-read data. Nevertheless, long-read sequencing data, pangenome graph, T2T assemblies, and improved variant-calling algorithms will help to substantiate the SVs discovered here and exploit more long-length SVs and novel SVs in sheep and goats.

The population structures of goats and sheep (Additional file 1: Fig. S29), as revealed by PCA and phylogenetic tree, genetic structure, and linkage disequilibrium analyses based on genome-wide SNPs performed in this study and previous investigation [11], respectively, were generally consistent with the patterns revealed by SV analyses. This provides evidence that the SVs identified here could largely recapture the known population structures in sheep and goats. Nevertheless, the results from the SNP analyses could reveal clearer differentiation between wild progenitor and domestic populations (e.g., Asiatic mouflon and domestic sheep; bezoar and domestic goats) and a more refined structure within Asian populations (e.g., Central-and-East Asian, South-and-Southeast Asian and Middle-Eastern sheep; East Asian, South Asian and Southwest Asian goats) than those inferred from SVs (Additional file 1: Fig. S29) [11, 119]. This could be due to the larger number of SNPs than SVs across genomes, providing more informative alleles in genetic differentiation analyses.

We performed whole-genome tests to detect the selection signatures of SVs during the domestication and improvement of sheep and goats. In addition to previously reported genes (e.g., *BMPR1B*, *BMPR2*, *CPA6*, *FAF1*, *KLHL1*, *LEPR*, and *RORA* and *TMEM117*) identified based on SNP or SV analyses [11–13, 119], we revealed an array of novel genes (e.g., *ANK2*, *AUTS2*, *DGKI*, *NKAIN2*, *OPCML*, *PRIMPOL*, *RBFOX1*, *RGS7*, *TRAPPC12*, and *TSHZ2*) under selection, particularly for important production traits such as fertility, meat, dairy, and wool or hair fineness in sheep and goats. By illustrating the level of linkage disequilibrium between selected SVs and selected SNPs and revealing whether they are located in the same haplotype block in the candidate genes (e.g., *BMPR1B*, *BMPR2*), our results demonstrate that SVs are valuable molecular markers for revealing novel candidate genes for important traits that have remained undetected by SNP analyses. Notably, we revealed convergent signatures across the sheep and goat genomes from various selection tests and environment association analyses and identified molecular parallelism between the two species through a collective set of 115 commonly detected SV-genes. Based on the 115 genes, we proposed an integrated genomic view of convergent evolution based on the parallel selected genes with relevant functions (Fig. 5A). Particularly, we also constructed a genomic map for displaying molecular parallelism of 79 SV-genes underlying convergent signatures of selection (Fig. 5D). We validated the SVs in several parallel selected SV-genes that are known to be functionally associated with production or phenotypic traits using IGV visualization; these genes included *BMPR1B* and *BMPR2* for fertility; *CCSER1*, *LRP1B*, and *TMEM117* for meat; and *CNTNAP2* and *PRKG1* for dairy and meat (Fig. 6H, Additional file 1: Figs. S18B, S22C, S23C, and S30). Functional enrichment analysis of the 79 parallel selected genes showed their associations with reproduction, neural processes, and metabolism, which are possibly relevant to production traits and environmental adaptation. This set of common SV-genes greatly expands upon the set of candidate genes under convergent evolution identified earlier based on SNPs [12, 120] and SVs [13]. Taking the previous study on convergent genomic signatures of domestication in sheep and goats based on SNPs as an example [12], we identified much more common candidate selected genes (this study, 31 genes; previous study, 9 genes) for the domestication of sheep and goats, likely due to much larger sample size included in the present study, greater effect of SVs on phenotypic traits, and differences in the methods used (e.g., stringency of the detection) as compared with that of SNPs. Leveraging the 79 common candidate selected genes, we revealed that a limited extent (5.07% for sheep genes and 7.29% for goat genes) of molecular parallelism has occurred during the convergent evolution of sheep and goats, consistent with previous findings that parallelism was less detected than convergence [121]. This suggested parallel gene-use to some extent by sheep and goats and provided a remarkable example of molecular parallelism between ruminant species. Collectively, our findings provide more large-effect target genes that are potentially useful for future trans-species molecular breeding of sheep, goats, and other related livestock.

Among the 79 genes under convergent selection, *BMPR1B* and *BMPR2* have been reported to be the major functional genes associated with reproduction in pigs, sheep, and goats based on SNP analysis [24, 29, 114]. Here, the evidence of convergent selection on the two genes based on SVs further substantiated their important roles in regulating reproductive traits. In fact, associations between convergently selected genes and the same or similar phenotypic traits of related species have been observed in several crops and animals, such as *CHST11* and *SDCCAG8* for fertility in goats and sheep [120], *DYNC2H1* and *PCNT* for pseudothumb development in the giant panda and red panda [122], and *KRN2* and *OsKRN2* for grain yield in maize and rice [123]. Through integrated analyses of molecular evolution, GWAS, gene expression, and experimental validation data, we independently associated previously undetected SVs (e.g., deletions) in *BMPR2* and *BMPR1B* to generate a two-stage evolutionary pattern of reproduction traits in sheep and goats. The selection signatures found in *BMPR* genes during domestication and genetic improvement could reflect the ongoing selection on prolific traits in small ruminant livestock. Similar long-term selection on a particular trait at different evolutionary stages has been reported for fruit size in horticultural species such as tomato, apple, and cherry [124–126].

Furthermore, we found that SVs more frequently influence open chromatin in exons across sheep and goat genomes. The findings are in accordance with previous conclusions that SVs are often associated with phenotypic variation [127–129]. Based on the introgression analysis, we revealed that the SV contents in the genomes of domestic sheep (e.g., Bashibai sheep) and domestic goats (e.g., Longlin goat) were partly shaped by genetic introgression from their wild relatives. These findings add novel information to previous results of SNP analyses showing that genetic introgression has occurred between closely related species [11, 25, 130–132]. More interestingly, the common genes among the introgressed genes in domestic sheep and goats were associated with traits such as reproduction, body conformation, and pigmentation, providing rare examples of introgressed genetic materials showing the same functional roles between different species. In addition, the SVs associated with environmental variables in native sheep and goat populations were annotated to several common genes responsible for temperature adaptation, circadian clock regulation, and abiotic stress responses, providing valuable insights into common mechanisms of climatic adaptation across species.

Additionally, it should be noted that due to the limitation to access the samples of wild animals, this study could only include very small sample size (e.g., < 5 samples) for some wild sheep (e.g., urial, argali, European mouflon, and snow sheep) and wild goat species (e.g., Iberian ibex, Nubian ibex, and markhor) (Additional file 2: Table S1). Also, the sequencing depth of six wild goat samples including one Alpine ibex and five Siberian ibex was relatively low (e.g., < 10 ×) (Additional file 2: Table S1). Under this circumstances, we used the SV-calling programs (e.g., Manta, Delly, and LUMPY) which can precisely identify SVs on even an individual sample [133] or on samples with sequencing depth even below 4 × [134, 135]. Although the deficiency of samples in the aforementioned wild species may restrict the programs to detect all potential SVs in these species, it was unlikely to affect the main results of this study (e.g., assembly of Asiatic mouflon, SV characterization and selection signatures for domestic sheep and goats, and the impact of SVs on open chromatin and environmental adaptation) because of the non-involvement of these wild species in the relevant analyses. Regarding the difference in sequencing depth of our samples, we considered that it should not lead to obvious bias in SV characterization, because the sequencing depth of most (787/813) modern sheep and goat samples is higher than 15 × which is an adequate depth for accurate detection of SVs from short-read sequencing data [136]. For the potential batch effects in the collected genome sequencing data of sheep/goat samples, our strategies of data collecting (e.g., reducing the difference in genome sequencing depths of samples, with most samples > 15 ×), data processing (e.g., quality control for raw reads and alignments, SV calling with single sample and subsequently integrated SVs with all the samples), and data analyzing (e.g., quality control for analyzed SV data, robust analysis methods to control false positives and demographic factors) could have at least alleviated the batch effects in our integrated SV data and corresponding results base on previous investigations [137–139]. Future studies including a large number of high-depth genomes of wild sheep and goats will enable to comprehensively characterize whole-genome SVs and better resolve SV-associated questions for wild species in the genus *Ovis* and *Capra*.

## Conclusions

Leveraging a new created high-quality assembly of Asiatic mouflon and available high-depth (> 15 ×) genomes of worldwide ovine and caprine populations, we generated one of the most comprehensive resources of SVs in livestock and their wild relatives. We revealed convergent signals of SV-genes associated with domestication, improvement, adaptation, and production traits in the whole-genome landscape and provided an integrated genomic view of convergent evolution from wild progenitors to specialized populations in sheep and goats. In particular, we found strong evidence for the important roles of deletions in *BMPR1B* and *BMPR2* in regulating litter size traits and proposed novel molecular mechanism of neural regulation on the hypothalamic-pituitary-gonadal (HPG) axis underlying female reproduction traits. Our results highlight the utilization of SV markers to discover novel genes and genetic variants associated with evolutionary events and important traits that cannot be detected by SNP analyses, and suggest the potential to utilize trans-species SVs to accelerate the trait improvement of farm animals with modern techniques.

## Methods

### Whole-genome sequences

For sheep, goats, and their wild relatives, whole-genome sequences of modern samples and ancient remains were retrieved from publicly available data, including data from 281 modern [10, 119, 140–149] and 84 ancient samples [10, 21, 22] of *Capra* species and 532 modern samples [11, 24, 25, 150] of *Ovis* species (Additional file 2: Tables S1 and S5). In particular, the samples of domestic sheep and goats represent populations with various morphological and production traits such as fertility, wool or hair fineness, and dairy and meat production.

The samples of modern sheep populations and their wild relatives represented 37 animals from 7 wild sheep species (*O. aries*, *O. orientalis*, *O. musimon*, *O. nivicola*, *O. dalli*, *O. canadensis*, *O. ammon*, and *O. vignei*) and 495 animals from 129 worldwide domestic populations (95 landraces and 34 improved populations) (Fig. 1A, Additional file 2: Table S1). The samples of modern goats represented a global collection of 209 animals from 37 domestic populations (28 landraces, 6 improved, and 3 unassigned populations) and 72 animals from 6 wild goat species (*C. hircus*, *C. aegagrus*, *C. sibirica*, *C. nubiana*, *C. pyrenaica*, *C. ibex*, and *C. falconeri*) (Fig. 1A, Additional file 2: Table S1).

Genomic data of ancient remains of goats were retrieved from three recent studies [12, 13, 20], including samples from Asia, Europe, and the Middle East (Fig. 1A, Additional file 2: Table S5). All the sequences with sequencing depth > 15 × were selected in the wild and domestic goats (average 21.45 ×) and sheep (average 18.32 ×) (Additional file 2: Table S4). The sequencing depth of ancient goat genomes was 0.001–3.90 × (Additional file 2: Table S5).

### Whole-genome assembly of the Asiatic mouflon

The blood and biopsy samples from various tissues of Asiatic mouflon were taken and rapidly frozen in liquid nitrogen. These samples were then preserved on dry ice during transportation and stored at − 80 °C for future research purposes. Genomic DNA was isolated using a QIAamp DNA Mini Kit (QIAGEN). The integrity and concentration of DNA were measured with an Agilent 4200 Bioanalyzer (Agilent Technologies, Palo Alto, California). Eight micrograms of genomic DNA was sheared using g-Tubes (Covaris, Woburn, MA) and concentrated with AMPure PB magnetic beads (Pacific Biosciences). Each SMRTbell library was constructed using the SMRTbell Template Prep Kit 3.0 (Pacific Biosciences). The constructed libraries were size-selected on a BluePippin™ system for molecules ≥ 15 kb, followed by primer annealing and the binding of SMRTbell templates to polymerases with the DNA Polymerase Binding Kit. The sequencing of 8 SMRT cells was performed on the Pacific Bioscience Sequel platform with a movie length of 10 h by Annoroad Gene Technology Company (Beijing, China).

The filtered PacBio Sequel sequencing data were corrected by NextCorrect v2.5.0 (https://github.com/Nextomics/NextDenovo) using the parameters reads_cutoff:1 k and seed_cutoff:35 k and were assembled using NextGraph v2.5.0 (https://github.com/Nextomics/NextDenovo) with the default parameters. To further improve the assembly accuracy, one round of consensus correction was performed using Arrow v2.0.1 (https://github.com/PacificBiosciences/gcpp) with PacBio reads, followed by four additional rounds of consensus correction using NextPolish v1.0.5 [151] with Illumina reads. Illumina short-read data were generated using the Illumina HiSeq platform. To assess the completeness of the genome assembly, we searched the annotated genes in the assembly using the BUSCO package v5.4.7 [152]. BUSCO was also used to evaluate the completeness of the gene set on the basis of 13,335 highly conserved eukaryotic genes in the cetartiodactyla odb10 database. BioNano-based consensus mapping was performed by the hybrid scaffolding module in the IrysView package v2.5.1 (https://bionanogenomics.com/support/software-downloads/) with the manufacturer’s suggested parameters. The Hi-C sequencing data were first aligned to the assembled genome using Bowtie2 v2.4.5 [153] with the end-to-end read alignment model and were then clustered, ordered, and organized into pseudochromosomes using Lachesis [154]. Finally, the predicted pseudochromosomes were cut into 100-kb bins of equal length, which were used to construct a heatmap based on the interaction signals generated by valid mapped read pairs to manually validate and correct the pseudochromosomes.

### Genome annotation and synteny analysis

#### Repetitive element and noncoding RNA annotation

Repeats in the Asiatic mouflon genome were analyzed according to a strategy combining the construction of a specific repeated sequence database and the identification of repetitive element sequences based on the database using RepeatMasker [155] and RepeatModeler [156]. In detail, tandem repeats were first annotated by using GMATA v2.2 [157] and TRF v4.07b [158]. Next, we masked the tandem repeats before annotating the transposable elements. MITE-Hunter [159] and LTR_retriver v2.9.0 [160] were then employed to construct a TE.lib library to mask the genome before using RepeatModeler v1.0.11 [156] to construct a RepMod.lib library. After classifying the de novo repeat libraries with TEclass software v2.1.3 [161] and combining the libraries with the RepBase database version 25.04 [162], RepeatMasker v r1.331 [155] was applied to search for TEs. To obtain a reliable profile of noncoding RNA, we queried the Rfam database [163] using the program cmscan implemented in the software Infernal v1.1.2 [164]. Furthermore, we predicted tRNAs and rRNAs using tRNAscan-SE v2.0 [165] and RNAmmer v1.2 [166], respectively.

#### Gene annotation

The identification of protein-coding regions and gene prediction were performed by integrating a transcriptome sequencing database and ab initio and homology-based gene prediction methods. Ab initio gene prediction was conducted to predict the protein-coding genes with Augustus v3.3.1 [167]. For homology-based prediction, homologous proteins of goats, sheep, cattle, mice, and humans were downloaded from the NCBI database and aligned with the assembled genome to predict homologous genes. We performed homology-based gene structure prediction using GeMaMo v1.6.1 [168]. PASA v2.3.3 [169] was used to identify the alternatively spliced transcripts in the gene models based on transcriptome data without a reference assembly. Finally, the gene sets predicted from the above three methods were integrated by using EvidenceModeler (EVM) v1.1.1 [169] based on the relative weights (1:1:1) of the transcriptomic, homology-based and ab initio evidence.

The functional annotation of the predicted genes was achieved by using Blastp v2.7.1 [170] with the parameters “-evalue 1e-5, -max_target_seqs 1.” Specifically, the protein sequences of the predicted genes were aligned against the sequences in the NCBI nonredundant protein (NR), KOG [171], and SwissProt [172] databases. Gene Ontology terms and pathways were assigned to the predicted genes by analysis against the GO [173, 174] and KEGG [175] databases. Additionally, the predicted genes were annotated by defining protein domains and protein families using InterProScan v5.0 [176] and the Pfam database 35.0 [177] with the default parameters. The completeness of the gene set was evaluated on the basis of 4104 highly conserved eukaryotic genes in the mammaliania_odb9 database using BUSCO v5.4.7 [152].

### Read alignment of the sheep and goat genomic data

Read alignment of the sheep and goat genomic data followed the procedures described previously [11]. Specifically, Trimmomatic v.0.39 [178] was used to trim adaptors and low-quality sequences. The resulting clean reads were mapped to the sheep (*Oar_rambouillet_v1.0*, NCBI accession GCA_002742125.1) or goat (*ARS1*, NCBI accession GCA_001704415.1) reference genomes using the BWA-MEM (Burrows‒Wheeler Alignment-mem) algorithm v.0.7.17-r1188 [179] with the default parameters. Subsequently, alignments were transferred into BAM format by using SAMtools v.1.11 [180], and duplicates were removed using both SAMtools and GATK v.4.1.9.0 [181].

### Structural variant calling and annotation

To achieve high accuracy and sensitivity [182], an integrated strategy was applied to detect the SVs of the short-read alignments [11]. In detail, SVs were detected from each sample by using three independent tools, Smoove v.0.2.6 (https://github.com/brentp/smoove), Delly v.0.8.5 [134], and Manta v.1.6.0 [133], with the default parameters. Smoove integrates the best practices of LUMPY [135] and other associated tools; here, SVs were called for each sample by LUMPY and merged across all the samples by SVtools [183]. Then, the SVs were genotyped by SVtyper [184] and annotated with read coverage using Duphold v0.2.1 [185]. To reduce the false-positive rate, SV call sets identified by individual tools were integrated and then merged among all the sheep or goat samples using SURVIVOR v.1.0.7 [186] with the command line “SURVIVOR merge sample.txt 1000 2 1 1 0 50 sample_SURVIVOR.vcf” and “SURVIVOR merge samples.txt 1000 1 1 1 0 50 final.vcf”. Only the SVs ≤ 1 Mb detected by at least two tools were retained for further analysis.

Based on the genic regions overlapping with SVs, we annotated the identified SVs with ANNOVAR v.2020-06-07 [187] and classified the SVs into seven categories: intergenic region, intronic region, exonic region, 2 kb upstream and downstream region, 3′ UTR, and 5′ UTR. When one SV intersected two or more different genic regions, that SV was annotated and classified into different categories simultaneously. Additionally, we defined the genes annotated with SVs as “SV-genes” and the remaining genes as “nonSV-genes.”

Next, we implemented TE annotation for the SV datasets identified above. We first performed de novo annotation of TEs for the sheep (GCF_016772045.1) and goat (GCF_001704415.1) reference genomes using the EDTA package v2.0.0 [188] with parameters “--overwrite 1 --anno 1 --threads 24.” The TE libraries constructed by EDTA were analyzed in RepeatMasker v2.0.2 [155] using parameters “-cutoff 255 -frag 20000”. We then obtained the overlapped SVs from RepeatMasker and the SV datasets identified above, which were considered as these SVs generated by TEs. The length distributions of SVs and TEs were illustrated with ggplot2 v3.3.6 [189].

### Distribution of SV hotspots and their overlap with QTL regions

We detected SV hotspots using a method detailed previously [129]. In summary, we counted the number of SV breakpoints in 1-Mb windows with a 500-kb step size on each chromosome and ranked all the 1-Mb windows according to the number of SV breakpoints within each window. We then defined the windows with the top 10% of SV breakpoints as the SV hotspots and merged all of the continuous hotspot windows as “hotspot regions.”

We considered the 5-Mb region at the end of each chromosome arm to be the telomeric region [7, 190]. We counted the numbers of SV breakpoints within 1-Mb bins in the telomere regions and nontelomeric regions and used the Wilcoxon rank-sum test to examine the statistical significance of their differences (Additional file 2: Table S12). Chromosome X of goats was excluded from this analysis because of its incompleteness [191].

To discover the SVs associated with QTLs, we compared the SVs with the QTLs of sheep and goats obtained from the Animal Quantitative Trait Loci Database (https://www.animalgenome.org/cgi-bin/QTLdb/index) [192]. Since only SVs shorter than 1 Mb were considered here, we excluded QTL regions larger than 5 Mb in the comparisons. We then identified the SVs in which at least 50% of the segments overlapped with QTL regions [26, 193] using the program BEDtools v2.29.1 with the parameter “-f 0.5 –F 0.5 –e” [194]. We evaluated the fold enrichment of the deletions in QTLs.

### Identification of novel SVs

To identify novel SVs, we compared the SVs identified here with the published datasets (Additional file 2: Table S8) [7, 24, 26, 193, 195–202]. Since previously identified SVs were called based on different reference genomes of *Ovis* species, we first converted the genome coordinates of previously published SVs to the coordinates in *Oar_rambouillet_v1.0* using the NCBI Genome Remapping Service (https://www.ncbi.nlm.nih.gov/genome/tools/remap). We used a 30% reciprocal overlap ratio (with the parameter “-f 0.3 -F 0.3 –e”) as a threshold to determine whether two SVs were the same variant, irrespective of whether the SVs were obtained from different sequencing technologies/platforms, different calling methods, or different reference genomes [7]. Similar procedures were implemented in *Capra* species with the exception of remapping analysis because all the SV datasets of the *Capra* species called here and previously were based on the same goat reference genome, *ARS1*. Finally, we detected novel SVs using BEDtools v2.29.1 with the parameter “-f 0.3 -F 0.3 –e -v.”

### Genetic diversity and population structure analysis

To assess genetic diversity levels, measures of nucleotide diversity (*π*) were calculated using VCFtools v.0.1.16 [203] with a sliding window size of 10 Mb across genomes. Heterozygosity was also estimated using VCFtools based on the proportion of heterozygous SV sites in the whole set of SVs. Pairwise genome-wide *F*_ST_ values were calculated among species based on the individual SV sites using VCFtools. Estimates of linkage disequilibrium (LD) parameter *r*^2^ were calculated between pairwise SVs within each chromosome using PLINK v.1.90b6.21 [204] with the parameters “–ld-window-r2 0 –ld-window 99,999 –ld-window-kb 300.” The results were plotted using ggplot2.

Population structure was explored based on the SV datasets. SVs with a missing genotype rate > 0.25 were excluded from the analysis using PLINK with the parameter “--geno 0.25”. After filtering, genetic structure was analyzed by using an unsupervised clustering algorithm implemented in ADMIXTURE v.1.3.0 [205], with the number of predefined genetic clusters (*K*) ranging from 2–5 for sheep and 2–6 for goats. Principal component analysis (PCA) was performed using GCTA v.1.93.2 [206]. Individual-level neighbor-joining (NJ) trees were constructed with 1000 bootstrap replicates using Phylip v.3.697 [207] based on the pairwise *p*-distance matrix. The NJ trees were rooted with Bighorn sheep or Siberian ibex, and both trees were visualized by using iTOL (https://itol.embl.de/) [208].

To reveal the difference in genetic differentiation inferred based on SVs and SNPs, the population structure of goats and sheep was also investigated based on genome-wide SNPs. In all 281 wild and domestic goats, PCA was performed with PLINK, and genetic structure was examined using ADMIXTURE with the default settings. The number of assumed genetic clusters (*K*) was set as 2–11. An individual-based NJ tree was constructed based on the nucleotide *p*-distance matrix using the program MEGA v.11 [209], and the final concordant tree was visualized using FigTree v.1.4.4 (http://tree.bio.ed.ac.uk/software/figtree/). LD among SNPs was calculated as above. The PCA, genetic structure, NJ tree, and LD of the 532 wild and domestic sheep based on SNPs was obtained from our recent study [11].

### Identification of functional genes under convergent selection

We detected the SV signatures of selective sweeps during the stages of domestication, early development, and later genetic improvement. We filtered the SV data with MAF < 0.01 and missing genotypes > 25% and retained high-quality SVs for the following analyses. For the domestication-stage analysis, we first aligned the Asiatic mouflon assembly (*CAU_Oori_1.0*) to the sheep assembly (*Oar_rambouillet_v1.0*) using the sequence aligner nucmer in the program MUMmer v4.0.0rc1 [210] and called SVs with Assemblytics v1.2.1 [211]. We genotyped the SV calls obtained from the program Assemblytics v.1.2.1 in the genomes of 33 Asiatic mouflon and 63 domestic sheep from the Middle East following the SV genotyping pipeline from https://github.com/GaoLei-bio/SV [212]. Then, we calculated Weir and Cockerham’s *F*_ST_ [213] for all the SVs across the genomes between the Asiatic mouflon and domestic sheep populations using VCFtools v0.1.16. To estimate the statistical significance of each *F*_ST_ value, we generated 200 randomly sampled *F*_ST_ values and obtained *P* value by measuring the proportion of random *F*_ST_ values higher than the observed *F*_ST_ value [214]. We also computed a differentiation index DI_SV_ between the domestic sheep and Asiatic mouflon populations, which can reflect the difference in derived allele frequencies between these populations [13]. To define the derived allele states when calculating DI_SV_, the ancestral allele genotype was estimated based on the majority allele of Asiatic mouflon. The SVs with the *P* values of *F*_ST_ < 0.05 and the top 5% of DI_SV_ values were identified as candidate selected SVs during domestication [19]. Similarly, we estimated *F*_ST_ and associated *P* values and DI_SV_ values for the SVs in the 18 bezoar and 168 native goats and identified candidate selected SVs during goat domestication. Using publicly available ancient goat genomes, we also identified candidate selected SVs with the top 5% of *F*_ST_ values during domestication and subsequent early development, which was performed between 18 bezoar and 84 ancient goats and between 84 ancient goats and 168 native goats. All SVs were included in the analyses due to small number of SV sites in ancient goat genomes.

Additionally, global *F*_ST_ estimates were calculated among 33 domestic goat populations as well as among 129 domestic sheep populations with VCFtools following previous approaches [19, 215]. In detail, we calculated *F*_ST_ values between each breed and all other breeds, and the average of the *F*_ST_ values was assigned as the global *F*_ST_ values for each SV. The SVs with the top 5% of global *F*_ST_ values were considered as candidate selected SVs associated with genetic improvement in the past several hundred years. Additionally, we used pbscan v2020.03.16 [216] to conduct selection tests for the specific traits of reproduction, wool/hair fineness, dairy, and meat production separately. The argument of pbscan was set as “-div 1.” The Asiatic mouflon and bezoar were used as outlier group for sheep and goats, respectively, and pairwise groups of sheep/goat populations with contrasting phenotypes were chosen for selection analyses (Additional file 2: Table S26). The SVs with the top 5% of PBS values were identified as candidate selected SVs for each trait. We then annotated the SVs and overlapped the goat and sheep candidate selected genes as parallel evolution for each phenotype. Candidate functional genes were annotated for the selected SVs using the program VEP release v.104.3 [217]. KEGG pathways and GO terms were enriched for the candidate genes with gprofiler v0.2.1 [218], and false discovery rate (FDR) was also computed in this program with the Benjamini–Hochberg method to correct for multiple testing.

To investigate the novelty of identified SV variants and genes under selection, we performed genome-wide selective sweep test for reproduction traits based on the SNP data of the same prolific and non-prolific sheep/goat populations involved in the SV analysis, and compared the results from SNPs and SVs. The SNP data of sheep were obtained from our previous study, and the SNPs of goats were called with the same pipeline as that used in sheep [11]. We filtered the SNP data with MAF < 0.05, Hardy-Weinberg equilibrium value < 0.001, and missing genotypes > 10%, and retained high-quality SNPs for the following analyses. We used the same method and populations as the selection test for reproduction traits based on SVs, except that the argument of pbscan was set as “-win 50 -step 25” and the top 1% of PBS values was considered as the criterion for choosing candidate selected regions. We conducted linkage disequilibrium analysis within candidate genes using the selected SNPs and SVs identified in the selective sweep tests. The linkage disequilibrium heatmap was generated from VCF files by LDBlockShow v1.40, with the parameter “-SeleVar 2.”

In the selective sweep tests, the commonly selected SV-genes in sheep and goats were identified to undergo convergent selection. A permutation test was used to determine whether convergent SV-genes were obtained more frequently than expected by chance, and statistical significance was evaluated by comparing the number of SV-genes observed based on real data with those randomly generated from the permutation test [123]. Enrichment analyses of GO terms and KEGG pathways [173–175] were carried out for the 79 convergently selected genes using gprofiler v0.2.1. The statistical significance of the enrichment results was calculated with the g:Profiler g:SCS algorithm for multiple-testing correction with a threshold of *P* < 0.05.

### GWAS for the litter size trait

An association analysis of litter size and SV variants in goats was performed using the MLM in GEMMA v.0.98.3 in Yunshang black goats (40 ewes) with detailed multigenerational litter size records (Additional file 2: Table S36) [219]. A total of 14,105 SVs were identified and used in the association analysis. The effect of population stratification was corrected by adjusting the first three principal components (PCs) estimated with PLINK v.1.90b6.21 [204]. The SVs with the top 5% of *P* values were considered to be significantly associated with litter size. The proportion of variance explained by the identified SV loci was calculated using the following formula:

1 $$\frac{2{\widehat{\beta }}^{2}MAF(1-MAF)}{2{\widehat{\beta }}^{2}MAF\left(1-MAF\right)+{2N\left(se\left(\widehat{\beta }\right)\right)}^{2}MAF(1-MAF)}$$

In the formula (1), $\widehat{\beta }$, *se*($\widehat{\beta }$), *MAF*, and *N* are effect size estimate, standard error of effect size, minor allele frequency, and sample size for the genetic variant, respectively [220].

### Evolutionary and functional analyses of deletions in *BMPR1B* and *BMPR2*

The deletions in *BMPR1B* and *BMPR2* of sheep and goats were visualized and validated with IGV 2.14.0 [221]. Additionally, we examined nucleotide diversity (*π*) in the genic and adjacent regions of *BMPR2* and *BMPR1B* in wild ancestors and domestic populations. The *π* value was calculated using the following formula with VCFtools v0.1.16:

2 $$\pi =\frac{n}{n-1}{\sum }_{ij}{x}_{i}{x}_{j}{\pi }_{ij}$$

In the formula (2), ${x}_{i}$ indicates the frequencies of the *i* th sequences, ${x}_{j}$ indicates the frequencies of the *j* th sequences, ${\pi }_{ij}$ indicates the number of allele differences per SV locus between the *i* th and *j* th sequences, and *n* indicates the number of sequences in the samples.

For organisms in the taxon Ruminantia, the sequences of the deletions along with the 1000 bp upstream and downstream regions in the *BMPR1B* and *BMPR2* genes of sheep and goats were subjected to BLAST searches against the NCBI RefSeq Representative Genome and Whole-Genome-Shotgun contigs (WGS) Database using NCBI blastn [222]. Assemblies covering the whole length of the query sequence with > 90% identity were considered to contain the deleted sequences, which was further confirmed by visualization. Phylogenetic trees were then constructed for each deletion in *BMPR1B* and *BMPR2* using iqtree2 v2.2.0 (-B 1,000) [223]. Additionally, motifs in the deleted sequences were identified by MEME v5.5.0 [224], and GO terms of the motifs were enriched by GOMo v5.5.0 [225]. Moreover, publicly available RNA-Seq data of sheep and goats were downloaded from EBI-ENA to compare the expression of *BMPR1B* and *BMPR2* across different tissues (Additional file 2: Table S48) [24, 226–235]. To standardize the RNA-Seq data from different studies, only the datasets meeting the following criteria were selected and included in the association tests: (i) paired-end (PE) reads, (ii) available information on breed/species name, tissue type, and sex, (iii) more than two species/populations for a tissue, and (iv) more than three biological replicates for a tissue of a species/population from the same BioProject. Thereafter, Trimmomatic v.0.39 was used to trim adaptors and low-quality sequences of the raw data. After filtering, high-quality data were processed using the HISAT 2.2.1 [236], featureCounts 1.6.0 [237], and DESeq2 1.42.1 [238] pipelines. In summary, the clean reads were mapped to the most updated goat (ARS1, RefSeq: GCF_001704415.1) or sheep reference genome (Oar_rambouillet_v1.0, RefSeq: GCF_002742125.1) using HISAT2 with the default parameters. Alignments were converted to BAM format by SAMtools v.1.11. Then, read counts of each gene were calculated from the BAM files by featureCounts, and FPKM (fragments per kilobase of exon per million fragments mapped) value was used to standardize the level of expression for each gene using an in-house script based on the read count tables.

### Analysis of introgression from wild relatives to domestic populations

We compared domestic sheep/goat populations with their congeneric wild relatives to search for potentially introgressed SVs using a method for SV-based introgression analysis in previous studies [239, 240]. For each domesticated population (88 native sheep populations and 14 native goat populations) and each wild species (7 wild sheep species and 6 wild goat species), we used VCFtools v.0.1.16 [203] to calculate the allele frequency of each SV with the option “-freq.” The candidate introgressed SVs from wild species to domestic populations were identified based on the following criteria: (i) SV was specific to one domestic population and fixed in any wild species (allele frequency = 1), but was absent from any other domestic populations (allele frequency = 0); (ii) allele frequency of SV should be zero in bighorn sheep (*Ovis canadensis*) for sheep [11] and Siberian Ibex (*Capra sibirica*) for goats [241], so as to exclude the candidate introgressed SVs due to common ancestor; (iii) each domestic population included in the introgression analyses should have at least 2 samples and each introgressed population should have more than 2 candidate introgressed SVs, so as to exclude the potential influence of genetic drift on the results.

### Tests for the impact of SVs on ATAC peaks

We utilized the published ATAC-Seq data of hypothalamus, rumen, heart, lung, liver, duodenum, spleen, and adipose of sheep from NCBI-SRA and liver parenchyma and alpha-beta cytotoxic T cell of goats from EBI-ENA to explore the impacts of SVs on the regulatory regions (Additional file 2: Table S44) [242, 243]. Particularly, all the ATAC-Seq data of sheep or goats are from the same project, thus batch effect should not exist in these data. Also, whole-genome sequencing data of the same sheep samples as the ATAC-Seq data are also publicly available (Additional file 2: Table S45) [243], which enabled us to obtain corresponding SVs to test the impact of SVs on ATAC peaks. Raw reads of ATAC-Seq data were trimmed with Trimmomatic v.0.39, and clean reads were then aligned to the sheep (Oar_rambouillet_v1.0) or goat (ARS1) reference genome using BWA-MEM. The resulting SAM files were converted into BAM files with SAMtools, and duplicates were filtered out by GATK. Then, the peaks were called for each sample from the BAM files using MACS2 v2.2.7.1 [244] with the options “-q 0.05 --nomodel --shift 100 --extsize 200 --keep-dup all --call-summits.” To obtain an integrated set of peaks in a population, the overlapped peaks in different samples were merged using BEDTools. Meanwhile, we extracted common SV sites among the populations involved in the ATAC-Seq analysis. We defined SVs with at least 50% of their length overlapping with the merged peaks as “Peak-SVs” and the remaining SVs as “nonPeak-SVs.” We then annotated the Peak-SVs and nonPeak-SVs by ANNOVAR and compared the percentages of Peak-SVs and nonPeak-SVs located in each of the annotated genic elements (e.g., exon, intron, upstream).

### Genome-wide environmental association study

For the geographic location of each native sheep/goat population, a total of 22 environmental variables (EVs) of 19 bioclimatic variables (bio1 – bio19) and three climate variables (elevation, solar radiation, and water vapor pressure) with their average values for 1970–2000 were retrieved from the World Climate database v2.1 (WorldClim) (https://www.worldclim.org/data/worldclim21.html) with a resolution of 2.5 arc-minutes. The genotype matrix of each individual containing the SVs with MAF > 1% and missing rate < 25% were also preprocessed with PLINK v.1.90b6.21 [204]. Next, the GWEAS was performed in 23,306 SVs from 358 native sheep and 32,689 SVs from 168 native goats using the LFMM method. The LFMM method was performed with PC1 (0.54 for sheep and 0.69 for goats) obtained from PCA of the 22 EVs and the above preprocessed SVs using R package lfmm v2 [245] with the parameters of “model lasso and -K 6.” The candidate SVs associated with environmental variables were identified based on the thresholds of *P*_adj_ < 0.05 in the LFMM analysis.

### Experimental validation of SVs

Polymerase chain reaction (PCR) and quantitative real-time PCR (qPCR) experiments were performed to validate the identified SVs. Seventeen DELs and 6 DUPs of sheep were randomly selected from the SV dataset, which were located in the common SV-genes of sheep and goats. Primers for the tested DELs and DUPs were designed using the program Primer Premier v6.00 (Additional file 2: Table S10).

PCRs were performed in a total volume of 20 μL containing 1 μL of DNA, 10 μL of 2 × Taq PCR MasterMix (GeneBetter Biotech, Beijing, China), 0.8 μL of forwards and reverse primers, and 7.4 μL of water. PCRs were performed on an Eppendorf Mastercycler (Eppendorf) under the following cycling conditions: 94 ℃ for 3 min, followed by 35 cycles at 94 ℃ for 30 s, 56 ℃ for 30 s, and 72 ℃ for 1 min, and then a final extension at 72 ℃ for 5 min. All of the amplification products were examined by 1.5% agarose gel electrophoresis (AGE). The lengths of the PCR products obtained from electrophoresis were compared with those inferred by a structural variant calling pipeline from the same individuals.

For qPCR, *DGAT2* was used as the internal reference gene [24–26]. qPCR was performed on a QuantStudio™ 1 Real-Time PCR System (96-Well 0.2 ml Block) (Applied Biosystems) using SYBR Green I fluorescence. qPCR was implemented in a total volume of 20 μL, which contained 2 μL of DNA, 1 μL of forward and reverse primers, 10 μL of SYBR qPCR Master Kit (2 ×), 0.4 μL SYBR ROX Low (50 ×), and 5.6 μL of water. The cycling conditions were set as 95 ℃ for 5 min, followed by 40 cycles at 95 ℃ for 10 s, 56 ℃ for 20 s, and 72 ℃ for 30 s, and the melting curve was set at 95 ℃ for 15 s, 60 ℃ for 1 min, and 95 ℃ for 15 s at the end of the amplification. Three replications were performed for all the samples and blank controls. Thereafter, the ΔΔ*C*_T_ method was used to validate copy number variations (CNVs), which are a particular subtype of SVs, using the equation ΔΔ*C*_T_ = (*C*_T_target_ − *C*_T_DGAT2_)_sample_A_ − (*C*_T_control_ − *C*_T_DGAT2_)_sample_B_, where *C*_T_ is the cycle threshold, sample A is the test individual and sample B is the control individual [246]. ΔΔC_T_ values between 1.414 and 2.449 were inferred to indicate a normal copy number of 2 [246]. Accordingly, the concordance between the called CNVs and the relative copy numbers estimated based on qPCR was evaluated.

### Comparison of common candidate genes with previous bibliography

To substantiate the functions of the 79 common candidate selected genes identified in sheep and goats, we investigated whether these genes have been reported previously in human and other animals. Specifically, we conducted an extensive literature review by searching online database, such as “Web of Science” and “China National Knowledge Infrastructure,” using keywords “gene symbol (e.g., *BMPR2*) and domestication,” “gene symbol (e.g., *BMPR2*) and particular trait (e.g., meat, wool or milk), etc. Occasionally, we also included specific animal (e.g., sheep, goats or cattle) as an additional keyword to refine the results. We then summarized all the previously reported genes in terms of function and animal in Additional file 2: Table S30 [28, 30, 31, 35–38, 41, 43–47, 103, 114, 247–306]. To eliminate potential bias in the results of literature review, we did not use the literature including the same sheep/goat individuals as the present study for gene comparison.

### Supplementary Information

Additional file 1: Supplementary Results and Supplementary Figures 1–30. Fig. S1. Genomic characterization and synteny landscape for the *de novo* assembled Asiatic mouflon genome. Fig. S2. Venn diagrams. Fig. S3. The size distribution of SVs exceeding 1 kb by variant types in sheep and goats. Fig. S4. Experimental validation of the SVs identified in this study. Fig. S5. Genomic features of the sheep and goat genomes. Fig. S6. Distribution of short length SVs and transposable elements. Fig. S7. The pattern of linkage disequilibrium (LD) decay in genomes of sheep and goats based on SNPs. Fig. S8. Principal component analysis (PCA) of domestic sheep and domestic goats based on structural variants. Fig. S9. The cross-validation errors for different values of *K* in admixture analysis of sheep and goats. Fig. S10. Genome-wide selective test for candidate genes associated with the domestication of domestic sheep and domestic goats. Fig. S11. Genome-wide selective test for candidate genes associated with the domestication of domestic goats during two different developmental stages. Fig. S12. Genome-wide selective test for candidate genes associated with the genetic improvement of domestic sheep and domestic goats. Fig. S13. Functional enrichment analysis reveals significant GO terms and KEGG pathways for the candidate selected genes associated with the fertility. Fig. S14. Genome-wide selective test for candidate genes associated with the reproduction traits of domestic sheep and domestic goats based on SNP data. Fig. S15. Genome-wide selective test for candidate genes associated with the wool and cashmere related trait of domestic sheep and domestic goats. Fig. S16. Genome-wide selective test for candidate genes associated with the dairy trait of domestic sheep and domestic goats. Fig. S17. Genome-wide selective test for candidate genes associated with the meat production trait of domestic sheep and domestic goats. Fig. S18. Evolution and function analysis of the deletions in *BMPR1B *of goats. Fig. S19. Experimental validation of the selected deletions in *BMPR1B* and *BMPR2*. Fig. S20. Evolution analysis of the deletions in *BMPR1B *of sheep. Fig. S21. Linkage disequilibrium and haplotype block analysis of the SVs and SNPs in *BMPR1B *of sheep. Fig. S22. Evolution and function analysis of the deletions in *BMPR2 *of sheep. Fig. S23. Evolution and function analysis of the deletions in *BMPR2 *of goats. Fig. S24. Linkage disequilibrium and haplotype block analysis of the SVs and SNPs in *BMPR2 *of goats. Fig. S25. The IGV visualization of location and sequence of the deletion in *BMPR1B* significantly (*P* < 0.05) associated with litter size in Yunshang black goats. Fig. S26. SV introgression from wild sheep to domestic sheep populations at chromosomes 1–27 and from wild goat to domestic goat populations at chromosomes 1–29. Fig. S27. Genome-wide environmental analysis of 22 environmental variables and structural variants associated with local adaptation in sheep and goats. Fig. S28. The SV numbers identified in the *Ovis* and *Capra* are close to saturation with our sample sizes. Fig. S29. Genetic structure of wild and domestic goat populations based on SNPs. Fig. S30. The IGV visualizations of locations and genotypes of the SVs in 5 well-known genes associated with production traits.

Additional file 2: Table S1. Summary information of 495 domestic and 37 wild sheep as well as 209 domestic and 72 wild goats used in this study. Table S2. Statistic for wild and domestic sheep assemblies. Table S3. The distribution of GC content and gene coverage and their correlation in the genome assembly of Asiatic mouflon. Table S4. Summary information of the sequencing and alignment statistics for sheep (*n* = 532) and goat (*n* = 281) samples. Table S5. Summary information of ancient goat DNA samples used in this study. Table S6. Summary information of SVs in sheep (*n* = 532) and goats (*n* = 281). Table S7. Length distribution of SVs in the predefined ranges in sheep and goats. Table S8. Comparison of the SVs/CNVRs identified in this study and those reported in previous studies. Table S9. The novel SVs/CNVRs that were not detected in previous studies. Table S10. Details of SV validation by PCR and qPCR in sheep. Table S11. The SV hotspot regions overlapping with QTL regions in sheep and goats. Table S12. The number of SVs in the telomere and normal regions in sheep and goat genomes. Table S13. Functional annotation of the SVs detected in the 532 domestic and wild sheep. Table S14. Functional annotation of the SVs detected in the 281 domestic and wild goats. Table S15. SV annotated genes that are common between *Ovis* and *Capra*. The SVs associated with the common genes are either specific to or shared between *Ovis* and *Capra* species. Table S16. Summary information of the overlapped SVs and QTLs in sheep and goats. Table S17. Log_2_(fold enrichment) of SVs in QTLs for each individual of sheep. Table S18. Log_2_(fold enrichment) of SVs in QTLs for each individual of goats. Table S19. Transposable element (TE) annotation in *Ovis* and *Capra* SV calling sets. Table S20. Functional annotation of candidate selected regions identified with overlap of top 5% DI_SV_ and significant *F*_ST_ for domestication related traits in domestic sheep and domestic goats. Table S21. The most significantly enriched GO terms (FDR < 0.1) and KEGG pathways (FDR < 0.1) for the candidate genes associated with domestication, improvement, wool, milk, meat and reproduction related traits in domestic sheep and domestic goats. Table S22. Common genes under convergent selection between sheep and goats associated with domestication, improvement, wool, milk, meat and reproduction related traits. Table S23. Functionally enriched (FDR < 0.15) GO terms and KEGG pathways for the common genes under convergent selection associated with domestication, improvement, wool, milk, meat and reproduction related traits in sheep and goats. Table S24. Functional annotation of candidate selected regions identified with the top 5% *F*_ST_ value for stage I (from bezoar to ancient goats) and stage II (from ancient goats to native goats) of goat domestication. Table S25. Functional annotation of candidate selected regions identified with the top 5% *F*_ST_ value for improvement related traits in domestic sheep and domestic goats. Table S26. Detailed information on sheep and goat populations involved in the selective tests for structural variants (SVs) associated with particular production traits. Table S27. Functional annotation of candidate selected regions identified with the top 5% PBS value for wool, milk, meat and reproduction related traits in domestic sheep and domestic goats. Table S28. Functional annotation of candidate selected regions identified with the top 1% PBS value for reproduction traits in domestic sheep based on SNP data. Table S29. Functional annotation of candidate selected regions identified with the top 1% PBS value for reproduction traits in domestic goats based on SNP data. Table S30. Convergently selected genes of sheep and goats identified in this study that have been reported previously in human, sheep, goat and other animals. Table S31. Candidate selected genes in the convergent pathways implicated in the female reproduction process in Fig. 5 that have been previously reported with functions in reproductive system by studies in GeneRIF (Gene References Into Functions) and mouse transgenic document in MGI (Mouse Genome Informatics). Table S32. Allele frequency of the deletion (DEL00034481) in the* BMPR1B* gene in prolific and non-prolific sheep populations. Table S33. Allele frequency of the deletion (DEL00067921) in the *BMPR1B* gene in prolific and non-prolific goat populations. Table S34. Experimental validation results of *BMPR1B* gene in prolific and non-prolific sheep. Table S35. Experimental validation results of *BMPR2* gene in Asiatic mouflon and the Middle Eastern domestic sheep populations. Table S36. Whole-genome sequencing and phenotypic data of goats used in this GWAS analysis for the litter size trait. Table S37. Genome-wide association signals using SV data for the litter size trait of goats based on the threshold of *P* < 0.05. Table S38. Overlapped genes between those identified by GWAS analysis of goats in this study and reported previously to be functionally linked to reproduction in human, sheep, goat and other animals. Table S39. Candidate introgression regions identified in domestic sheep based on the allele frequency of specific SVs that shared with wild sheep. Table S40. Candidate introgression regions identified in domestic goats based on the allele frequency of specific SVs that shared with wild goats. Table S41. Genes overlapped with the introgression regions in domestic sheep. Table S42. The most significantly (FDR < 0.1) enriched results of gene ontology terms and KEGG pathways for the introgressed genes identified in domestic sheep and domestic goats. Table S43. Genes overlapped with the introgression regions in domestic goats. Table S44. The sheep and goat samples of the ATAC-seq data used in this study. Table S45. The whole-genome sequencing data of the same sheep samples as the ATAC-seq data. Table S46. Significant signals of SVs identified by genome-wide association study (GWAS) between SVs and environmental variables in sheep and goats. Table S47. The most significantly enriched GO terms (FDR < 0.1) and pathways (FDR < 0.1) for the candidate genes associated with environmental variables in sheep and goats and overlapped genes in both ruminants. Table S48. The sheep and goat samples of the RNA-seq data used in this study.

Additional file 3. Review history.

## Data Availability

The assembly and annotation of CAU_Oori_1.0 have been deposited to the NCBI Genome database under the accession number GCA_014523465.1 [307]. Raw sequencing reads from Illumina, PacBio, Hi-C, Iso-Seq and RNA-seq and Bionano data generated in this study for assembling CAU_Oori_1.0 have been submitted to the NCBI BioProject database under the accession number PRJNA529571 [307]. Detailed information of the SV data identified in this study is available in the Figshare repository (10.6084/m9.figshare.21993191.v9) [308]. The custom workflow and scripts are available in the GitHub repository (https://github.com/atongsa/convergency_sv) [309] and Zenodo (10.5281/zenodo.11227793) [310] under the MIT license.
