# Supplementary material for: Structural variant landscapes reveal convergent signatures of evolution in sheep and goats
Source: Genome Biol. 2024 Jun 6;25:148. doi: 10.1186/s13059-024-03288-6 (PMC11155191; doi:10.1186/s13059-024-03288-6)
Supplement: Supplementary file 1 — Additional file 1: Supplementary Results and Supplementary Figures 1–30. Fig. S1. Genomic characterization and synteny landscape for the de novo assembled Asiatic mouflon genome. Fig. S2. Venn diagrams. Fig. S3. The size distribution of SVs exceeding 1 kb by variant types in sheep and goats. Fig. S4. Experimental validation of the SVs identified in this study. Fig. S5. Genomic features of the sheep and goat genomes. Fig. S6. Distribution of short length SVs and transposable elements. Fig. S7. The pattern of linkage disequilibrium (LD) decay in genomes of sheep and goats based on SNPs. Fig. S8. Principal component analysis (PCA) of domestic sheep and domestic goats based on structural variants. Fig. S9. The cross-validation errors for different values of K in admixture analysis of sheep and goats. Fig. S10. Genome-wide selective test for candidate genes associated with the domestication of domestic sheep and domestic goats. Fig. S11. Genome-wide selective test for candidate genes associated with the domestication of domestic goats during two different developmental stages. Fig. S12. Genome-wide selective test for candidate genes associated with the genetic improvement of domestic sheep and domestic goats. Fig. S13. Functional enrichment analysis reveals significant GO terms and KEGG pathways for the candidate selected genes associated with the fertility. Fig. S14. Genome-wide selective test for candidate genes associated with the reproduction traits of domestic sheep and domestic goats based on SNP data. Fig. S15. Genome-wide selective test for candidate genes associated with the wool and cashmere related trait of domestic sheep and domestic goats. Fig. S16. Genome-wide selective test for candidate genes associated with the dairy trait of domestic sheep and domestic goats. Fig. S17. Genome-wide selective test for candidate genes associated with the meat production trait of domestic sheep and domestic goats. Fig. S18. Evolution and function analysis of the deletions i [file 13059_2024_3288_MOESM1_ESM.docx]

**Supplementary Information**

**Additional file 1**

**Supplementary Results**

***De novo* assembly and annotation of Asiatic mouflon genome**

The whole genome of a female Asiatic mouflon was *de novo* assembled, with a heterozygosity of 0.3% and a size of 2.49 Gb, as preliminarily determined by k-mer spectrum analysis. The sequencing data comprised of ~80× PacBio long reads, ~100× Hi-C reads, ~100× BioNano data and ~100× Illumina paired-end reads. An initial PacBio-based contig assembly was generated, with 343 primary contigs, an N50 size of 47.03 Mb, and a total length of 2.64 Gb. We obtained 193 scaffolds with an N50 length of 107.10 Mb after sorting the contigs using the BioNano data. We then anchored the scaffolds on pseudochromosomes using the Hi-C data, and obtained the Asiatic mouflon chromosome-level genome assembly *Amuf_v1* (NCBI accession GCA_014523465.1).

In the *Amuf_v1* genome, the 18,790 annotated genes showed an average length of 47,085 bp and an average exon number of 9.61 per gene, and 91.70% of the annotated genes were supported by RNA-Seq. Non-coding RNA annotation predicted 251,892 tRNA, 375 rRNA, 654 snRNA, and 769 microRNA genes. Additionally, repeat annotation identified 1.22 Gb of repetitive sequences (46.04%) overall, among which transposable elements (TEs) accounted for 98.65% of all the repetitive sequences. Among the TEs, class I elements (42.02%, including 31.99% long interspersed elements (LINEs), 6.59% long terminal repeats (LTRs) and 3.44% short interspersed elements (SINEs)) were abundant, while class II elements (DNA, 2.71%, miniature inverted repeat transposable elements (MITEs), 0.61% and rolling circle (RC) transposons, 0.03%) represented only 3.36% of the whole genome. Also, we annotated 174 superfamilies.

**SV discovery and characterization**

At the species level, most of the wild sheep (6 out of 7 species) and wild goats (5 out of 6 species) harboured a greater number of SVs than their domestic counterparts (Fig. 2B). At the population level, we did not observe obvious differences in the SV numbers among populations of either domestic sheep or domestic goats (Additional file 2: Table S6).

Regarding the distribution of SV length, 30.62% and 27.82% of SVs were 50 – 100 bp, 27.80% and 26.71% were 100 – 250 bp, 23.49% and 28.91% were 250 bp – 1 kb, and only 18.09% and 16.56% were > 1 kb in sheep and goats, respectively (Additional file 2: Table S7).

**SV-genes selected for important agronomic traits**

We identified 272 and 205 SV-genes for the trait of wool/hair fineness in sheep and goats, respectively (Additional file 1: Fig. S15, Additional file 2: Table S27). Of the two sets of selected genes, 16 showed convergent selection in sheep and goats (e.g., *COL21A1*, *TMEM117*, *NKAIN2*, *DGKB* and *MACROD2*; Additional file 2: Table S22), and their critical functions are related to the maintenance of the extracellular matrix integrity (e.g., *COL21A1*) [1]. For the dairy traits, we identified 241 and 230 SV-genes in sheep and goats, respectively (Additional file 1: Fig. S16, Additional file 2: Table S27), of which 11 genes (e.g., *TRNAC-GCA*, *PLEKHA5*, *KLHL1*, *RALYL* and *ZNF804B*; Additional file 2: Table S22) underwent shared signals of selection between the two ruminants. The 11 SV-genes under convergent selection play important roles in milk fat percentage (*PLEKHA5*) [2] and protein yield (*RALYL*) [3]. Also, we detected 287 and 261 SV-genes for the meat traits in sheep and goat respectively and 15 SV-genes (e.g., *LRP1B*, *PHLPP1* and *THSD7A*) that have undergone convergent selection during the genetic improvement of meat traits (Additional file 1: Fig. S17, Additional file 2: Tables S22 and S27). Annotation of the 15 genes indicated their essential roles in fat deposition and content (*LRP1B*) [4], average daily gain (*PHLPP1*) [5] and body weight (*THSD7A*) [6].

**Molecular analysis of the deletions in *BMPR2***

We calculated nucleotide diversity in *BMPR2* and its upstream and downstream regions, and observed a reduction in nucleotide diversity in in native goat relative to bezoar, but an increase of nucleotide diversity in the Middle Eastern sheep populations relative to Asiatic mouflon (Additional file 1: Figs. S17A and S18A). We examined the allele frequency of SV_w_15555 and found an obvious difference in the SV frequencies between Asiatic mouflon (0.091) and the Middle Eastern sheep populations (0.754). In goats, the frequency of DEL00018513 in bezoar is 0.118, while it is absent in the native goat populations.

Phylogenic analysis showed that the SV_w_15555 sequence in *BMPR2* of sheep was also observed in the Bovinae, Caprinae and Hippotraginae genomes. The presence of SV_w_155555 in Bovidae suggests its earliest origin in the last common ancestor ~16 million years ago (Additional file 1: Fig. S22B). We predicted two kinds of (GCCARATG and CACAGS) in the SV_w_15555 sequence using MEME software, which are functionally related to immune effector and excretion (Additional file 1: Fig. S22C). RNA-seq analysis indicated that the expression of *BMPR2* in ovary and corpus luteum was higher than the other tissues (Fig. 7A). These results suggest that SV_w_15555 could introduce transcription factor binding motifs that decrease the expression of *BMPR2* in the reproductive tissues of sheep, which was possibly related to the higher reproduction capability of domestic sheep compared to Asiatic mouflon. Similarly, the DEL00018513 sequence in *BMPR2* of goat occurs in the Bovinae, Caprinae, Hippotraginae, Odocoileinae and Cervinae genomes. The presence of DEL00018513 in both Cervidae and Bovidae suggests its origin in the last common ancestor ~23 million years ago (Additional file 1: Fig. S23B). Two kinds of transcription factor binding motifs (GCYTGS and AGATWG) predicted in the DEL00018513 were associated with blood pressure, olfactory receptor and immune response (Additional file 1: Fig. S23C). Transcriptome data showed that the expression of *BMPR2* in ovarian follicle was higher than in muscle and skin (Fig. 7A). These results imply that transcription factor binding motifs in DEL00018513 could increase the expression of *BMPR2* in the reproductive tissues of goats, which was probably associated with the higher reproduction capability of domestic goats compared to bezoar.

**Supplementary References**

1. Goyer B, Thériault M, Gendron SP, Brunette I, Rochette PJ, Proulx S. Extracellular matrix and integrin expression profiles in fuchs endothelial corneal dystrophy cells and tissue model. Tissue Eng Part A. 2018;24:607–15.
2. Wang P, Li X, Zhu YH, Wei JN, Zhang CX, Kong QF, et al. Genome-wide association analysis of milk production, somatic cell score, and body conformation traits in Holstein cows. Front Genet. 2022;9:932034.
3. Abdel-Shafy H, Awad MAA, El-Regalaty H, El-Assal SE-D, Abou-Bakr S. Prospecting genomic regions associated with milk production traits in Egyptian buffalo. J Dairy Res. 2020;87:389–96.
4. Zhang L, Wang F, Gao G, Yan X, Liu H, Liu Z, et al. Genome-wide association study of body weight traits in Inner Mongolia cashmere goats. Front Vet Sci. 2021;8:752746.
5. Zhou S, Ding R, Meng F, Wang X, Zhuang Z, Quan J, et al. A meta-analysis of genome-wide association studies for average daily gain and lean meat percentage in two Duroc pig populations. BMC Genomics. 2021;22:12.
6. Ben-Jemaa S, Senczuk G, Ciani E, Ciampolini R, Catillo G, Boussaha M, et al. Genome-wide analysis reveals selection signatures involved in meat traits and local adaptation in semi-feral Maremmana cattle. Front Genet. 2021;12:675569.

**Supplementary Figures**

**
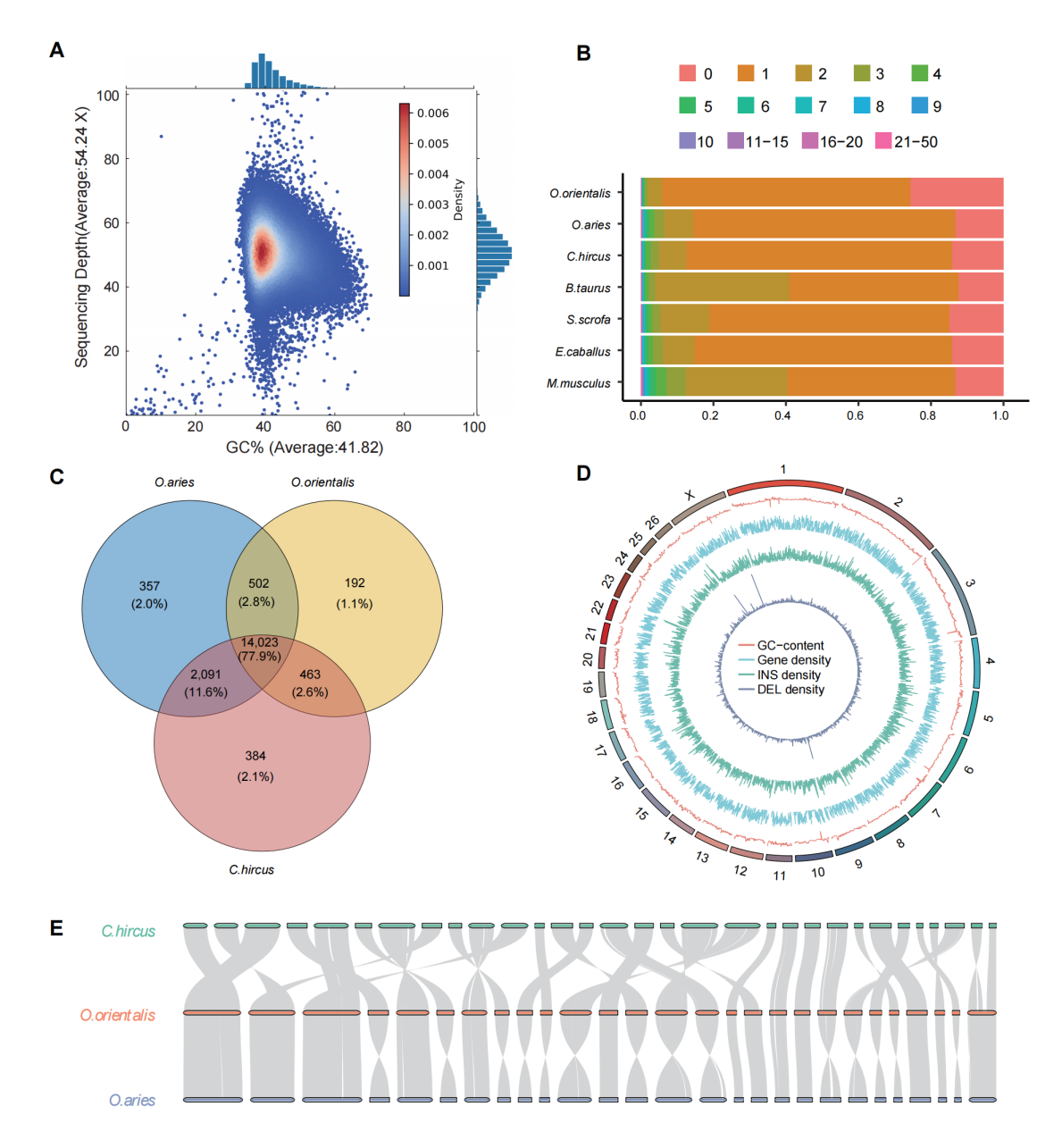
**

**Fig. S1. Genomic characterization and synteny landscape for the *de novo* assembled Asiatic mouflon genome. A** Distribution of Sequencing depth and GC content for the genome assembly using PacBio subreads. **B** Proportions of different gene numbers in 18,777 gene families in the seven mammal species. **C** Venn diagram shows the number of homologs among Asiatic mouflon (*O. orientalis*), domestic sheep (*O. aries*) and domestic goat (*C. hircus*). **D** Chromosomal features of the assembled Asiatic mouflon genome. From outside to inside, the red lines indicate GC content, the blue lines indicate gene density, the green lines represent insertion (INS) density and the purple lines represent deletion (DEL) density. **E** Syntenic plots between the assembled genomes of Asiatic mouflon and domestic sheep and goats.

**
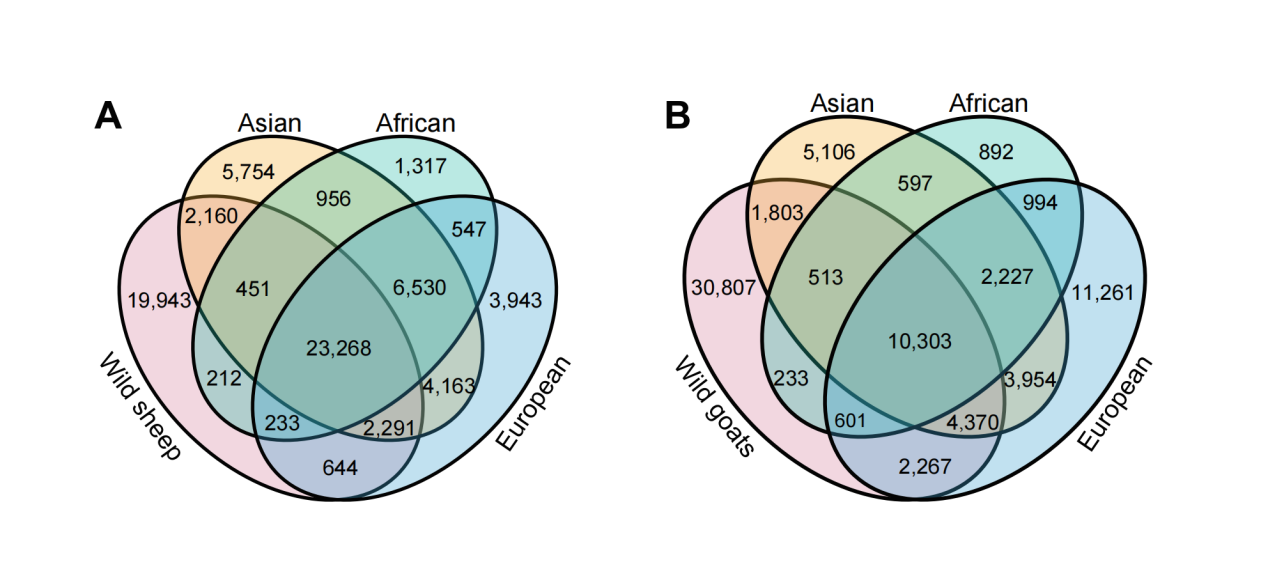
**

**Fig. S2. Venn diagrams. A** The Venn diagrams of SV quantities among wild sheep species and Asian, African, and European populations of domestic sheep. **B** The Venn diagrams of SV quantities among wild goat species and Asian, African, and European populations of domestic goats.

**
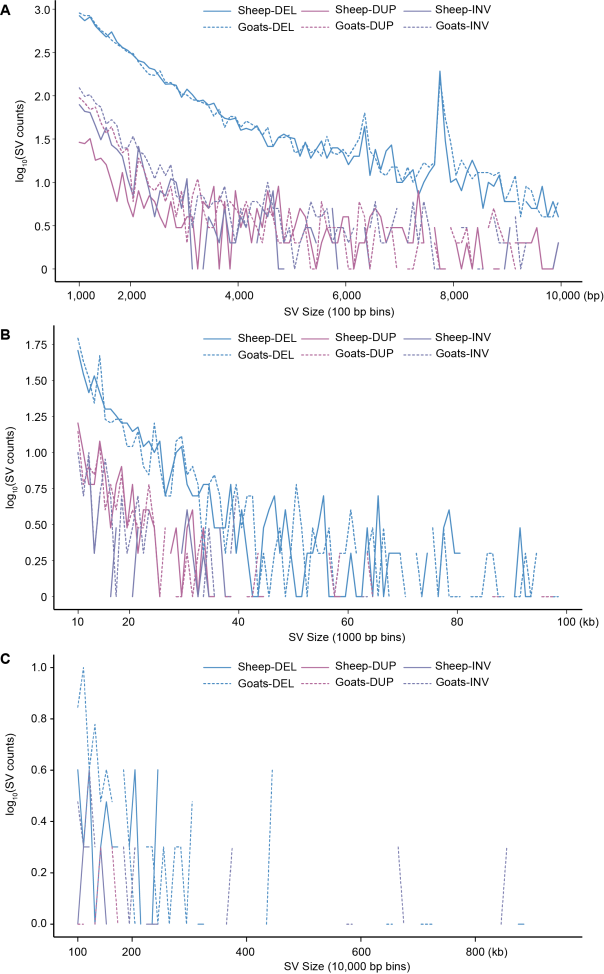
**

**Fig. S3. The size distribution of SVs.** The size distribution of SVs exceeding 1 kb by variant types in sheep and goats.


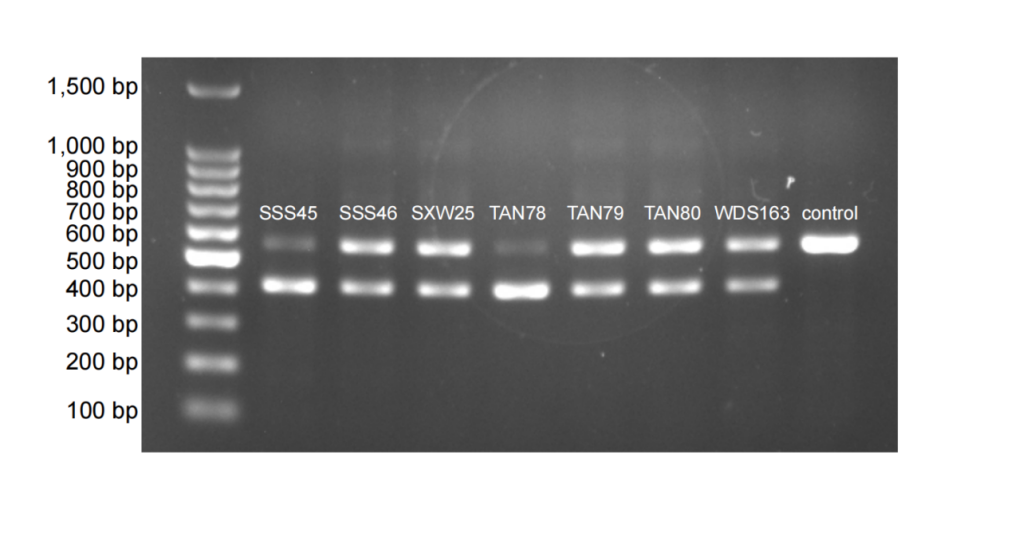


**Fig. S4. Experimental validation of the SVs identified in this study.** Experiment results of SV genotypes of control sample (wild type: MSF174) and test samples (mutant types: SSS45, SSS46, SXW25, TAN78, TAN79, TAN80, WDS163) are shown in the electrophoretic gel.

**
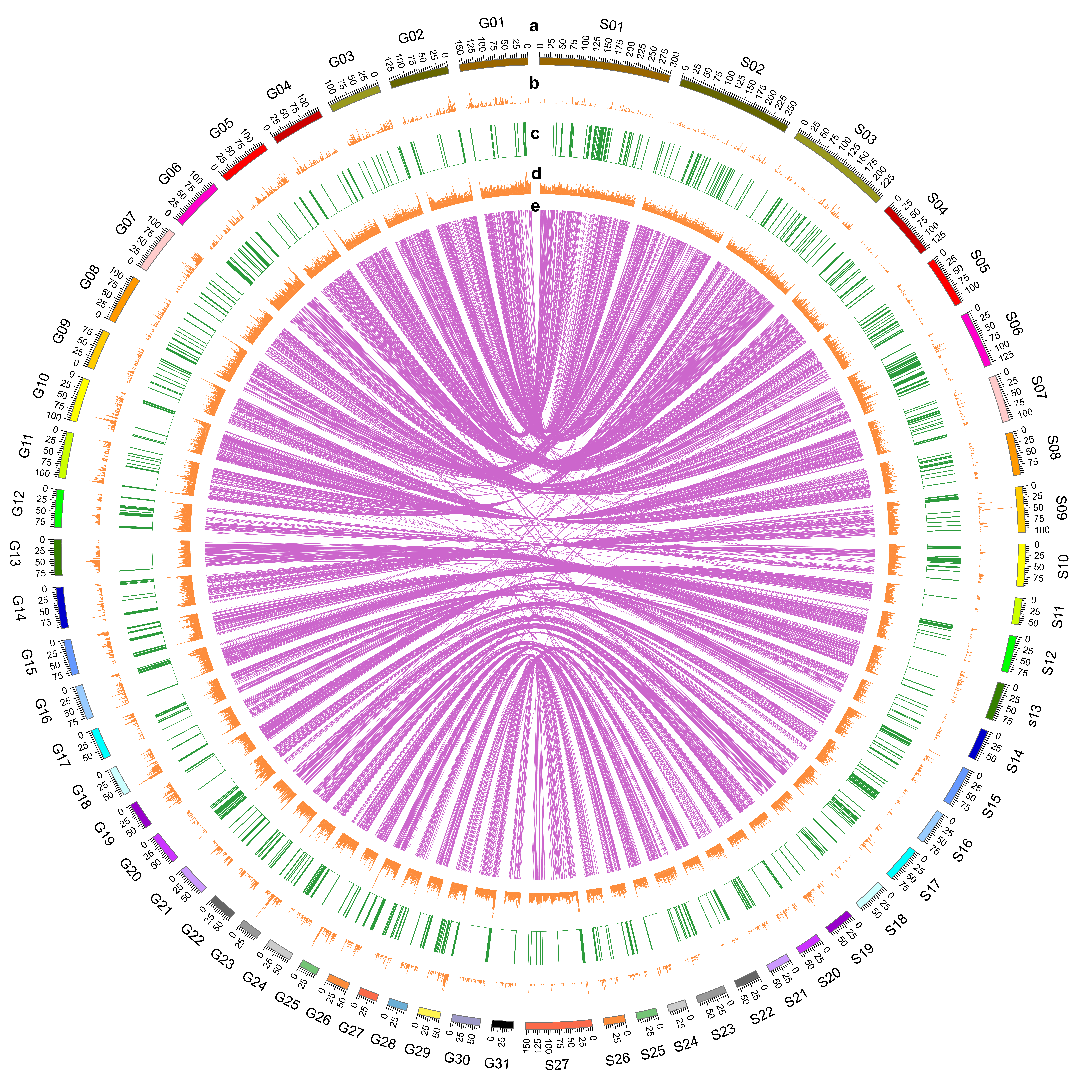
**

**Fig. S5. Genomic features of the sheep and goat genomes.** The ideograms represent the sheep and goat chromosomes in Mb scale (**a**), number of SVs per Mb (*n* = 0 – 104) (**b**), SV hotspot regions (**c**), number of annotated genes per Mb (*n* = 0 – 43) (**d**), and syntenic genes between the sheep and goat genomes (**e**).

**
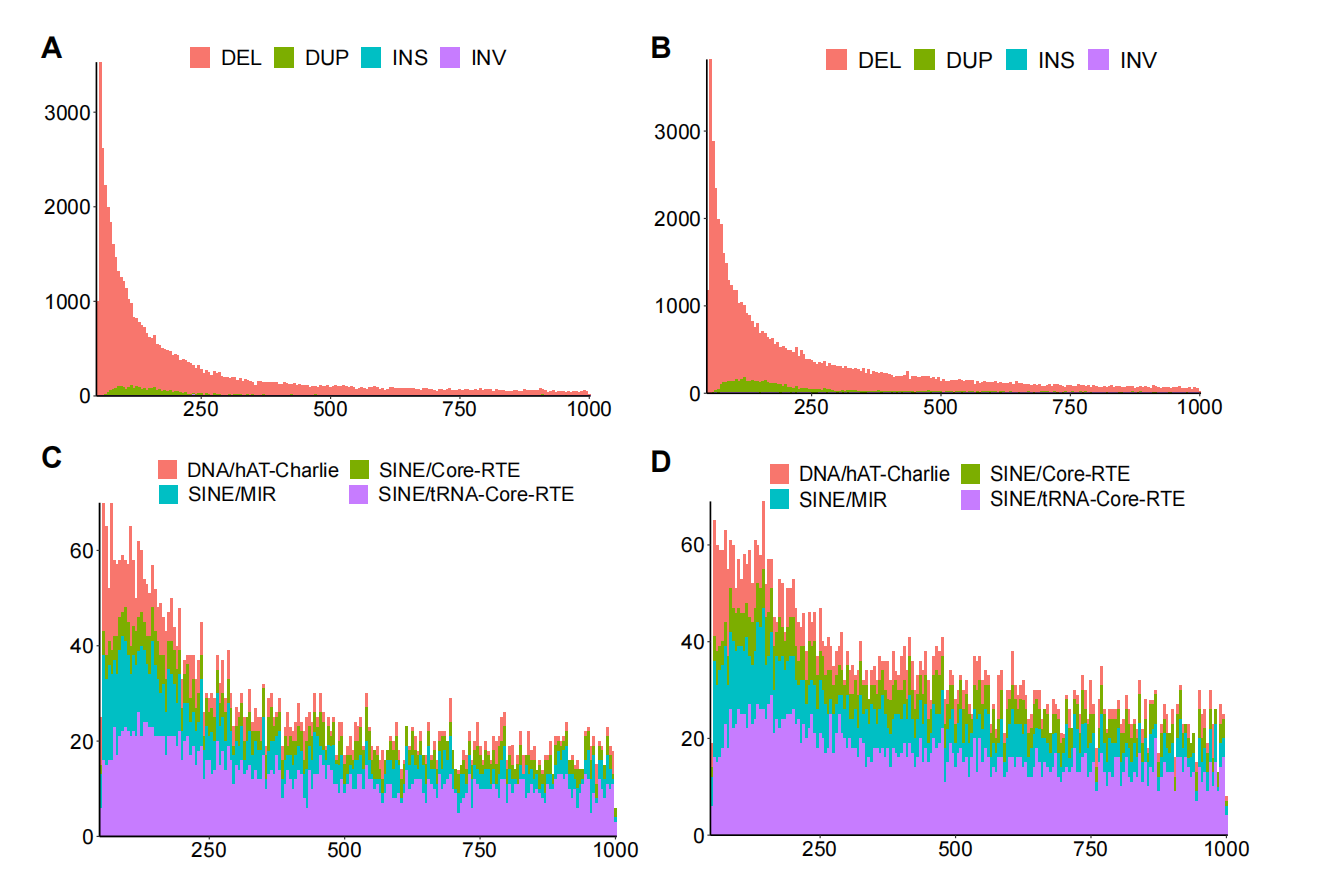
**

**Fig. S6. Distribution of short length SVs and transposable elements. A** Distribution of the whole SVs with a length of 1 – 1,000 bp in domestic sheep genome. **B** Distribution of the whole SVs with a length of 1 – 1,000 bp in domestic goat genome. **C** Distribution of the TEs with a length of 1 – 1,000 bp in domestic sheep genome. **D** Distribution of the TEs with a length of 1 – 1,000 bp in domestic goat genome.

**
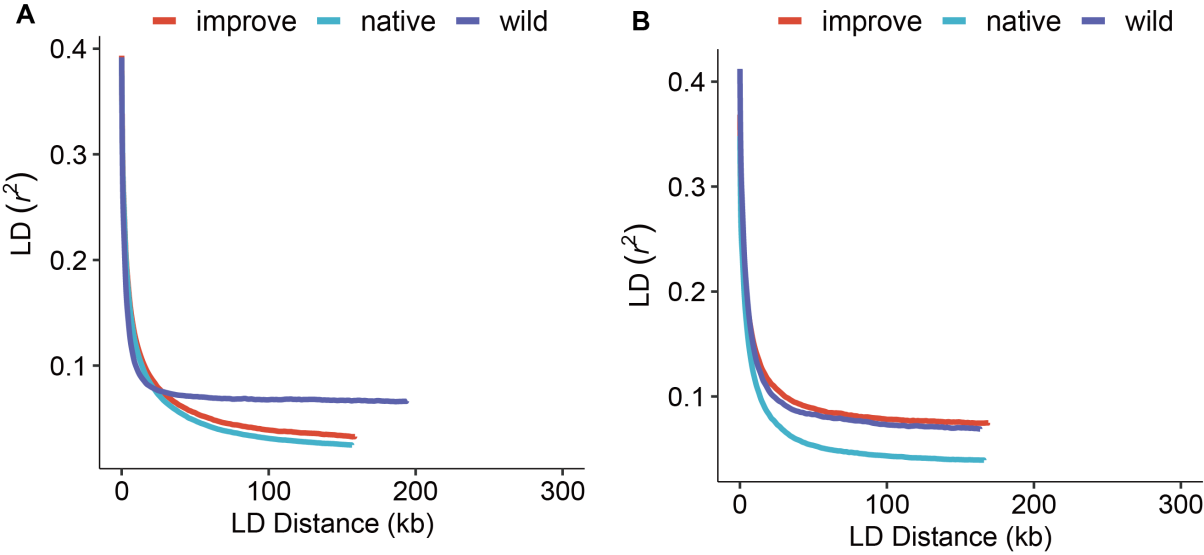
**

**Fig. S7. The pattern of linkage disequilibrium (LD) decay in genomes of sheep and goats based on SNPs. A** The pattern of LD decay in the genomes of Asiatic mouflon, native sheep and improved sheep. **B** The pattern of LD decay in the genomes of bezoar, native goat and improved goat.

**
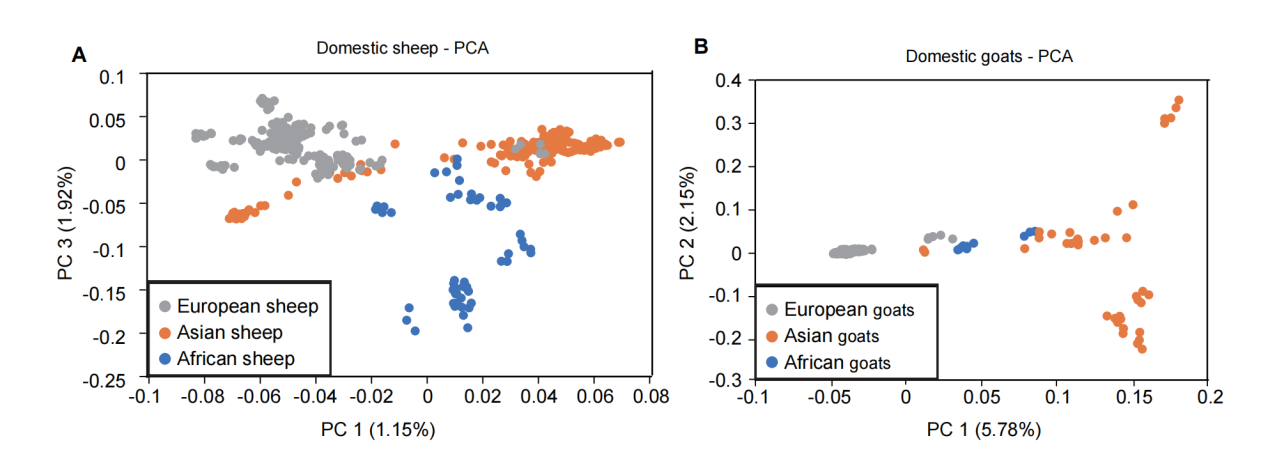
**

**Fig. S8. Principal component analysis (PCA) of domestic sheep** (**A**) **and domestic goats** (**B**) **based on the structural variants.**

**
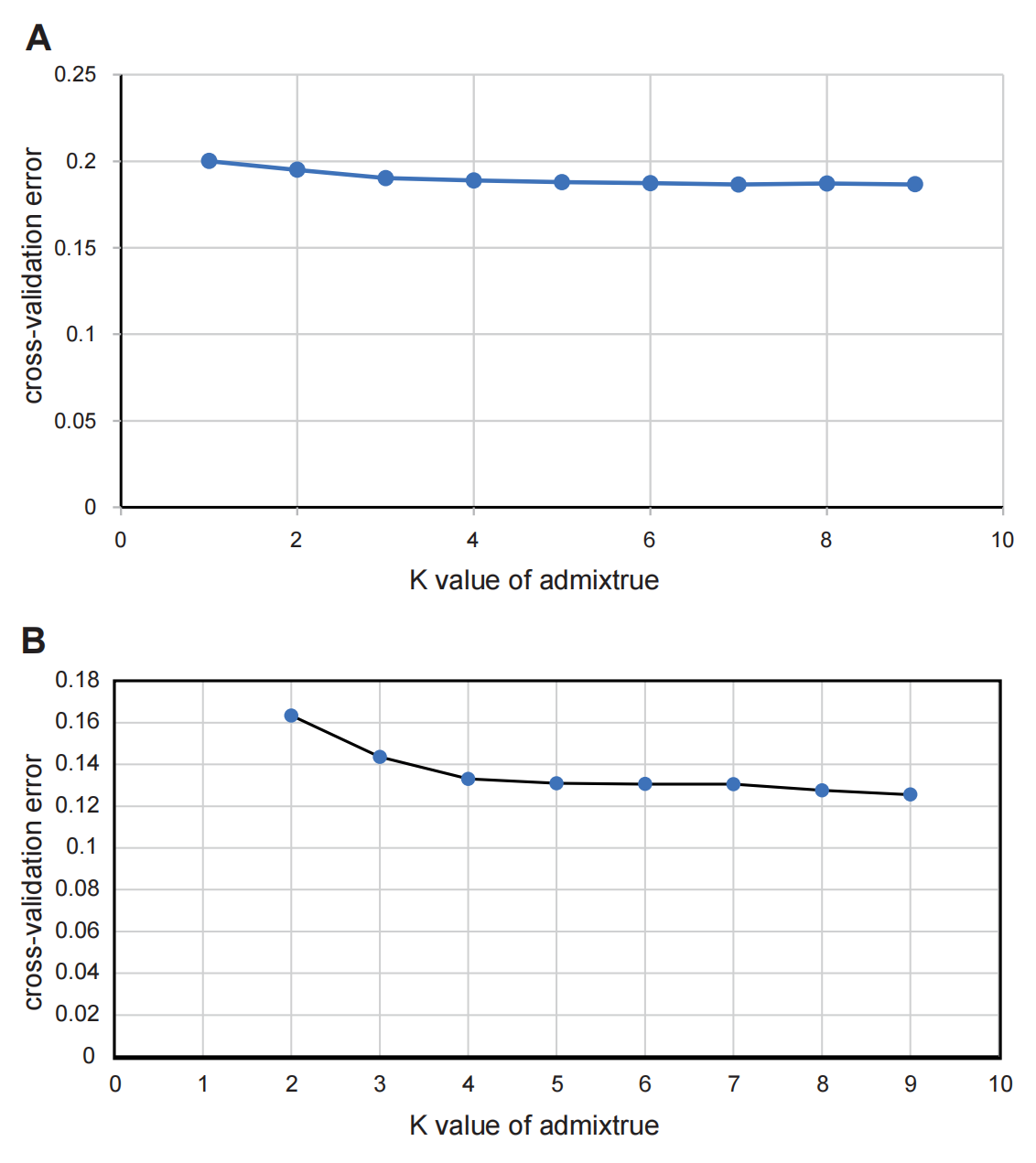
**

**Fig. S9. The cross-validation errors for different values of *K* in admixture analysis of sheep (A) and goats (B).**

**
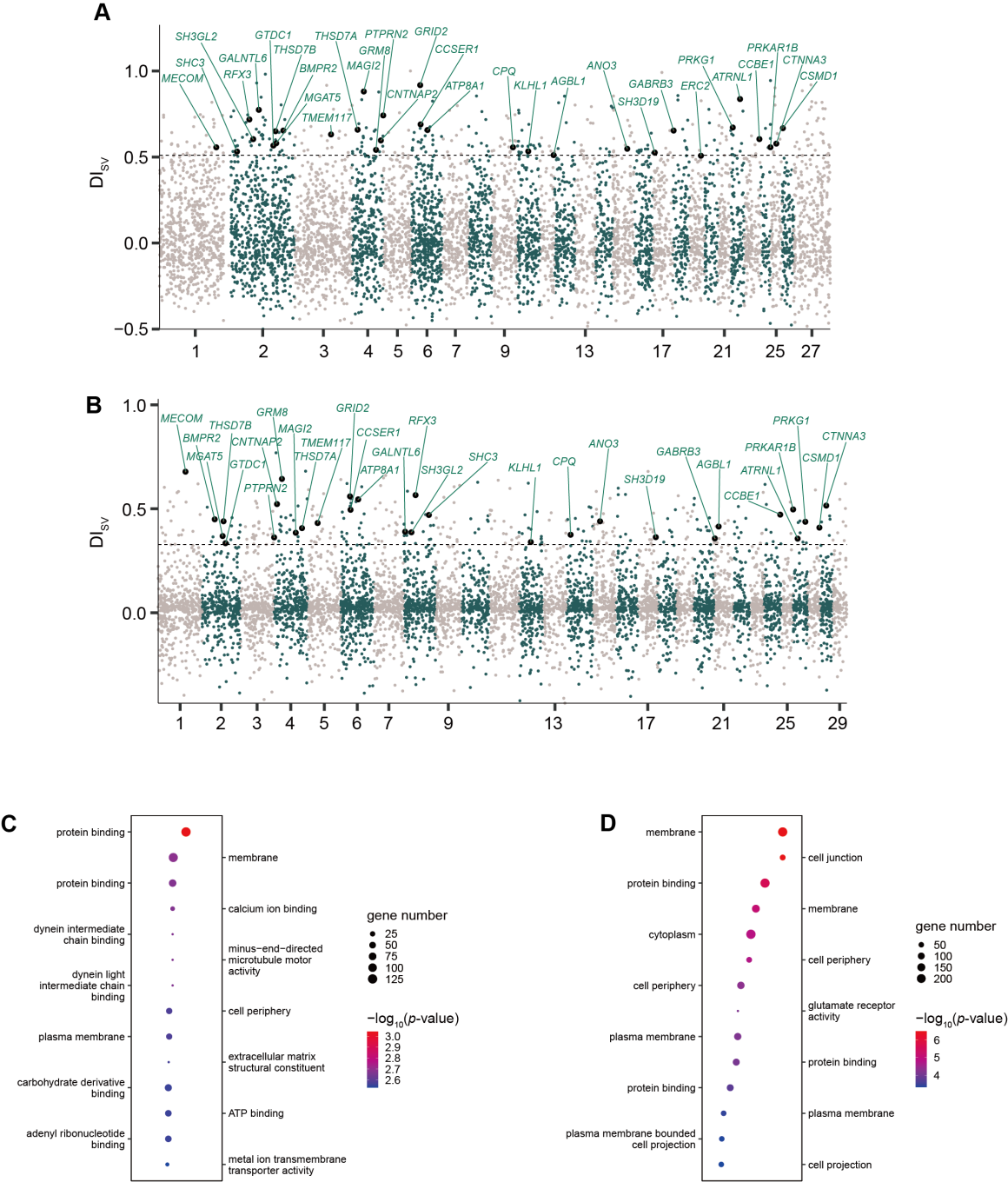
**

**Fig. S10. Genome-wide selective test for candidate genes associated with the domestication of domestic sheep and domestic goats. A** Genome-wide DI_SV_ values between Asiatic mouflon and the Middle Eastern sheep populations. **B** Genome-wide DI_SV_ values between bezoar and native goat populations. **C** Top enriched GO terms and KEGG pathways for candidate selected genes associated with sheep domestication. **D** Top enriched GO terms and KEGG pathways for candidate selected genes associated with goat domestication. In figures **A** and **B**, the horizontal dotted line represents the threshold of top 5% DI_SV_ value. The genes convergently selected in sheep and goats are shown in the figure, and those reported previously to be associated with animal domestication are represented in green font.


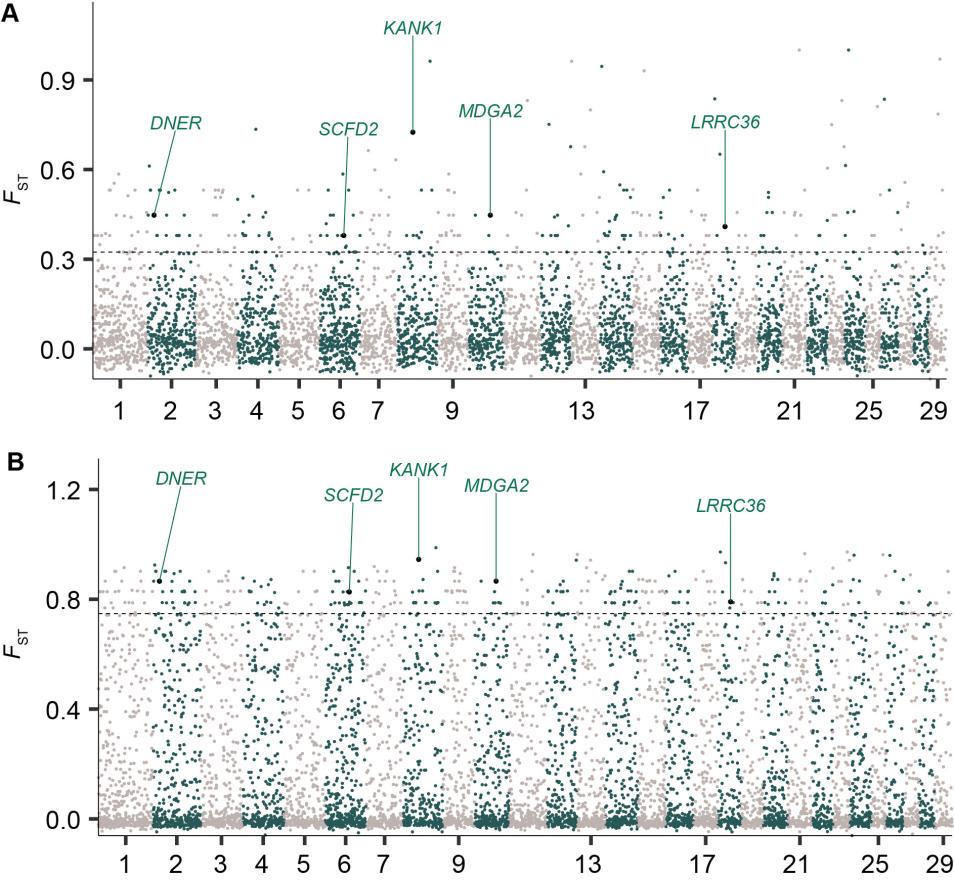


**Fig. S11. Genome-wide selective test for candidate genes associated with the domestication of domestic goats during two different developmental stages.** **A** Genome-wide *F*_ST_ values between the genomes of bezoar and ancient goat remains. **B** Genome-wide *F*_ST_ values between the genomes of ancient goat and native goat populations. The horizontal dotted line represents the threshold of top 5% *F*_ST_ value. The domestication-related genes commonly selected during the two developmental stages of goats are shown in green.


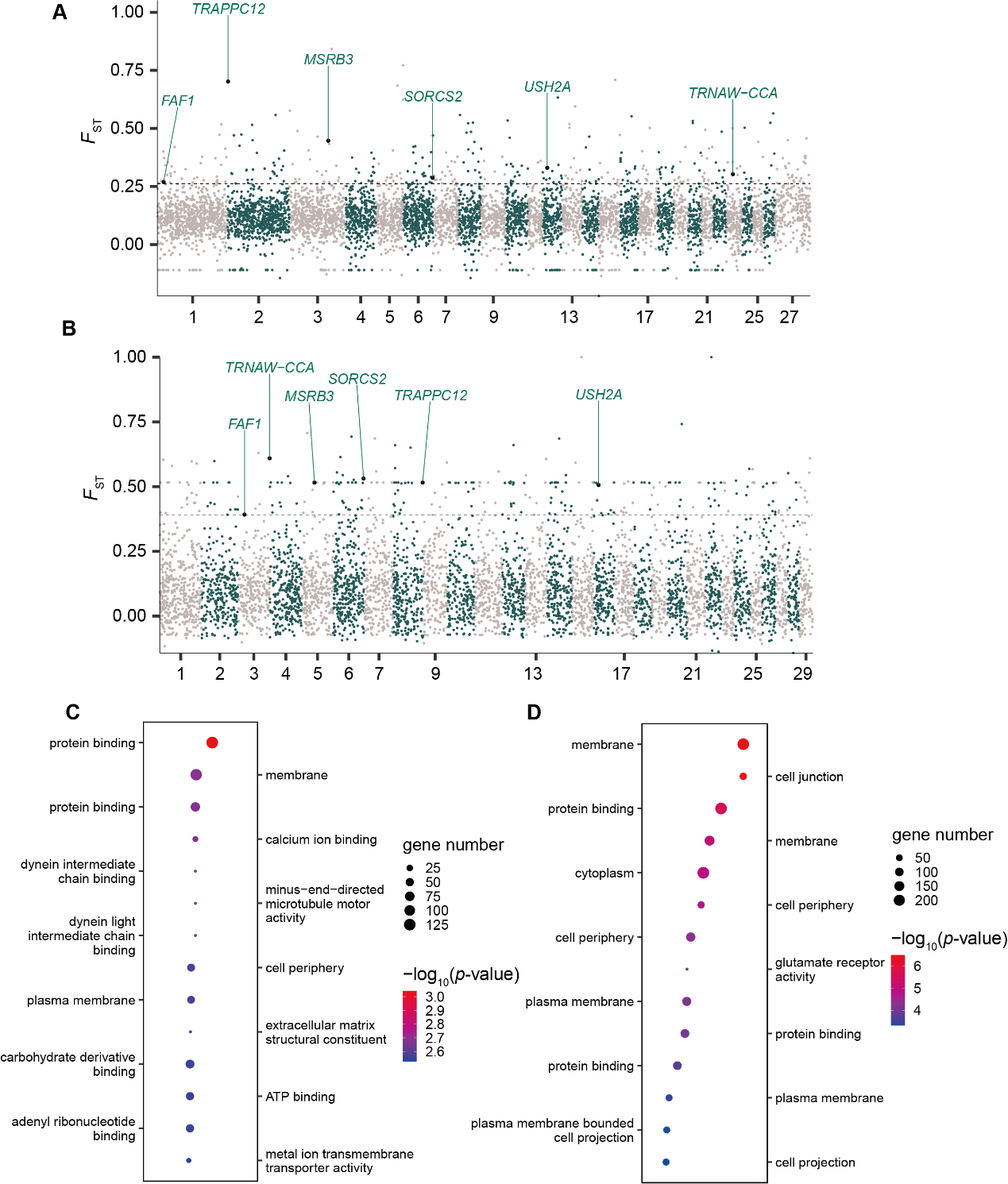


**Fig. S12. Genome-wide selective test for candidate genes associated with the genetic improvement of domestic sheep and domestic goats. A** Genome-wide *F*_ST_ values across all domestic sheep populations. **B** Genome-wide *F*_ST_ values across all domestic goat populations. **C** Top enriched GO terms and KEGG pathways for candidate selected genes associated with sheep improvement. **D** Top enriched GO terms and KEGG pathways for candidate selected genes associated with goat improvement. In figures **A** and **B**, the horizontal dotted line represents the threshold of top 5% *F*_ST_ value. The genes convergently selected in sheep and goats are shown in the figure, and those reported previously to be associated with animal improvement are represented in green font.


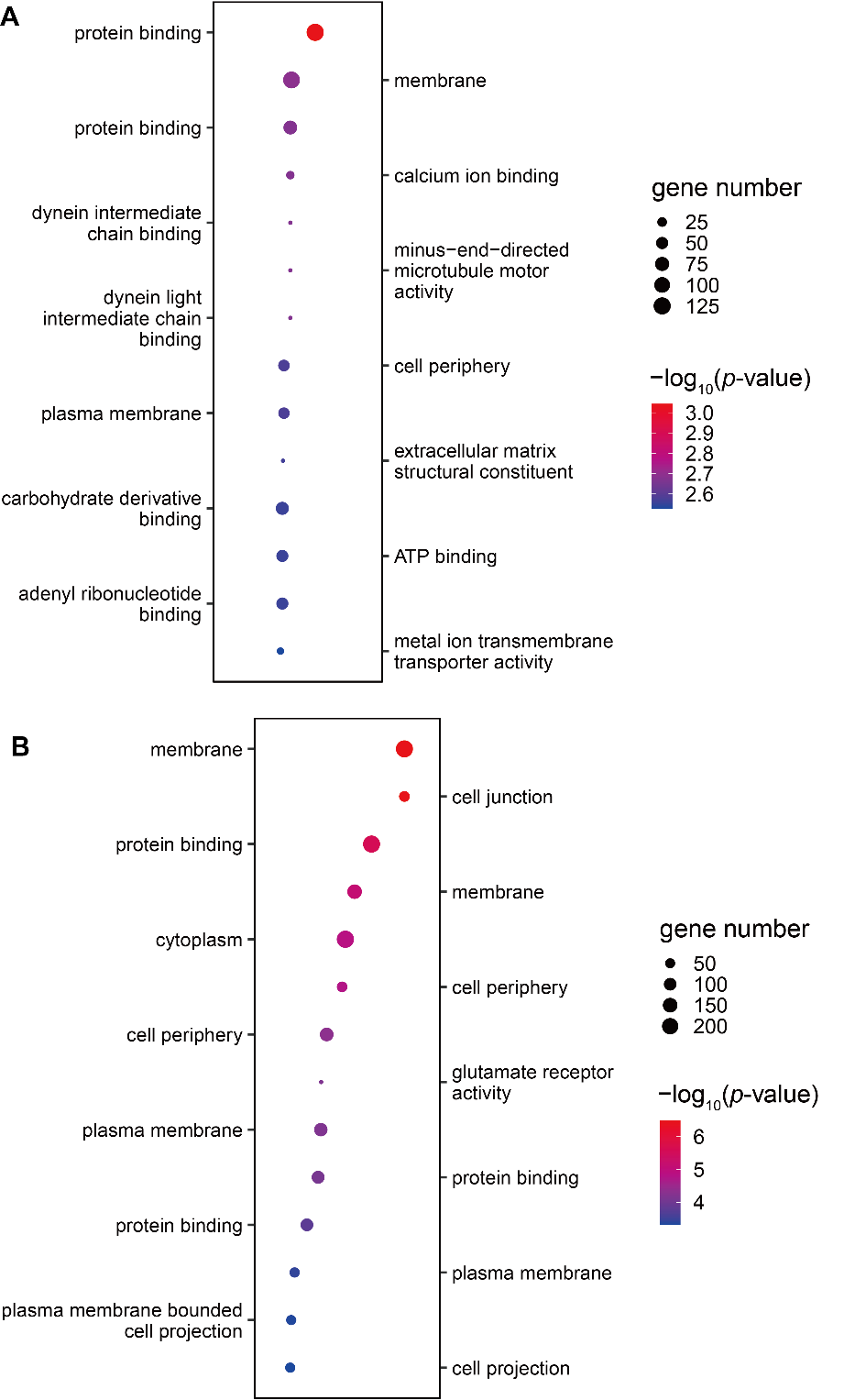


**Fig. S13. Functional enrichment analysis reveals significant GO terms and KEGG pathways for the candidate selected genes associated with the fertility in domestic sheep (A) and domestic goats (B).**

**
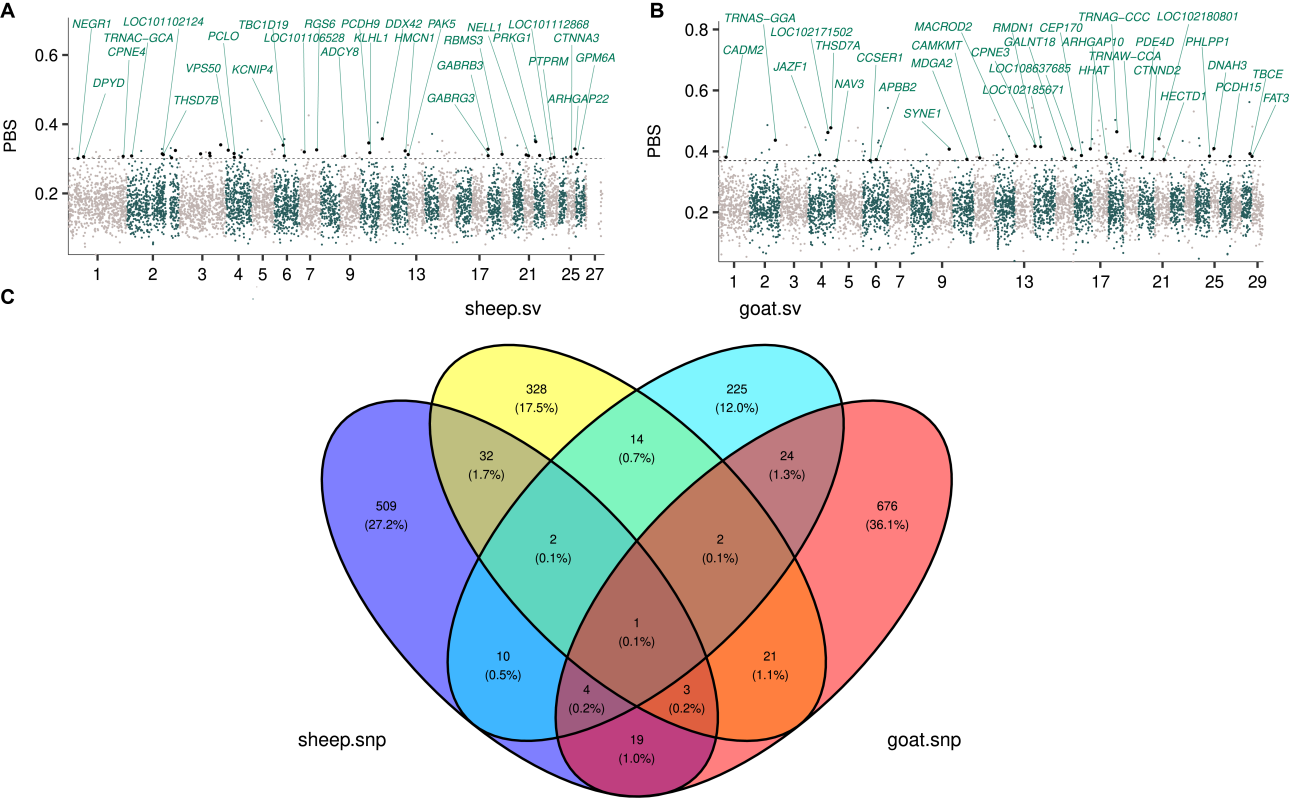
**

**Fig. S14. Genome-wide selective test for candidate genes associated with the reproduction traits of domestic sheep and domestic goats based on SNP data. A** Genome-wide PBS values for reproduction traits between prolific sheep (WDS, HUS, SXW, FIN, GOT) and non-prolific sheep (TAN, SSS, ALS, BSB, OU, SFK) populations. **B** Genome-wide PBS values for reproduction traits between prolific goat (SAN, BOE, MAT) and non-prolific goat (BOT, LEI, MAU, SON, DIA, SOF, AND, MEN) populations. **C** The Venn diagrams of the quantities of candidate selected genes identified using the sheep SNP, sheep SV, goat SV and goat SNP data. In figures **A** and **B**, the horizontal dotted line represents the threshold of the top 1% of PBS values for each selective test. The genes commonly selected in the SNP and SV analysis are shown in the panels, and those reported previously to be associated with reproduction traits are represented in green font. For detailed information of the populations involved in the selective tests, please see Additional file 2: Table S1.


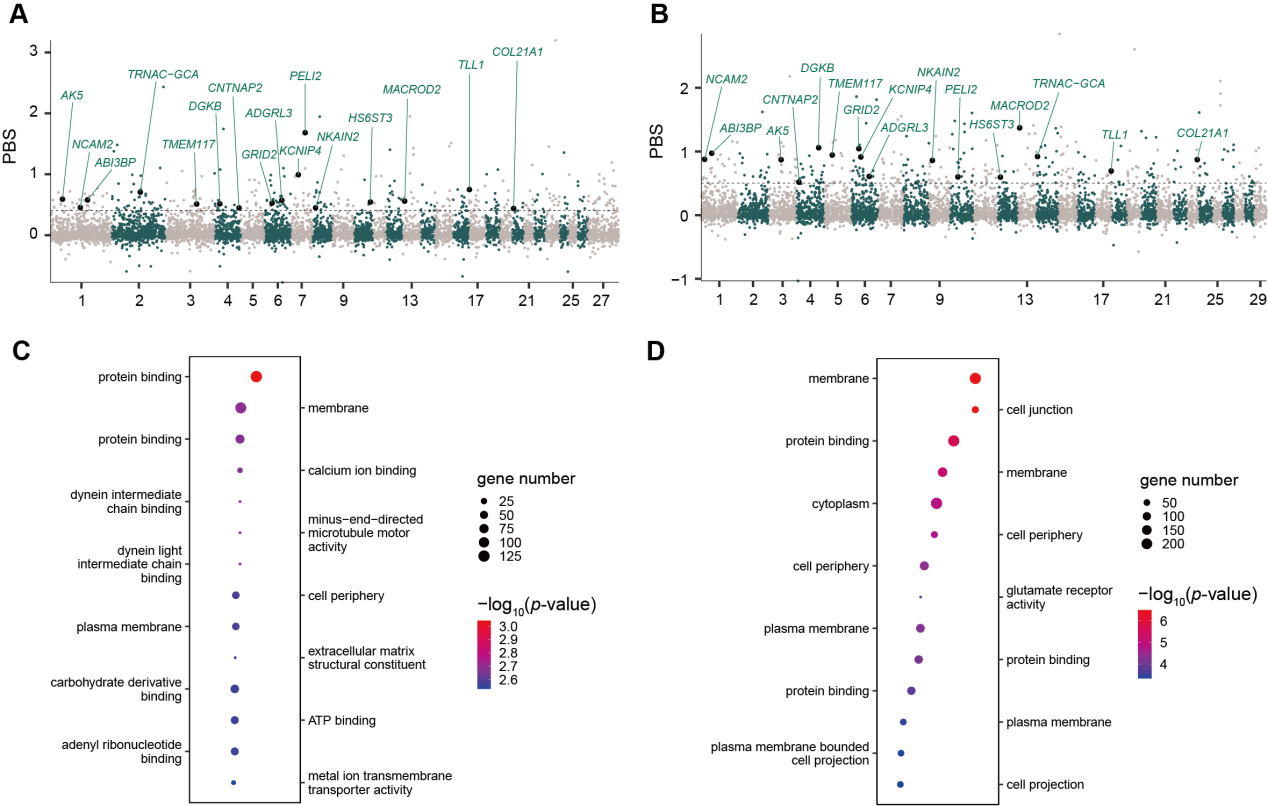


**Fig. S15. Genome-wide selective test for candidate genes associated with the wool and cashmere related trait of domestic sheep and domestic goats. A** Genome-wide PBS values between fine-wool sheep and coarse-wool sheep populations. **B** Genome-wide PBS values between angora goat and coarse-wool goat populations. **C** Top enriched GO terms and KEGG pathways for candidate selected genes associated with wool related trait of sheep. **D** Top enriched GO terms and KEGG pathways for candidate selected genes associated with mohair related trait of goats. In figures **A** and **B**, the horizontal dotted line represents the threshold of top 5% PBS value. The genes convergently selected in sheep and goats are shown in the figure.


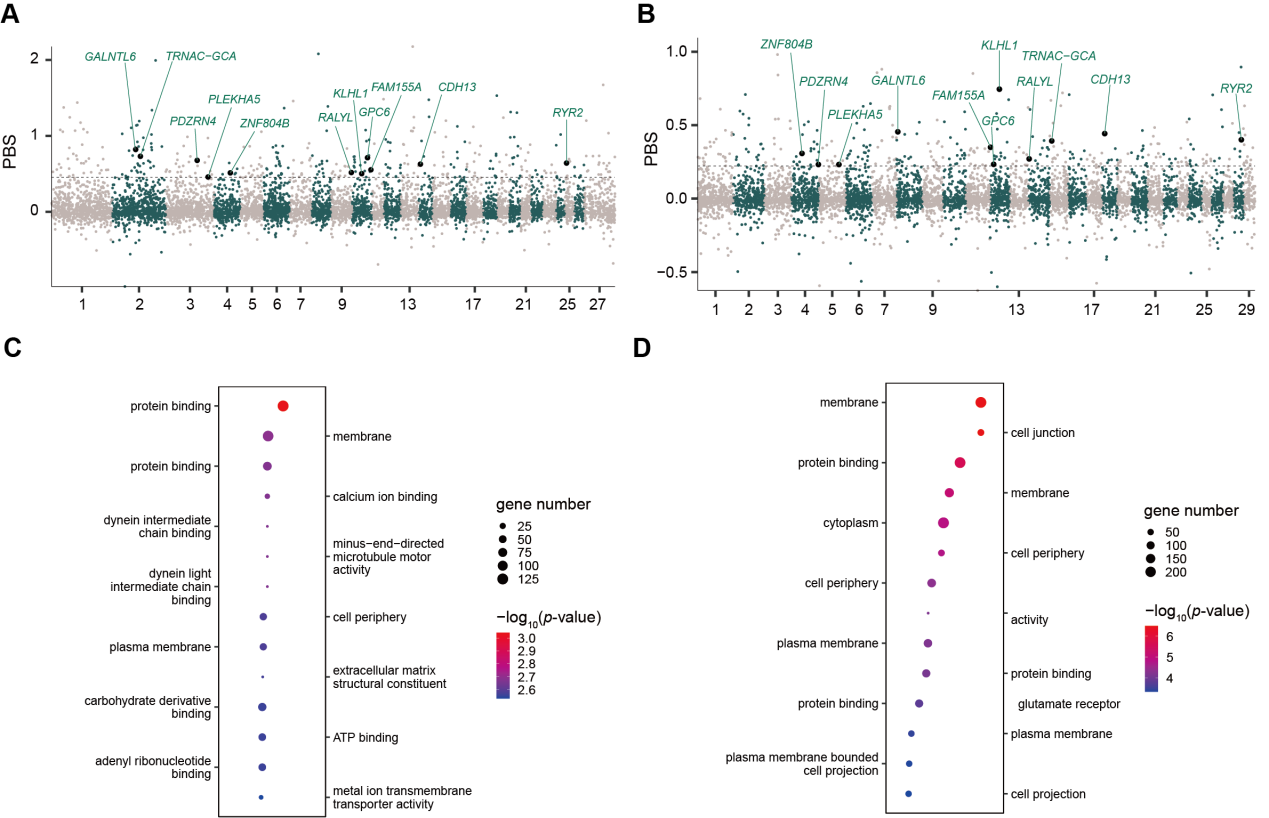


**Fig. S16. Genome-wide selective test for candidate genes associated with the dairy trait of domestic sheep and domestic goats. A** Genome-wide PBS values between dairy sheep and low milk yielding sheep populations. **B** Genome-wide PBS values between dairy goat and low milk yielding goat populations. **C** Top enriched GO terms and KEGG pathways for candidate selected genes associated with milk related trait of sheep. **D** Top enriched GO terms and KEGG pathways for candidate selected genes associated with milk related trait of goats. In figures **A** and **B**, the horizontal dotted line represents the threshold of top 5% PBS value. The genes convergently selected in sheep and goats are shown in the figure.


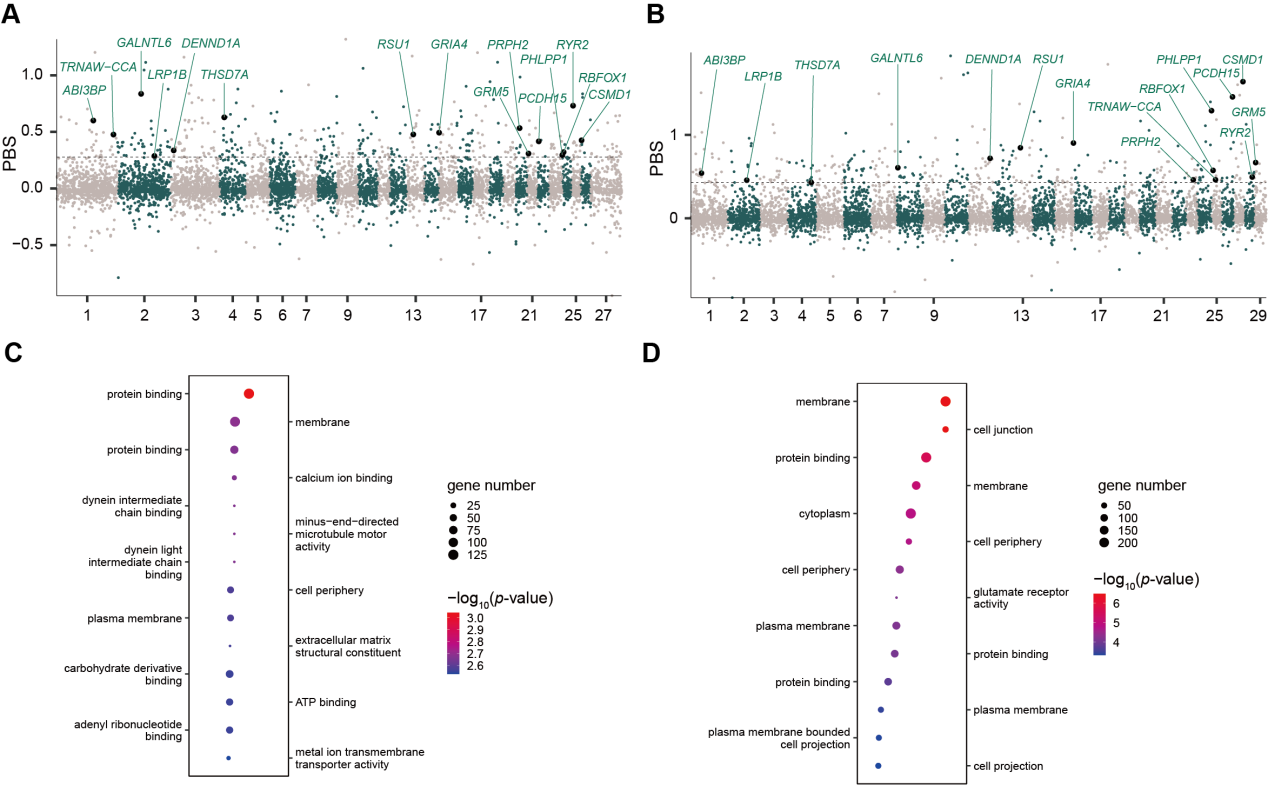


**Fig. S17. Genome-wide selective test for candidate genes associated with the meat production trait of domestic sheep and domestic goats. A** Genome-wide PBS values between the sheep populations specialized for meat (meat populations) and for other products (non-meat populations) or dual purposes. **B** Genome-wide PBS values between the goat populations for meat (meat populations) and for other products (non-meat populations) or dual purposes. **C** Top enriched GO terms and KEGG pathways for candidate selected genes associated with meat related trait of sheep. **D** Top enriched GO terms and KEGG pathways for candidate selected genes associated with meat related trait of goats. In figures **A** and **B**, the horizontal dotted line represents the threshold of top 5% PBS value. The genes convergently selected in sheep and goats are shown in the figure, and those reported previously to be associated with meat related traits are represented in green font.


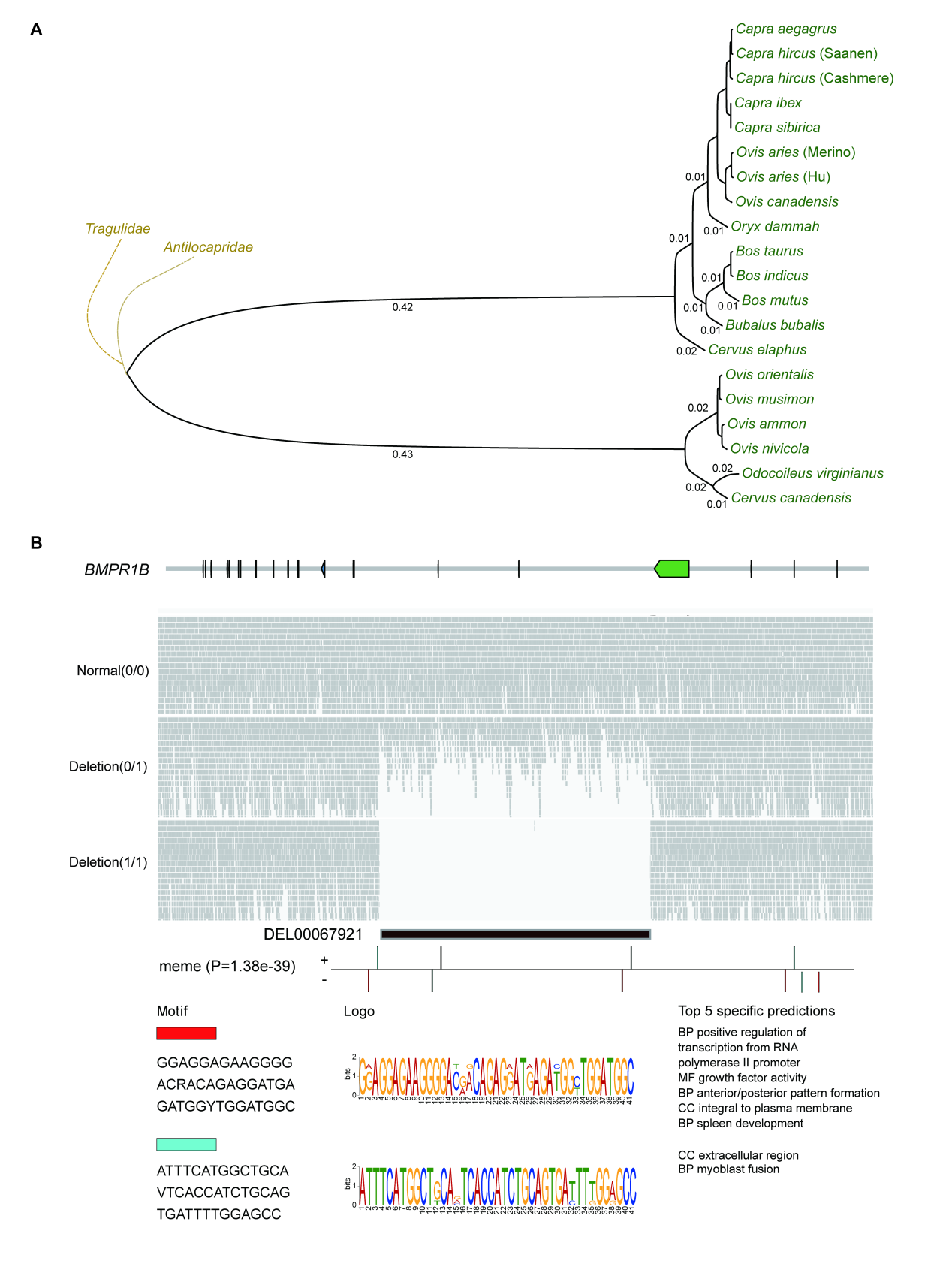


**Fig. S18. Evolution and function analysis of the deletions in *BMPR1B* of goats.** **A** The phylogenetic tree of deletion DEL00067921 identified in goat *BMPR1B* across species within ruminants. **B** The IGV visualization of location, sequence and motifs of DEL00067921 in goat *BMPR1B*, and the GO enrichment analysis of the motifs in DEL00067921. The sequence of DEL00067921 is not shown in the figure because it has a long length of 24,341 bp.


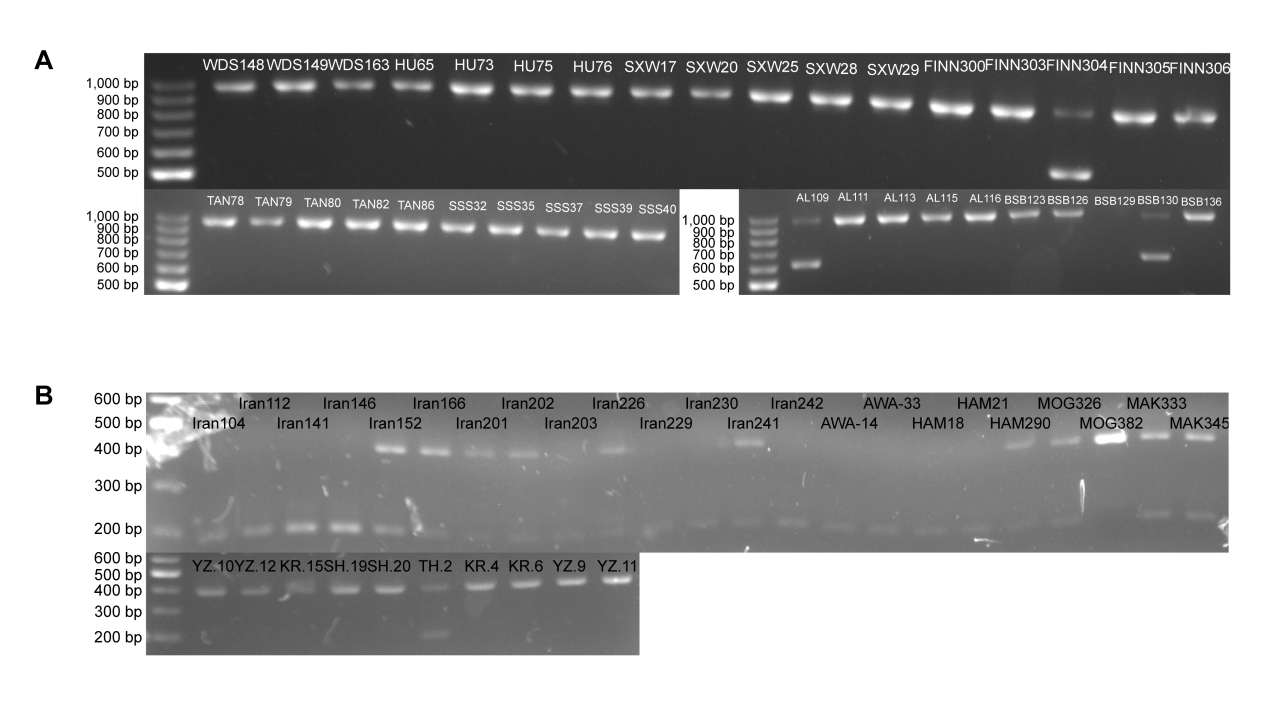


**Fig. S19. Experimental validation of the selected deletions in *BMPR1B* and *BMPR2*.** **A** Electrophoretic gel map shows experimental validation results of the deletion DEL00034481 in *BMPR1B* gene in the prolific and non-prolific sheep individuals. **B** Electrophoretic gel map shows experimental validation results of the deletion SV_w_15555 in *BMPR2* gene in the Asiatic mouflon and Middle Eastern sheep individuals.


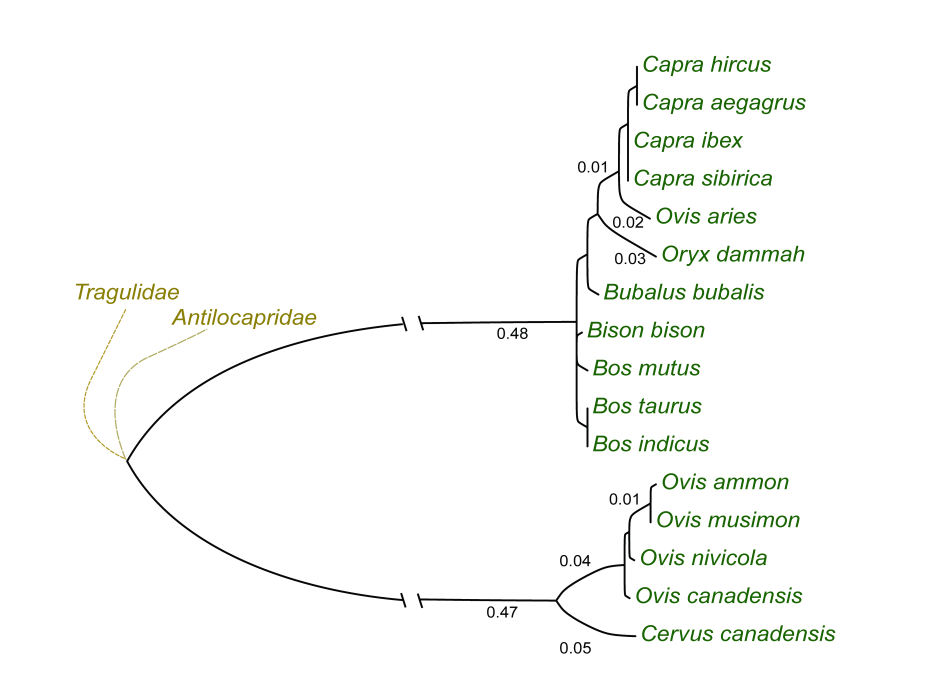


**Fig. S20. Evolution analysis of the deletions in *BMPR1B* of sheep.** The phylogenetic tree of deletion DEL00034481 identified in sheep *BMPR1B* across species of ruminants.

**
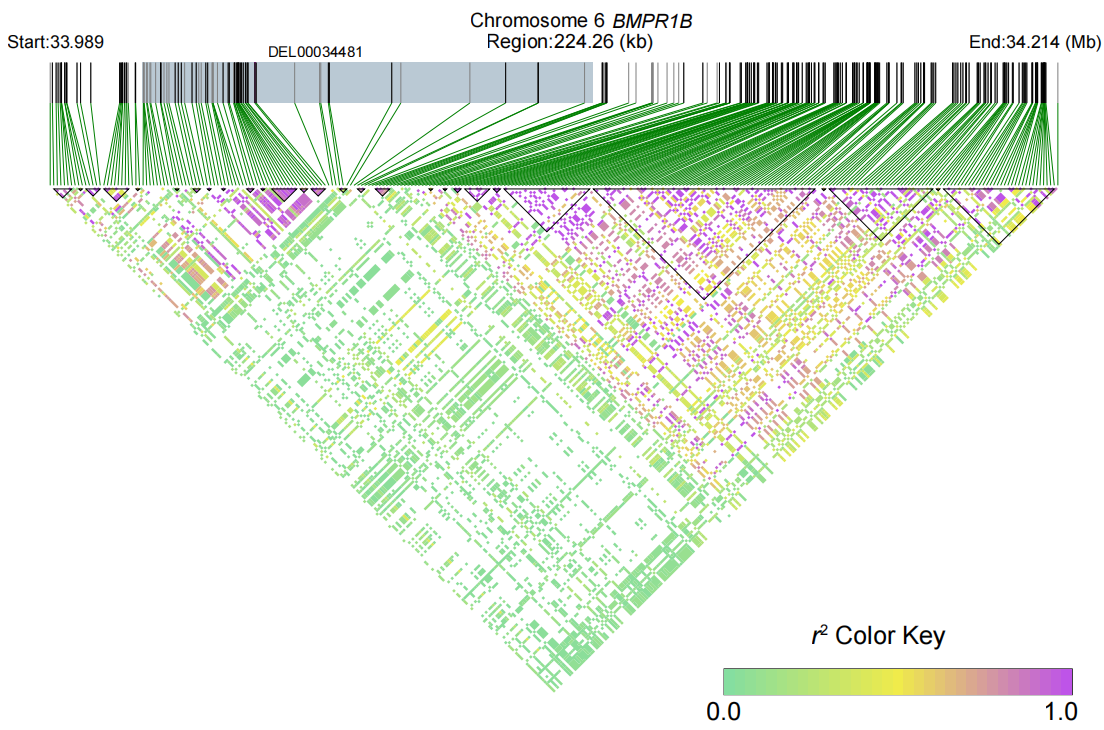
**

**Fig. S21. Linkage disequilibrium and haplotype block analysis of the SVs and SNPs in *BMPR1B* of sheep.** The DEL00034481 deletion is linked to several adjacent selected SNPs but not in linkage with the causal SNP (c.A746G) reported for litter size. The genomic regions under selection in the SNP analysis are shown in light blue. The SNPs located in and outside the haplotype blocks are indicated as gray and black lines, respectively. The DEL00034481 deletion is shown as yellow bar and red border.

**
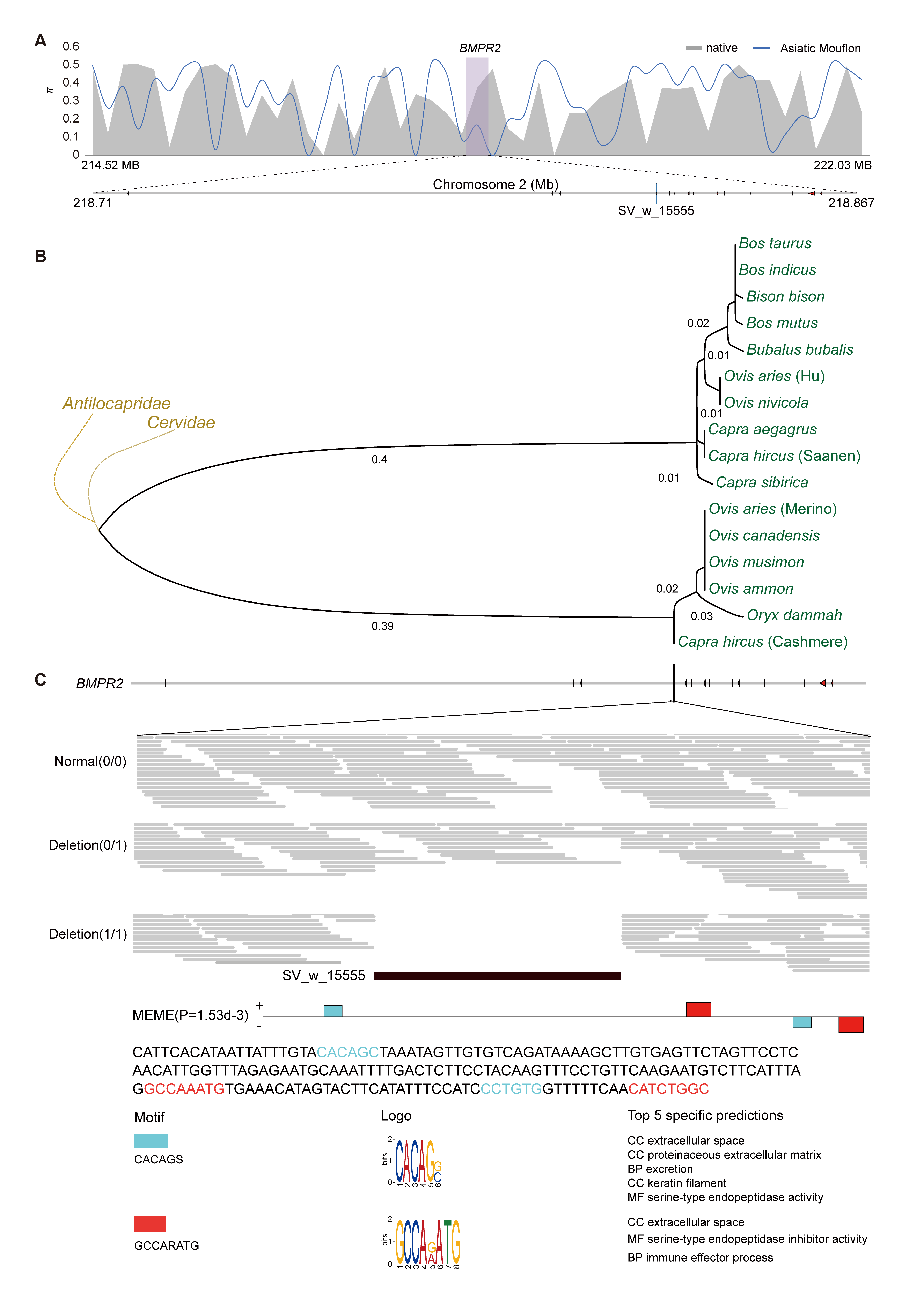
**

**Fig. S22. Evolution and function analysis of the deletions in *BMPR2* of sheep.** **A** Nucleotide diversity across the *BMPR2* locus and adjacent regions in sheep genome. The region of *BMPR2* is shaded in light purple. **B** The phylogenetic tree of deletion SV_w_15555 identified in sheep *BMPR2* across species within ruminants. **C** The IGV visualization of location, sequence and motifs of SV_w_15555 in sheep *BMPR2*, and the GO enrichment analysis of the motifs in SV_w_15555.


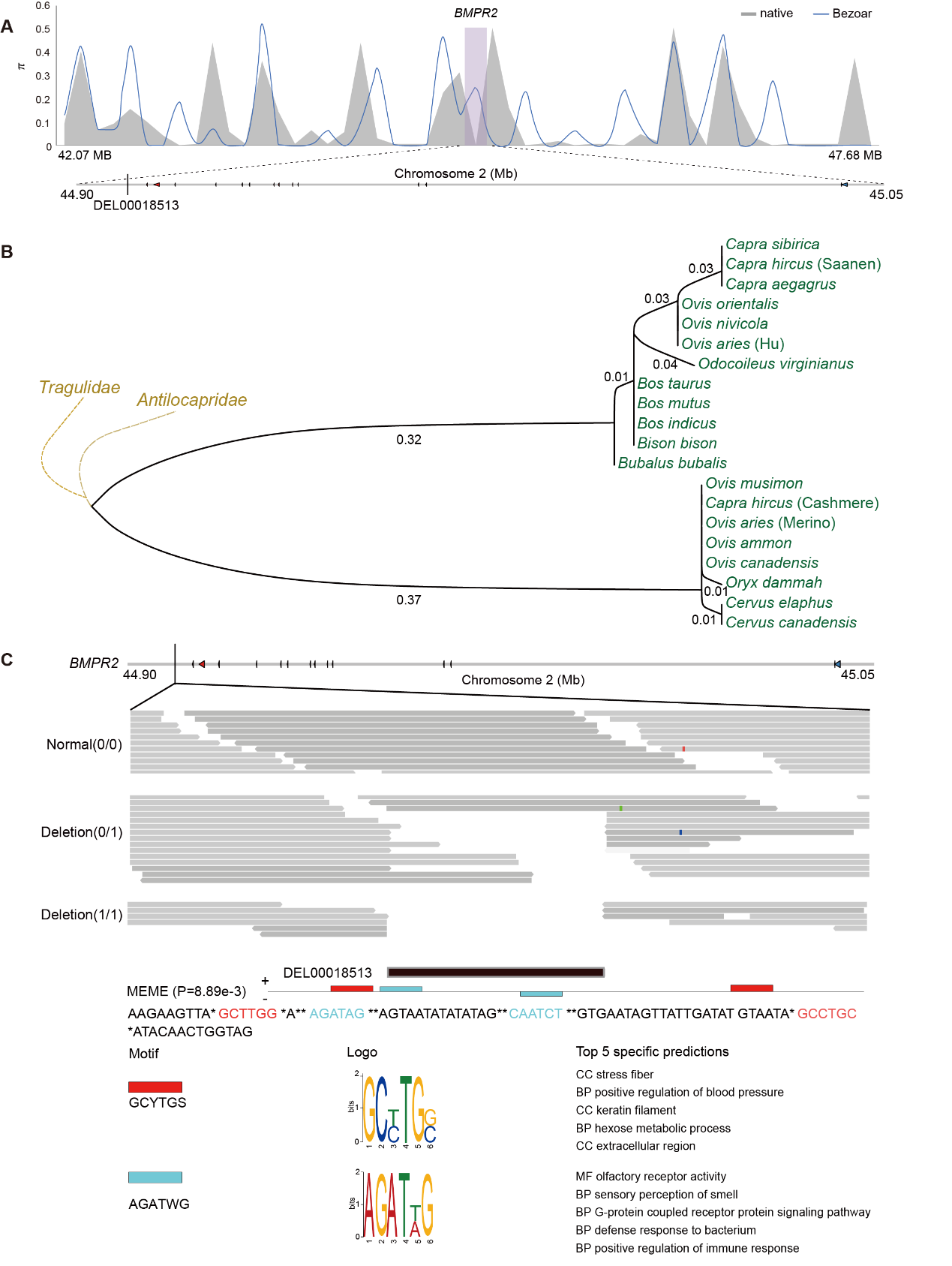


**Fig. S23. Evolution and function analysis of the deletions in *BMPR2* of goats.** **A** Nucleotide diversity across the *BMPR2* locus and adjacent regions in goat genome. The region of *BMPR2* is shaded in light purple. **B** The phylogenetic tree of deletion DEL00018513 identified in goat *BMPR2* across species within ruminants. **C** The IGV visualization of location, sequence and motifs of DEL00018513 in goat *BMPR2*, and the GO enrichment analysis of the motifs in DEL00018513.


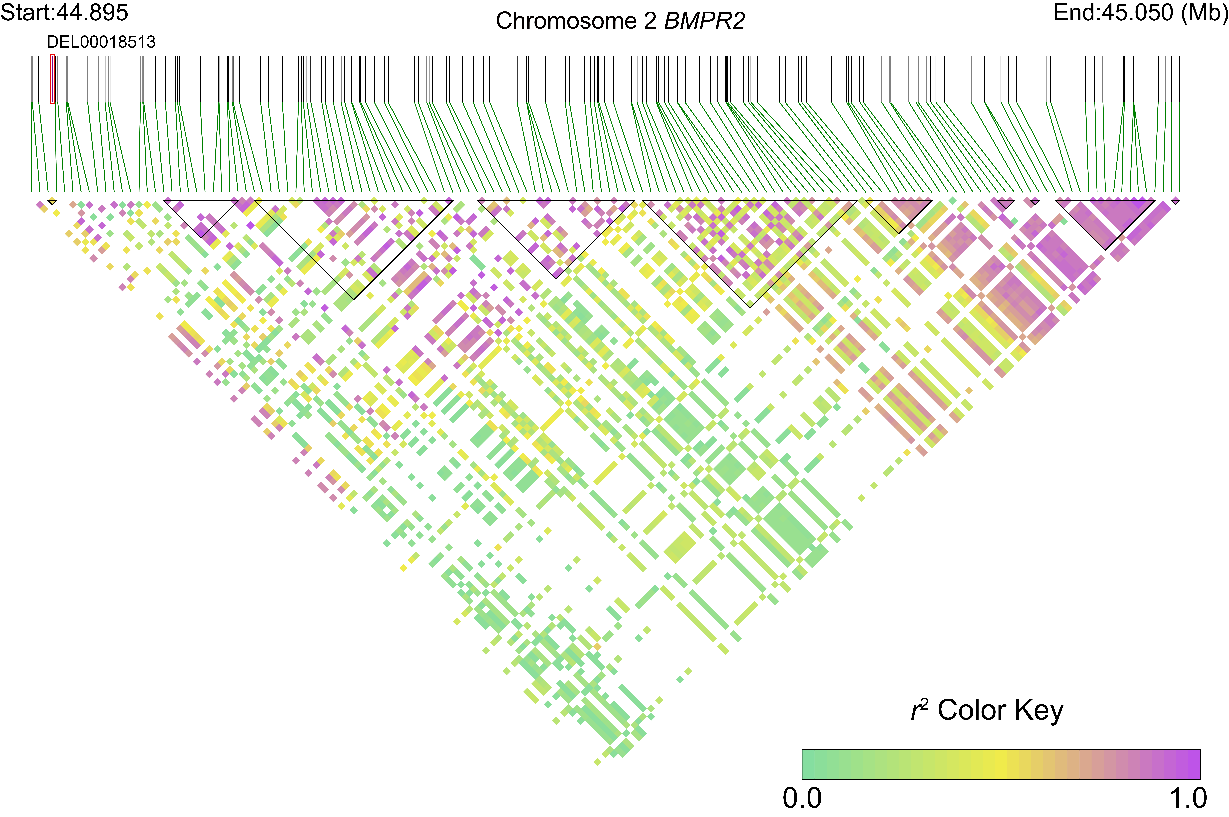


**Fig. S24. Linkage disequilibrium and haplotype block analysis of the SVs and SNPs in *BMPR2* of goats.** The SNPs are not under selection in *BMPR2*, and the DEL00018513 deletion is not linked to any selected SNPs in the gene. The SNPs located in and outside the haplotype blocks are indicated as gray and black lines, respectively. The DEL00018513 deletion is shown as yellow bar and red border.


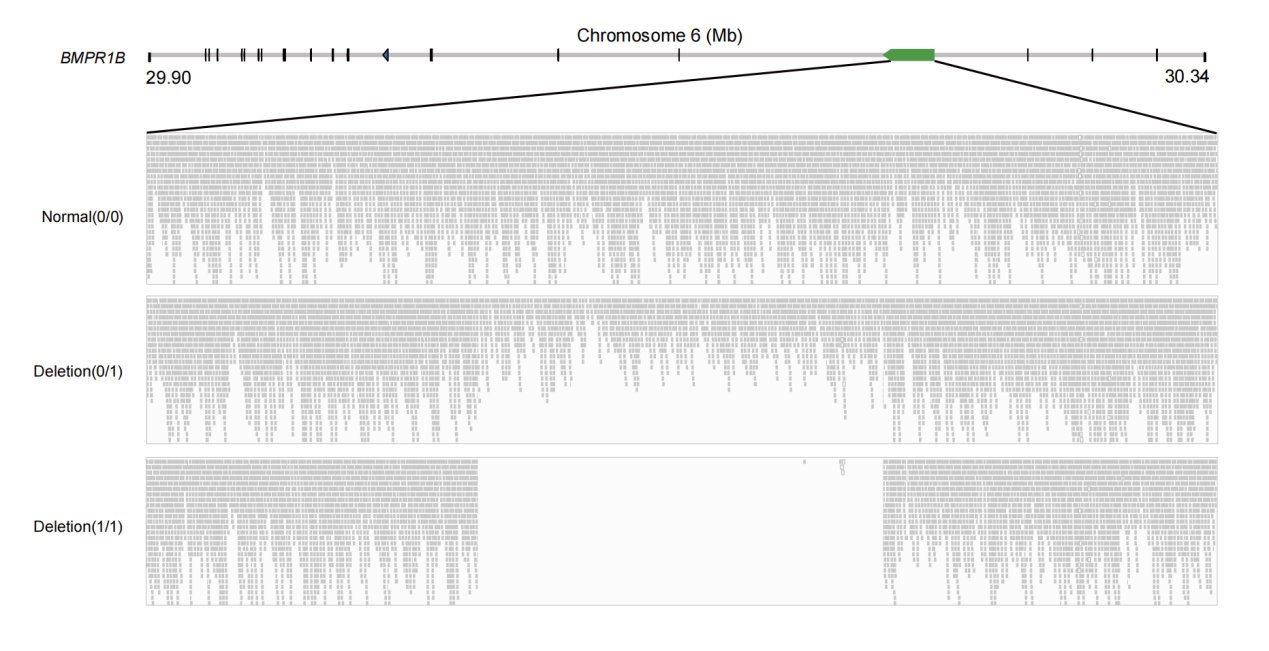


**Fig. S25. The IGV visualization of location and sequence of the deletion in *BMPR1B* significantly (*P* < 0.05) associated with litter size in Yunshang black goats.**


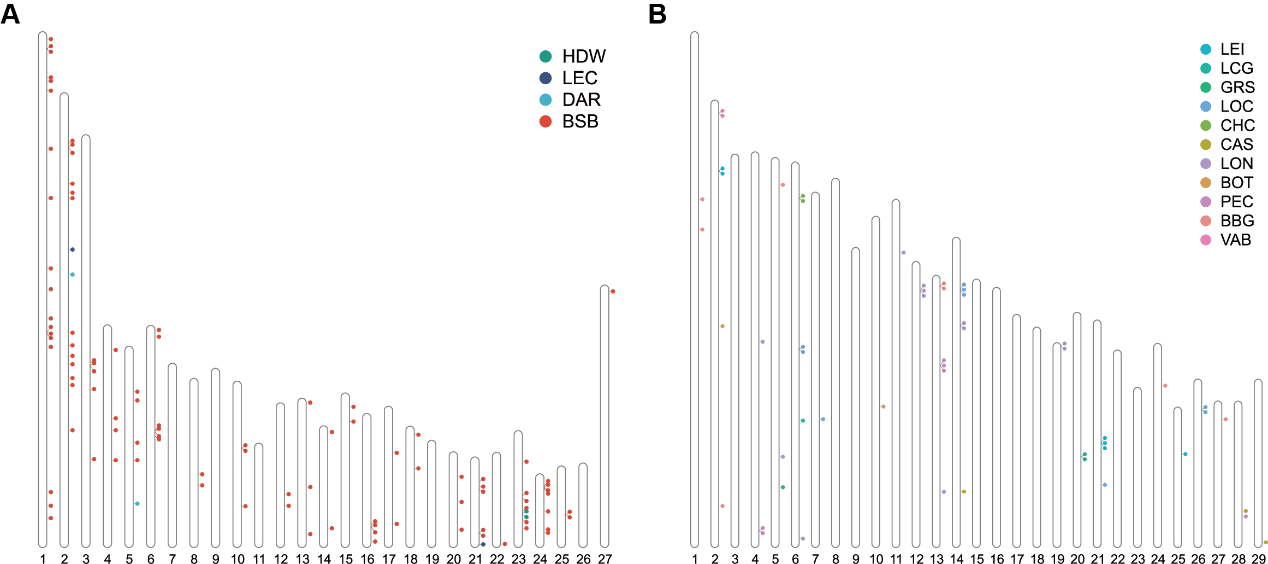


**Fig. S26. SV introgression from wild sheep to domestic sheep populations at chromosomes 1–27 and from wild goat to domestic goat populations at chromosomes 1–29.** For detailed information on the codes of sheep and goat populations shown in the figure, please see Additional file 2: Table S1.


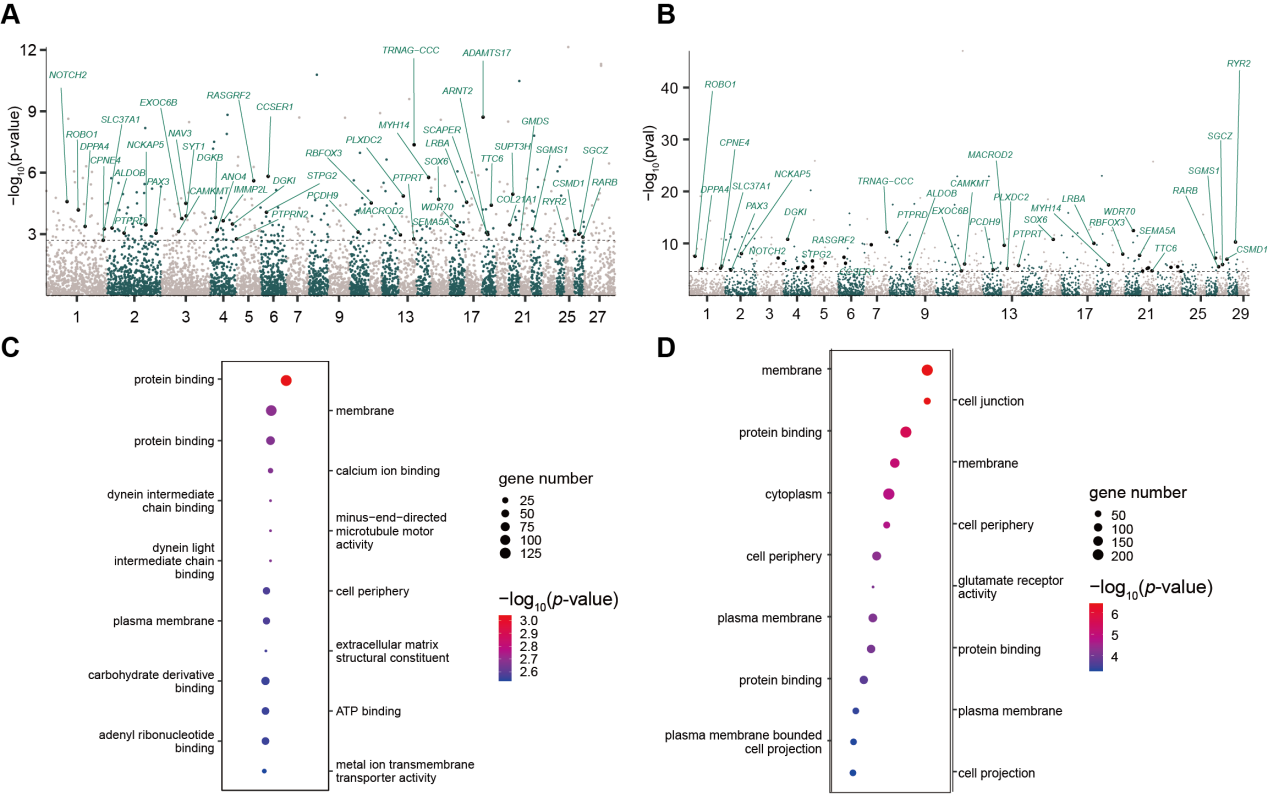


**Fig. S27. Genome-wide environmental analysis of 22 environmental variables and structural variants associated with local adaptation in sheep and goats. A** Genome-wide distribution of -log_10_(*p*-value) using LFMM among native sheep populations. **B** Genome-wide distribution of -log_10_(*p*-value) using LFMM among native goat populations. **C** Top enriched GO terms and KEGG pathways for candidate selected genes associated with environment variables in sheep. **D** Top enriched GO terms and KEGG pathways for candidate selected genes associated with environment variables in goats. **E** Top enriched GO terms and KEGG pathways for 62 commonly selected genes in sheep and goats. In figures **A** and **B**, the horizontal dotted line represents the threshold of top 5% -log_10_(*p*-value). The genes shown in the figures are commonly selected genes in sheep and goats with known functions related to environment adaptation.


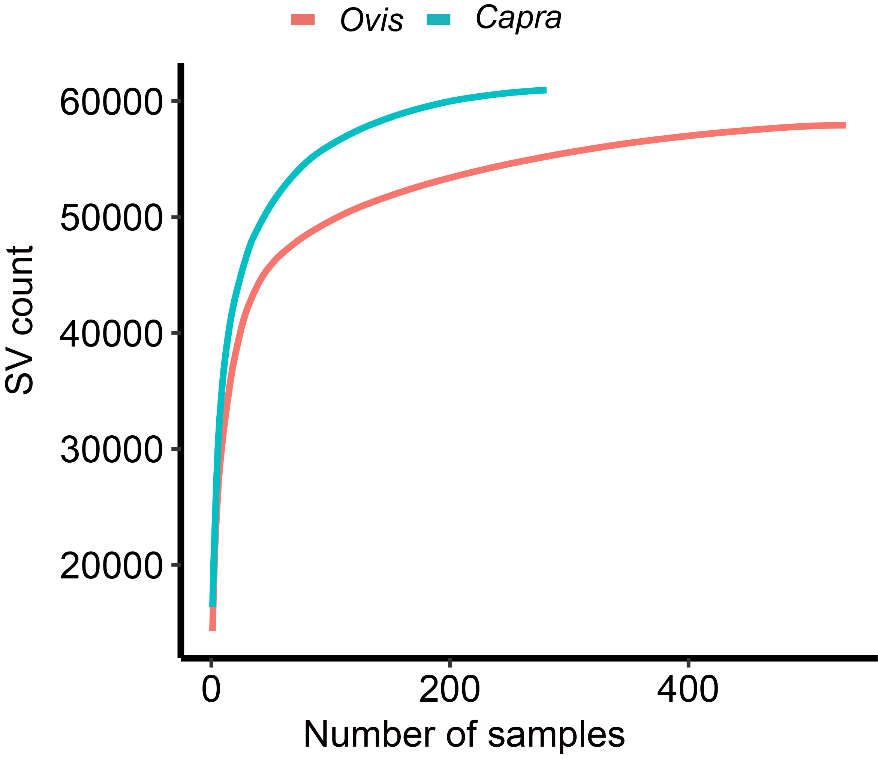


**Fig. S28. The SV numbers identified in the *Ovis* and *Capra* are close to saturation with our sample sizes.**


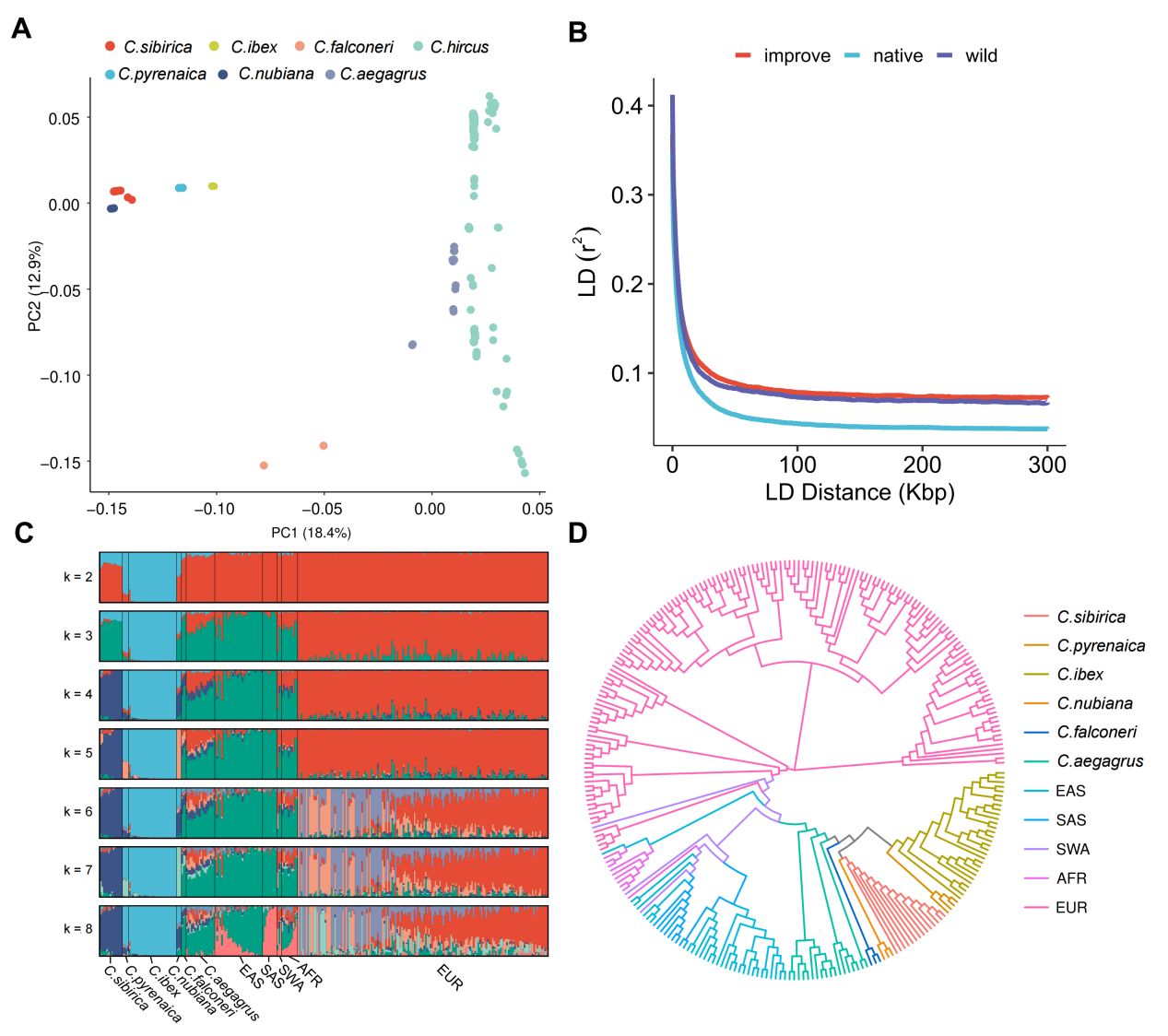


**Fig. S29. Genetic structure of wild and domestic goat populations based on SNPs. A** Principal component analysis (PCA) of wild and domestic goat populations. **B** Phylogenetic tree of wild and domestic goat populations constructed by using the *p*-distances between individuals calculated from SNPs. **C** Population genetic structure analysis of wild and domestic goat populations with assumed genetic clusters *K* = 2–8. **D** Pattern of linkage disequilibrium (LD) decay in the genomes of Bezoar, native and improved populations of domestic goats. In figures **A**, **B** and **C**, EAS indicates East Asia, SAS indicates South Asia, SWA indicates Southwest Asia, AFR indicates Africa and EUR indicates Europe.


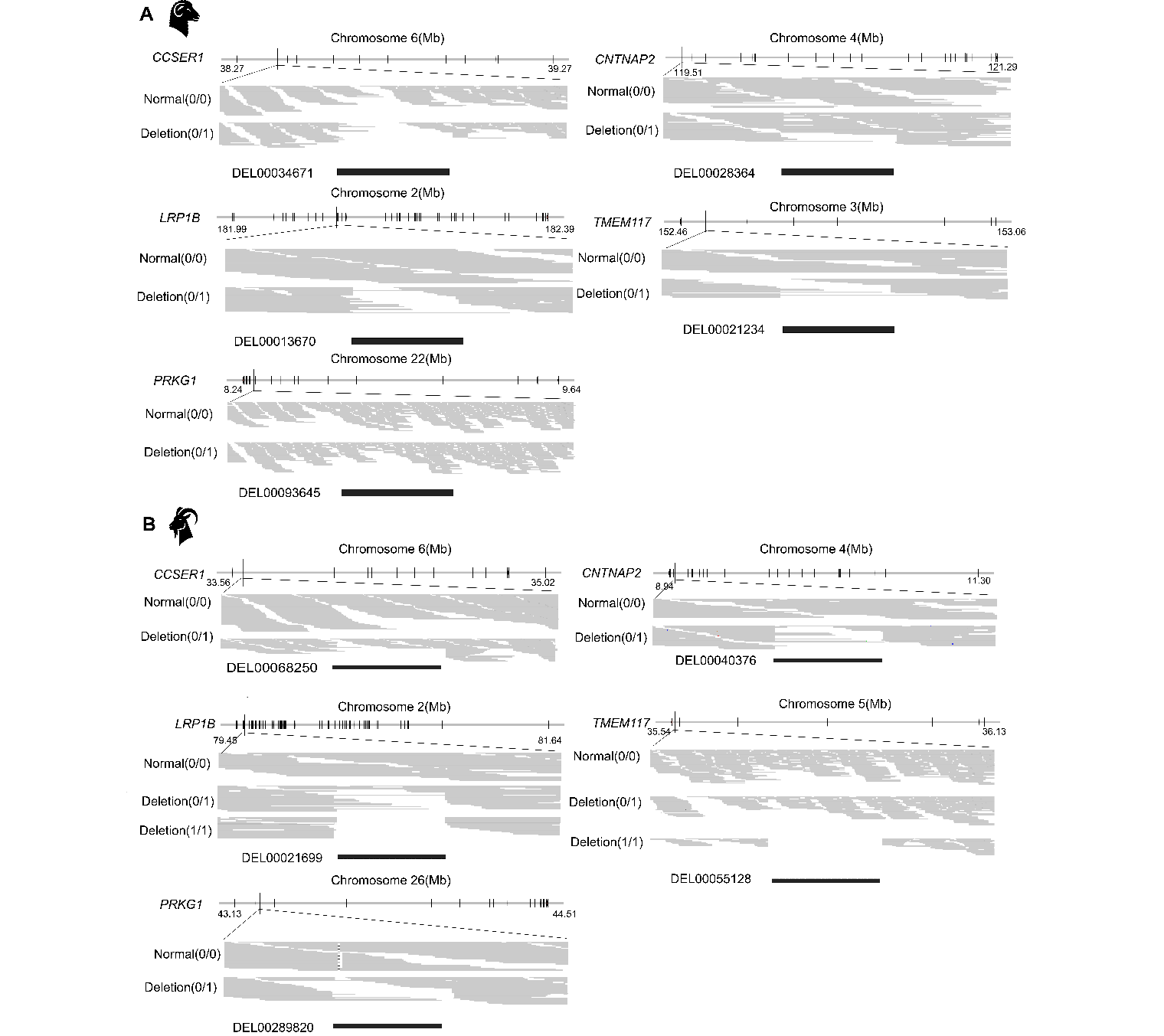


**Fig. S30. The IGV visualizations of locations and genotypes of the SVs in 5 well-known genes associated with production traits. A, B** IGV figures validate the SVs in genes *CCSER1*, *LRP1B*, *TMEM117* for meat and *CNTNAP2* and *PRKG1* for dairy and meat in sheep (**A**) and goat (**B**) genomes.
