# Supplementary material for: Structural variant landscapes reveal convergent signatures of evolution in sheep and goats
Source: Genome Biol. 2024 Jun 6;25:148. doi: 10.1186/s13059-024-03288-6 (PMC11155191; doi:10.1186/s13059-024-03288-6)
Supplement: Supplementary file 3 — Additional file 3. Review history. [file 13059_2024_3288_MOESM3_ESM.docx]

Review History

**First round of review**

**Reviewer 1**

**Were you able to assess all statistics in the manuscript, including the appropriateness of statistical tests used?**

No, I do not feel adequately qualified to assess the statistics.

**Comments to author:**

The Ms by Yang et al. presents an impressive piece of work identifying a set of candidate SVs putatively involved in the domestication process of sheep and goats, among which several are orthologous among species and might result from convergent evolution.
This is an interesting and valuable contribution to the genomics of sheep and goats. However, several concerns have to be considered by the authors.

Main concerns.
- Throughout the Ms, the authors refer to convergent evolution / convergence. They should be more careful when using these terms. First, referring on previous publication, they might define precisely the term 'convergence' with regards to 'parallelism', as both can explain the occurrence of orthologous genomic regions impacted in sheep and goats. Moreover, in the same way that signatures of selection identify 'candidate genes' and not 'selected genes' (because of false positives), it is incorrect to speak about 'convergence' all along the manuscript when orthologous genes are impacted. Most of the time it would be preferable to speak about orthologous genes impacted or candidates, and to what extend they results form convergence or parallelism should be discussed.
- I found that several conclusions should be down toned. Especially the fact that Svs negatively affect gene expression. I agree that the differences in log(Fold change) or FPKM are sometimes significant based on a Wilcoxon test. However (i) this is a global comparison (do not addressing the direct effect of Svs on different alleles of the same genes separately) making it difficult to interpret any difference ; (ii) even when significant the effect is very low, i.e., a few percents (Fig S25, S26) questioning the effect on the phenotype (stronger effects are considered in transcriptomic studies). Thus I don't see the interest of these data (Results section 'impacts of Svs on the gene expression…' and related discussion)
L 657 it is said that the genomes are 'extensively shaped' by introgression. I recommand to moderate this conclusion on the basis of the results found.
L 595 said that there is 'strong evidence of the high reliability of the SVs identified' due to the fact that the PCA based on SVs is consistent with the patterns found on the basis of SNPs analysis. I don't agree with this conclusion, as a large proportion of false genotypes randomly distributed would not affect the differentiation between well structured groups in a PCA.
- L 359-… and Table S30. I don't see what can be said about the fact that some genes have already been found in other studies. We have no indication on the process of selecting the bibliography to find these genes, no indication of wether some of the studies included individuals used in the present one (because WGS were retrieved from public databases), and no indication on the fact that the number of common genes is higher than by chance.
I also have concerns related to the Methods :
- The dataset analysed is obtained by retrieving WGS from public databases, i.e., from different studies (what means studies with different possibles biases, see e.g. https://doi.org/10.1186/s12859-017-1756-z). The authors should demonstrates the absence of batch effect and detail the how they proceeded to (at least) minimize this issue.
- L1037 I don't see why the Jaccard similarity would give evidence for the occurrence of introgression. Such similar alleles could be inherited from a common ancestor in both lineages, and you cannot distinguish between the two on the sole basis of a similarity index.
- L1045-48 : the authors select samples representative of a population/species (i.e. at least 3 replicates), but what about a probable polymorphism. How was this handled to ensure correspondance between genotypes and phenotypes. Also, how are handled possible batch effects that exist when merging data from different projects ? What are the possible biases and their impact on the hypothesis tested ? Same concern with the ATAC-Seq data.

- With regards to the dataset used :
It appears from Table S1 that 160 genomes used were retrieved from NCBI (PRJEB31857, PRJEB37122). The origin of these data is conveniently cited. However it appears that they are under a data sharing statement (see e.g., https://www.ncbi.nlm.nih.gov/bioproject/533273). I think that the authors must give evidence that they have contacted the consortium having produced the data and signed up the data sharing agreement.


Minor points

- in the Figures, the sheep and goat logos are taken from Alberto et al. 2018.I think the authors should get the authorization from Nature Communication and acknowledge the authors.
- I was not able to find Tables S3 - S10 in the supplementary material
- Fig. 3G - please explicit the name of the x-axis on the figure.
- I do not understand Fig 5A. The legend should be much ore explicit and/or the Figure redrawn in a clearer way
- throughout the text : 'selective signature' → 'selection signature'
- Fig. 8 is cited in the text before Fig. 7
- Fig. 6G (and S19) not so clear, please adjust the color-key
- L 533 - 'these findings …' sentence not clear. Do you mean 'lower than' ?
- L 580 … I don't see the difference between what is said in this sentence and and in L 577.
- L 608 in some of the references cited at least some genes have been identified on the basis of SV analyses.
- Paragraph beginning L 669 - As it is about data quality on which the whole study rely, I would shift this paragraph at the beginning of the discussion.
- L 923-24. Why was this study dine only on goats ? This does not prejudge what may happen on sheep. You should repeat the same analysis on sheep.
- L950-51: please give details on how VCFtools estimates the global Fst
- L 955-56 : Fst are estimated between populations with contrasting phenotypes, but we can see in Table S27 that some generalist breeds are referenced in several groups (i.e.traits). How was it handled?
- L 1024-25: not clear if there is polymorphism in the populations and how it is handled.
- L 1082 'mutual SV'. I don't understand what you are referring to.

**Reviewer 2**

**Were you able to assess all statistics in the manuscript, including the appropriateness of statistical tests used?**

Yes, and I have assessed the statistics in my report.

**Comments to author:**

Yang et al. reported a very comprehensive investigation of convergent evolution between two closely related domestic animal species, sheep and goat, based on structural variants (SVs) at the whole-genome level. This is a very interesting study from several aspects: (1) as I know, this is the one of a few, or perhaps the first, SV-based convergent genomic evolution between phylogenetically related mammals with such as a large sample size; (2) samples of the two species are from a worldwide geographic origins and with similar artificial selective pressures for similar phenotypes such as milk, meat and fiber; (3) populations of the two domestic species might have experienced wild introgressions, which might lead to similar consequences like local adaptation; (4) most interestingly, they characterized one SV in one functional gene which explained around 10% of the phenotypic variance, and (5) Last, the analyses in the work are very thorough and detailed. However, I have several major comments and minor comments for improving this manuscript. In summary, I suggest that this manuscript could be accepted for publication after resolving the below comments.

1． Convergent evolution between sheep and goat has been studied previously based on genome-wide SNPs [Nature communications 9: 813 (2018)]. I think this study is a significant advance in SV-based convergent evolution, but I wonder what were the differences in the results and conclusions between this study and the earlier investigation? The similarity and differences in results should be discussed in more details. Also, I would like to see the overlapping signals detected between the two studies and to see if there is link between the SNPs detected in the previous study and the SVs detected here?
2． A chromosome-level genome assembly of Asiatic mouflon was reported. This is a useful resource for the future studies on sheep domestication. I have two points here: (1) although table 1 has summarized the main parameters for the assembly, but I would I like to see a comparison of the parameters between this assembly and the published assemblies of sheep and other related species. Also, a short discussion on the comparison would make sense, at least for their utilization in domestication analyses etc. (2) minor discussion on the future T2T assembly in charactering SVs would be necessary, whatever for Asiatic mouflon and domestic sheep.
3． Some caveats in the data should be pointed out as well. There are only one or very small sample sizes for some wild sheep or goat species, which can be due to the limitation to access a large sample size of some wild animals, in particular some wild protected animals. The bias and effect on SV identification and characterization due to small sample size should be discussed. In addition, the difference in sequencing depth may also lead to biases in SV characterization.
4． As to the SV-based introgression analyses, it would be very necessary to justify the correct utilization of the method in the analyses. For example, how this method has been used in the similar analyses of introgression between other species, and why this method is suitable for the detection of introgression between wild and domestic sheep/goat species.

Minor comments:
1. Too many references. I suggest to omit some unnecessary references.
2. The whole manuscript reads well, but some sentences could be improved by a native English speaker.
3. Too many figures. Some parts in some figures could be moved to supplementary information.

**Reviewer 3**

**Were you able to assess all statistics in the manuscript, including the appropriateness of statistical tests used?**

Yes, and I have assessed the statistics in my report.

**Comments to author:**

Review of "Structural variant landscapes reveal convergent signature of evolution in sheep and goats" by Yang et al.

This study reports the outcomes of an in-depth analysis of structural variants across multiple breeds/origins for both sheep and goats. Additionally, long-read sequencing was conducted for the generation of an Asiatic mouflon de novo assembly. SVs were interrogated for characterization relating to genetic diversity, chromosomal distribution, gene links, QTLs, transposable elements, and impact on gene expression and regulatory elements. This allowed for the linkage with known phenotypes and evolutionary convergence. This data can be a basis for further hypothesis testing and comparison to other livestock species. Overall, the strengths of this manuscript include the breadth of sample inclusion and utilization of multiple SV callers to mitigate software biases. However, there are a few weaknesses: while the selection analysis was a large portion of the manuscript, it feels weak. A more sophisticated methodology could be undertaken as it relates to the FST outliers' analysis, and there was no control for demographic history.

General Comments:
- Please review manuscript, figure legends, and Additional File for grammatical and spelling errors.
- Ensure format of abbreviations are the same throughout (example: non-SV-genes vs. nonSV-genes).
- For some of the figures, it is advised to change the color palette to colorblind friendly, e.g., substituting green for a different color.
- An increase in background on goat and sheep divergence/comparison would be helpful.
- The results section addressing the de novo assembly could be expanded, particularly as it relates to the methods.
- Why were hotspots intersected with QTLs? Was there an expectation that multiple variant loci would be selected for production traits?
- Were the p-values from the GO output corrected for multiple testing?
- For the de novo genome assembly statistics. Per the Earth Biogenome project's standards, it is recommended to include a kmer completeness analysis, aiming for greater than 90%.
- For the genetic structure analysis. Please provide background information on why the values K=5 and K=6 were selected for plotting. Could you provide cross-validation values to justify.
- The deletion analysis feels a bit speculative. While an intronic deletion may be an effect, more data is needed. Is there specific binding sites that show evidence of conservation or other signals of enhancers?
- Is the expression data is from different samples? If so, it is unclear how it is then integrated with the SV data

Specific Comments:
Line 63 : Recommend reordering words, placing "de novo" after "assembled a": "Here. We assembled a de novo high-quality…" .
Line 65: Please be more explicit in what ">15x" reference.
Line 67-68: It is indeed a large and comprehensive dataset/analysis; however, it is recommended to temper the verbiage to being less absolute. E.g., "large dataset" and "one of the most comprehensive".
Line 69: The word "legacy" seems out of place. Suggest a different word choice or reworking sentence to remove.
Line 102: What is the "PAV" distribution. Please spell out PAV before abbreviation.
Lines 105-109: Any statistics available to quantify these statements?
Line 113: Can you break out the number of samples to quantify the number of wild and domestic?
Line 115: Please check sheep values. Per Supplemental Table S1 and S3, mean depth for sheep is 18.32 (15.02-30.11). Coverage values match.
Lines 116-117: Please goat values. Per Supplemental Table S1 and S3, mean depth values is 20.04 (5.97-41.06).
Line 114 and 117: It is stated that a minimum depth of >15x for the study. However, there are a number of goat samples below that value.
Line 149: To ease reading comprehension, please spell out deletion and duplication before abbreviation.
Line 184: Suggest changing "…the numbers in the introns…" to "intronic".
Line 162: What is a "hotspot"? A clearer definition would be helpful.
Line 188: Were most of these genes with SVs in the intron? What was the proportion of intron/exon? How many genes had an SV overlapping with an exon?
Line 218: Why would the LD be high in the wild species? This is the opposite of what I would have expected.
Line 219: Was the pi based on SVs or SNPs? Accordingly, what does the LD pattern look like with SNPs?
Line 335: here is limited overlap between SNP and SV FST outlier. This is a sign of power, genotyping error, etc. Would expect a true selected signal to have a long haplotypic signal that is also well tagged by SNPs.
Line 404: Space needed: "DEL00034481deletion" to "DEL00034481 deletion".
Line 407: IGV visualization is not true "validation", but nonetheless makes a clear representation of the SV.
Line 533-534: Sentence starting with "These findings…". Should the sentence end with "… low expression than non-SV-genes"?
Line 539: What is the definition of Peak vs non-peak SV? This seems like useful data that could be used to annotate potential enhancer regions.
Line 584: Suggest reworking sentence starting with "The amount of…". E.g., "The high number of included genomes in our study (532 sheep and 442 goats) could ensure…".
Line 593: Include oxford comma following "genetic structure".
Line 634: Include oxford comma following "gene expression".
Line 650: Include oxford comma following "apple".
Line 654: Strike "good".
Line 658: Change analysis to analyses.
Line 662: Include oxford comma following "conformation".
Line 673: Include oxford comma following "Delly".
Line 686: Include oxford comma following "graph".
Line 691: Strike "on".
Lines 729-730: Please check values as they are different than those reported in Lines 115-118.
Line 738: Include oxford comma following "small intestine".
Line 815: It is recommended that the newest version of BUSCO (v5.4.7) and database (odb10) be utilized.
Line 934: Include oxford comma following "early development".
Line 1026: Hisat2 to HISAT2. DeSeq2 to DESeq2.
Line 1051: Hisat2 to HISAT2. Please include HISAT2 version.
Line 1052: Please include versions of featureCounts and DESeq2. DeSeq2 to DESeq2.
Tables:
Table 2: The number of translocations are unlikely to be real. Are the duplicated/mapability issues in the assemblies used?
Table 3: Have same justification (either right or centered) for both rows.
Figures:
Figure 1: Panels B and C: Please include accounted variance for each principal component.
Figure 1: Panel D and E: As mentioned above, please provide justification for K selection and include cross-validation values for K values. Figure legend states K 2-5 and 2-6, when only K 2,5 and 2,6 are plotted.
Figure 2: Panel C: Why is the limit 1kb?
Figure 2: Panel E and F: Figures are difficult to discern differences and trends across the groups. Suggest a different format of plot to better delineate the groupings.
Figure 4: Panel H: Value for C. sibirica-C.nubiana is not centered in box.
Figure Legends:
Line 1587: Additional fle to Additional File.
Line 1632: Additional fle to Additional File.
Line 1637: Additional fle to Additional File.
Line 1670: Additional fle to Additional File.
Line 1697: Additional fle to Additional File.

**Authors Response**

**Point-by-point responses to the reviewers’ comments:**

Reviewer reports:
Reviewer #1:
The Ms by Yang et al. presents an impressive piece of work identifying a set of candidate SVs putatively involved in the domestication process of sheep and goats, among which several are orthologous among species and might result from convergent evolution.
This is an interesting and valuable contribution to the genomics of sheep and goats. However, several concerns have to be considered by the authors.
We have addressed all your concerns. Please see the point-to-point responses detailed below.

Main concerns.
- Throughout the Ms, the authors refer to convergent evolution / convergence. They should be more careful when using these terms. First, referring on previous publication, they might define precisely the term 'convergence' with regards to 'parallelism', as both can explain the occurrence of orthologous genomic regions impacted in sheep and goats. Moreover, in the same way that signatures of selection identify 'candidate genes' and not 'selected genes' (because of false positives), it is incorrect to speak about 'convergence' all along the manuscript when orthologous genes are impacted. Most of the time it would be preferable to speak about orthologous genes impacted or candidates, and to what extend they results form convergence or parallelism should be discussed.
[1.1] Good comment! The terms of convergence (or convergent evolution) and parallelism (or parallel evolution) do have similar meaning, resulting in misinterpretation and confusion [1]. The definition of convergent evolution and parallel evolution can apply at various scales on the tree of life [2-4]. In this study, species in the same genus were closely related and could be parallel evolution, whereas those in different genera (e.g., sheep and goats) were distantly related and could be defined as convergent evolution.

At the molecular level, phenotypic changes of particular traits in different species can arise from the same/different molecular pathways, genes or nucleotides, which correspond to parallelism/convergence [5, 6]. Since orthologous genes across species are currently best characterized [7], we considered molecular parallelism and molecular convergence at the gene level in this study. Specifically, candidate selected genes identified for the same (or similar) traits of sheep and goats were compared, and the common genes (i.e., orthologous genes) between the two species were defined as molecular parallelism. This enabled us to evaluate the extent of molecular parallelism/convergence underlying the convergent evolution (e.g., convergent selection) of sheep and goats.

Based on the definition of parallel/convergent evolution and molecular parallelism/convergence, we revealed a limited extent of molecular parallelism (5.07% for sheep genes and 7.29% for goat genes) accounting for the convergent signatures of evolution in sheep and goats, which is consistent with previous findings that parallelism was less detected than convergence [8]. This suggested parallel gene-use to some extent by sheep and goats, and provided a remarkable example of molecular parallelism between ruminant species.

Following your comment, we changed “selected genes” to “candidate genes” or “candidate selected genes” throughout the manuscript, and changed “common genes” or “overlapped genes” to “orthologous genes” where applicable. We also added the contents and brief discussion about convergence and parallelism to the relevant part of the Results and Discussion section, respectively. Please see lines 402-417 and 706-712.

Reference:
1.Cerca, J. Understanding natural selection and similarity: Convergent, parallel and repeated evolution. Mol Ecol. 2023;32:5451–62.
2.Elmer KR, Meyer A. Adaptation in the age of ecological genomics: insights from parallelism and convergence. Trends Ecol Evol. 2011;26:298–306.
3.Lescak EA, Bassham SL, Catchen J, Gelmond O, Sherbick ML, von Hippel FA, et al. Evolution of stickleback in 50 years on earthquake-uplifted islands. Proc Natl Acad Sci U S A. 2015;112:E7204–12.
4.Westram AM, Galindo J, Alm Rosenblad M, Grahame JW, Panova M, Butlin RK. Do the same genes underlie parallel phenotypic divergence in different Littorina saxatilis populations? Mol Ecol. 2014;23:4603–16.
5.Dong Y, Wang YZ. Seed shattering: from models to crops. Front Plant Sci. 2015;6:476.
6.Rosenblum EB, Parent CE, Brandt EE. The molecular basis of phenotypic convergence. Annu Rev Ecol Evol Syst. 2014;45:203-26.
7.Woodhouse MR, Hufford MB. Parallelism and convergence in postdomestication adaptation in cereal grasses. Philos Trans R Soc B. 2019;374:20180245.
8.Pickersgill B, Parallel vs. Convergent Evolution in Domestication and Diversification of Crops in the Americas. Front Ecol Evol. 2018;6:56.

- I found that several conclusions should be down toned. Especially the fact that Svs negatively affect gene expression. I agree that the differences in log(Fold change) or FPKM are sometimes significant based on a Wilcoxon test. However (i) this is a global comparison (do not addressing the direct effect of Svs on different alleles of the same genes separately) making it difficult to interpret any difference ; (ii) even when significant the effect is very low, i.e., a few percents (Fig S25, S26) questioning the effect on the phenotype (stronger effects are considered in transcriptomic studies). Thus I don't see the interest of these data (Results section 'impacts of Svs on the gene expression…' and related discussion)
[1.2] As suggested, we removed all relevant contents about the impact of SVs on gene expression from the paper.

L 657 it is said that the genomes are 'extensively shaped' by introgression. I recommend to moderate this conclusion on the basis of the results found.
[1.3] Following your comment below (i.e., - L1037 I don't see why the Jaccard similarity ...) which is related to the Methods of introgression analyses and the comment from reviewer 2, we used a new method based on the allele frequency of each SV [1, 2] to perform introgression analyses for domestic sheep/goat populations and their congeneric wild relatives. This method was also used to detect genetic introgression from wild cattle to domestic cattle populations [2] and from other Bos species to indicine cattle [1] based on SV variants. On the basis of the new results, we moderated this conclusion associated with introgression. We found that introgressed SVs were most observed in Bashibai sheep and Longlin goat, and most enriched on chromosomes 1, 2, 23, and 24 of the sheep genome and 2, 6, 13, and 14 of the goat genome (Additional file 1: Fig. S26). Please see lines 555-566 and 737-739.

Reference:
1.Dai X, Bian P, Hu D, Luo F, Huang Y, Jiao S, et al. A Chinese indicine pangenome reveals a wealth of novel structural variants introgressed from other Bos species. Genome Res. 2023;33(8):1284-98.
2.Xia X, Zhang F, Li S, Luo X, Peng L, Dong Z, et al. Structural variation and introgression from wild populations in East Asian cattle genomes confer adaptation to local environment. Genome Biol. 2023;24(1):211.

L 595 said that there is 'strong evidence of the high reliability of the SVs identified' due to the fact that the PCA based on SVs is consistent with the patterns found on the basis of SNPs analysis. I don't agree with this conclusion, as a large proportion of false genotypes randomly distributed would not affect the differentiation between well structured groups in a PCA.
[1.4] Following your comment, we corrected this sentence to “This provides evidence that the SVs identified here could largely recapture the known population structures in sheep and goats”. Please see lines 664-665.

- L 359-… and Table S30. I don't see what can be said about the fact that some genes have already been found in other studies. We have no indication on the process of selecting the bibliography to find these genes, no indication of whether some of the studies included individuals used in the present one (because WGS were retrieved from public databases), and no indication on the fact that the number of common genes is higher than by chance.
[1.5] We summarized the common candidate genes identified in sheep and goats which have been also reported previously in human, sheep, goats and other animals. The main reasons are two-folds. On the one hand, this could enable us to distinguish previously reported genes and novel genes among all candidate genes. On the other hand, this provided evidences that the functions of some candidate genes (i.e., previously reported genes) were actually linked to domestication or particular traits, because their gene functions have been demonstrated in human or other animals in previous studies. These previously reported candidate genes could be considered as the most plausible genes associated with the domestication and trait selection of both sheep and goats.

We added the process of selecting the bibliography to find these genes in the Methods section. We also clarified that the previous studies presented in Table S31 (previous Table S30) didn’t include the same sheep/goat individuals as the present study. Please see lines 1245-1257.

We could not perform permutation test to check if the number of overlapped genes listed in Table S31 is higher than by chance. There are two reasons.. First, the previous studies cited in Table S30 usually had a different gene pool and different panel of selected genes from different species, and most studies only reported a small number of selected genes, which made it very difficult to find suitable study to implement the permutation test between our study and previous study. Second, we just wanted to see if particular candidate gene identified in this study has been reported previous, thus providing evidence to support the function of a specific candidate gene. We didn’t intend to see of there was a significant amount of overlapped genes between our study and a previous study.

I also have concerns related to the Methods :
- The dataset analysed is obtained by retrieving WGS from public databases, i.e., from different studies (what means studies with different possibles biases, see e.g. https://doi.org/10.1186/s12859-017-1756-z). The authors should demonstrates the absence of batch effect and detail the how they proceeded to (at least) minimize this issue.
[1.6] Batch effects usually refer to unexpected variations in data generated from technical sources (e.g., machine and experimenter) that can confound data analysis, yet the task of evaluating batch-associated variances and removing batch effects is complex [1-4].

In this study, although we didn’t mention batch effects in the previous version of the manuscript, our data collecting, processing and analyzing strategies should have minimized the issue of batch effects. First, the sequencing depths of all domestic sheep/goat and wild sheep genomes and most of wild goat genomes collected in this study are larger than 15×. Second, uniform criteria were used to filter raw reads and align high-quality reads to reference genome for all the collected genomic data of sheep/goats. Third, SVs were identified for each sample by three independent tools and integrated by SURVIVOR, and were genotyped jointly with all the sheep/goat sample by SVtyper and annotated with read coverage by Duphold. Lastly, we removed SV sites with missing genotype rate greater than 0.25 for population structure analyses, and used more robust methods to perform whole-genome selection tests (e.g., DISV and FST methods for domestication and PBS method for particular traits) and introgression analyses (e.g., method based on the allele frequency of each SV in the domestic populations and wild species) in the revised version. All the above strategies (e.g., reducing the difference in genome sequencing depths of samples, quality control for raw reads, alignments and analyzed SV data, SV calling with single sample and subsequently integrated SVs with all the samples, and robust analysis methods to control false positives and demographic factors) could have minimized the batch effects in our integrated SV data and corresponding results base on previous investigations [5, 6].

We added a concise discussion about the potential batch effects in our data to the Discussion section and cited relevant reference papers in the revised version. Please see lines 769-777.

Reference:
1.Tom JA, Reeder J, Forrest WF, Graham RR, Hunkapiller J, Behrens TW, et al. Identifying and mitigating batch effects in whole genome sequencing data. BMC Bioinformatics. 2017;18(1):351.
2.Goh WWB, Yong CH, Wong L. Are batch effects still relevant in the age of big data? Trends Biotechnol. 2022;40(9):1029-40.
3.Goh WWB, Wang W, Wong L. Why Batch Effects Matter in Omics Data, and How to Avoid Them. Trends Biotechnol. 2017;35(6):498-507.
4.Leek JT, Scharpf RB, Bravo HC, Simcha D, Langmead B, Johnson WE, et al. Tackling the widespread and critical impact of batch effects in high-throughput data. Nat Rev Genet. 2010;11(10):733-9.
5.Tom JA, Reeder J, Forrest WF, Graham RR, Hunkapiller J, Behrens TW, et al. Identifying and mitigating batch effects in whole genome sequencing data. BMC Bioinformatics. 2017;18(1):351.
6.Seo S, Park K, Lee JJ, Choi KY, Lee KH, Won S. SNP genotype calling and quality control for multi-batch-based studies. Genes Genom. 2019;41(8):927-39.

- L1037 I don't see why the Jaccard similarity would give evidence for the occurrence of introgression. Such similar alleles could be inherited from a common ancestor in both lineages, and you cannot distinguish between the two on the sole basis of a similarity index.
[1.7] We used a new method based on the allele frequency of each SV [1, 2] to perform introgression analyses for domestic sheep/goat populations and their congeneric wild relatives. This method can strictly exclude false positive signals of introgression resulting from common ancestor, and was also used to detect genetic introgression from wild cattle to domestic cattle populations [2] and from other Bos species to indicine cattle [1] based on SV variants. The description of the new method has been added to the Methods section. Please see lines 1156-1169.

Reference:
1.Dai X, Bian P, Hu D, Luo F, Huang Y, Jiao S, et al. A Chinese indicine pangenome reveals a wealth of novel structural variants introgressed from other Bos species. Genome Res. 2023;33(8):1284-98.
2.Xia X, Zhang F, Li S, Luo X, Peng L, Dong Z, et al. Structural variation and introgression from wild populations in East Asian cattle genomes confer adaptation to local environment. Genome Biol. 2023;24(1):211.

- L1045-48 : the authors select samples representative of a population/species (i.e. at least 3 replicates), but what about a probable polymorphism. How was this handled to ensure correspondance between genotypes and phenotypes. Also, how are handled possible batch effects that exist when merging data from different projects ? What are the possible biases and their impact on the hypothesis tested ? Same concern with the ATAC-Seq data.
[1.8] We removed all relevant contents about the impact of SVs on gene expression from the paper in the revised version.

For the ATAC-Seq data, we used new data of hypothalamus, rumen, heart, lung, liver, duodenum, spleen and adipose of sheep from NCBI-SRA in the revised version. All the ATAC-Seq data of sheep or goats are from the same project (Additional file 2: Table S45), thus batch effect should not exist in these data. Also, whole-genome sequencing data of the same sheep samples as the ATAC-Seq data are also publicly available (Additional file 2: Table S46), which enabled us to obtain corresponding SVs to test the impact of SVs on ATAC peaks. Please see lines 1172-1179.

- With regards to the dataset used :
It appears from Table S1 that 160 genomes used were retrieved from NCBI (PRJEB31857, PRJEB37122). The origin of these data is conveniently cited. However it appears that they are under a data sharing statement (see e.g., https://www.ncbi.nlm.nih.gov/bioproject/533273). I think that the authors must give evidence that they have contacted the consortium having produced the data and signed up the data sharing agreement.
[1.9] We are really sorry that we unintentionally missed the data sharing statement of the 160 goat genomes when retrieved them from NCBI (PRJEB31857, PRJEB37122). After we know this situation, we contacted the VarGoats Consortium who have produced the data, and negotiated the use of these goat genomic data in our study. Unfortunately, the consortium didn’t permit us to use the data because they are conducting a similar study on goat SVs. Under this circumstance, we have to remove these 160 goat genomes from the present study. We also removed an additional goat genome with unknown information about its breed name. Thus, we have 281 modern goat genomes and 84 ancient goat genomes for SV analyses in the revised version.

Although the amount of goat genomic data have been reduced, we found that in general the main results were not affected because the number of identified SVs and SV-annotated genes from 281 goat samples was comparable to those detected from 442 goat samples in the previous version. Also, the important candidate genes (e.g., BMPR2 and BMPR1B highlighted in this study) were still identified in relevant selection tests based on the updated goat samples. Additionally, we used separate goat samples for the GWAS analysis of litter size trait, so this part of results should not be affected.

Furthermore, the new dataset and new analyses used in this round of revision indeed improved the quality of the manuscript a lot. For example, we included new ATAC-Seq data and corresponding whole-genome sequencing data of sheep for peak-SV analyses. We used more strict methods for selection tests (e.g., DISV and PBS) and introgression analyses (e.g., specific method for SV-associated introgression based on allele frequency of each SV) to obtain more credible results. We also conducted a comprehensive evaluation for the assembly of Asiatic mouflon, and discussed potential issues associated with data usage (e.g., batch effect, small number of genomes for wild species).

Minor points
- in the Figures, the sheep and goat logos are taken from Alberto et al. 2018.I think the authors should get the authorization from Nature Communication and acknowledge the authors.
[1.10] We have replaced previous sheep and goat logos with the new logos drawn by ourselves throughout relevant figures. Please see Fig. 1-Fig 6.

- I was not able to find Tables S3 - S10 in the supplementary material
[1.11] We rechecked our submitted files in the online submission system of Genome Biology, and found that the file “Additional file 2.xlsx” in the supplementary material indeed contains Tables S3 - S10. Please find all the Supplementary Tables in the “Additional file 2” with “xlsx” format.

- Fig. 3G - please explicit the name of the x-axis on the figure.
[1.12] The x-axis of Fig. 3G represents the sheep individuals. We have added the name of the x-axis (i.e., Individual) on the Fig. 3G and also Fig. 3H for representing goat individuals.

- I do not understand Fig 5A. The legend should be much more explicit and/or the Figure redrawn in a clearer way
[1.13] Both Fig. 5A and its legend have been modified to make the figure more explicit to be understood. Please see the new figure 5 and lines 1773-1779.

- throughout the text : 'selective signature' → 'selection signature'
[1.14] Revised. Please see lines 74, 309, 325, 418, etc.

- Fig. 8 is cited in the text before Fig. 7
[1.15] We want to explain that Fig. 7 (line 428 in the previous version) is actually cited before Fig. 8 in the previous version. In the revised version, we have removed Fig. 8 to supplementary information.

- Fig. 6G (and S19) not so clear, please adjust the color-key
[1.16] Done. Please see new versions of Fig. 6G and Fig. S19.

- L 533 - 'these findings …' sentence not clear. Do you mean 'lower than' ?
[1.17] Yes, we mean “lower than”. This sentence should be “These findings indicated that SV-genes exhibited relatively low expressions than non-SV-genes”. However, because we don’t have the same samples for identifying SVs and measuring gene expression, and the second comment in your “Main concerns” said that “I don't see the interest of these data (Results section 'impacts of Svs on the gene expression…' and related discussion)”, we removed all relevant contents about the impact of SVs on gene expression from the paper. Therefore, this sentence has been deleted in the revised version.

- L 580 … I don't see the difference between what is said in this sentence and and in L 577.
[1.18] The sentence in L580-582 has been removed (i.e., “Additionally, differences in genomic features between domestic sheep and Asiatic mouflon will help to identify the genomic signatures associated with domestication and reveal the genetic basis underpinning phenotypic changes.”).

- L 608 in some of the references cited at least some genes have been identified on the basis of SV analyses.
[1.19] We rechecked the references cited in this sentence, and found that reference 10 was a study based on structural variations and reported BMPR1B among the listed genes. Because we intended to present previous reported genes and novel genes rather than whether previous reported genes were identified from SNPs or SVs, we retained BMPR1B and modified the sentence as “...identified based on SNP or SV analyses [11–13, 61]”. Please see line 677.

- Paragraph beginning L 669 - As it is about data quality on which the whole study rely, I would shift this paragraph at the beginning of the discussion.
[1.20]We shifted the paragraph L669-688 to the second paragraph of the discussion according to your suggestion. Please see lines 639-658.

- L 923-24. Why was this study done only on goats ? This does not prejudge what may happen on sheep. You should repeat the same analysis on sheep.
[1.21] The population structure analyses (i.e., PCA, phylogenetic tree, genetic structure and linkage disequilibrium) of sheep based on the whole-genome SNPs from the same sheep samples in this paper have been conducted in a previous study (Lv et al. 2022, reference 11 in this paper), so we didn’t performed these analyses on sheep. To make it clear, we modified relevant contents in the Methods section in the revised version. Please see lines 1025-1026.

- L950-51: please give details on how VCFtools estimates the global Fst
[1.22] The details have been added to the relevant part of the Methods section. Please see lines 1057-1061.

- L 955-56 : Fst are estimated between populations with contrasting phenotypes, but we can see in Table S27 that some generalist breeds are referenced in several groups (i.e.traits). How was it handled?
[1.23] Good comment. As a sheep breed usually has multiple traits, we used some generalist breeds (e.g., Tan sheep, Altay sheep and Booted goat) which don’t have excellent phenotypes in particular agronomic traits as the reference population in several groups (i.e., traits) in the previous version. We considered that the generalist breeds and the targeted breeds would have the largest difference in phenotype of tested trait among all traits, thus most signals detected in the selection tests could be associated with the tested trait. Also, we refined the results and reported only the genes and functional enrichment with known evidences for its association with the tested traits. This could at least partly solved the issue of generalist breeds.

To avoid potential bias resulting from the use of generalist breeds, we tried our best to eliminate the generalist breeds which are repeatedly referenced, and re-conducted the selection tests for the specific traits using a new method (i.e., PBS method) in the revised version. Please see Additional file 2: Table S28.

- L 1024-25: not clear if there is polymorphism in the populations and how it is handled.
[1.24] We don’t know if there is polymorphism in the populations because the genome sequencing data of the populations are not available. Actually, we wanted to use publicly available RNA-Seq data of sheep and goats to test if the expression of BMPR1B and BMPR2 in reproduction associated tissues (e.g., ovary, corpus luteum, endometrium and cervix) would be higher than that in the other tissues, thus providing evidences for their roles in reproduction. We can’t compare the expression of BMPR1B and BMPR2 across different tissues and among different populations with or without the deletions due to the lack of corresponding samples between RNA-Seq data and SV data. Therefore, we removed the wrong description here (i.e., “and among different populations with or without the deletions”). Please see lines 1136-1137.

- L 1082 'mutual SV'. I don't understand what you are referring to.
[1.25] “mutual SV” is referring to “common SV”. The word has been revised. Please see line 1188.

Reviewer #2:
Yang et al. reported a very comprehensive investigation of convergent evolution between two closely related domestic animal species, sheep and goat, based on structural variants (SVs) at the whole-genome level. This is a very interesting study from several aspects: (1) as I know, this is the one of a few, or perhaps the first, SV-based convergent genomic evolution between phylogenetically related mammals with such as a large sample size; (2) samples of the two species are from a worldwide geographic origins and with similar artificial selective pressures for similar phenotypes such as milk, meat and fiber; (3) populations of the two domestic species might have experienced wild introgressions, which might lead to similar consequences like local adaptation; (4) most interestingly, they characterized one SV in one functional gene which explained around 10% of the phenotypic variance, and (5) Last, the analyses in the work are very thorough and detailed. However, I have several major comments and minor comments for improving this manuscript. In summary, I suggest that this manuscript could be accepted for publication after resolving the below comments.
Thank you for your positive comments. We have addressed all your concerns. Please see the point-to-point responses below.

1．Convergent evolution between sheep and goat has been studied previously based on genome-wide SNPs [Nature communications 9: 813 (2018)]. I think this study is a significant advance in SV-based convergent evolution, but I wonder what were the differences in the results and conclusions between this study and the earlier investigation? The similarity and differences in results should be discussed in more details. Also, I would like to see the overlapping signals detected between the two studies and to see if there is link between the SNPs detected in the previous study and the SVs detected here?
[2.1] Good comment! By comparing the present study and Alberto et al. (2018) [i.e., Nature communications 9: 813 (2018)], we found the main differences as follows: (1) this study included all seven wild species in genus Ovis and most (i.e., 6 of 8) wild species of Capra as well as worldwide domestic sheep and goat populations with large sample size, while previous study included only one wild species (i.e., wild ancestor) and several domestic breeds for both Ovis and Capra with relatively small sample size; (2) previous study investigated convergent selection signatures for sheep and goat domestication and assessed the patterns of selection (e.g., positive selection, relaxation of selection), while this study performed genomic selection tests for convergent signatures for domestication, improvement and particular traits and focused on the signatures of positive selection for sheep and goats. This study also investigated potential convergent signatures of genetic introgression and environmental association in domestic sheep and goats; (3) this study identified much more common candidate genes (this study, 31 genes; previous study, 9 genes) under selection during the domestication of sheep and goats, probably due to much larger sample size included in this study and the greater effect of SVs on phenotypic traits than that of SNPs. This study provided much more genomic targets potentially useful for future genetic improvements of domestic sheep and goats.

We didn’t find any overlapped genes of the common candidate genes associated with the domestication of sheep and goats between the two studies, so we can’t check whether there is link between the SNPs detected in the previous study and the SVs detected here within the overlapped genes. We have added a brief discussion for the differences of the results between this study and previous study in the revised version. Please see lines 701-706.

2．A chromosome-level genome assembly of Asiatic mouflon was reported. This is a useful resource for the future studies on sheep domestication. I have two points here: (1) although table 1 has summarized the main parameters for the assembly, but I would I like to see a comparison of the parameters between this assembly and the published assemblies of sheep and other related species. Also, a short discussion on the comparison would make sense, at least for their utilization in domestication analyses etc. (2) minor discussion on the future T2T assembly in charactering SVs would be necessary, whatever for Asiatic mouflon and domestic sheep.
[2.2] Following your suggestion, we collected all the publicly available assemblies of domestic sheep (chromosome level) and related wild sheep species (chromosome and scaffold level), and compared the parameters (e.g., Total length, Contig N50, and Scaffold N50) between the assembly of Asiatic mouflon and these published assemblies (please see Additional file 2: Table S2). The results showed that Asiatic mouflon has the longest scaffold N50 (103.69 Mb) and the second longest contig N50 (42.16 Mb) among the three chromosome-level genome assemblies of wild sheep species (argali, bighorn sheep, and Asiatic mouflon), and the parameters are well comparable to the latest reference genome of domestic sheep (ARS-UI_Ramb_v3.0, with contig N50 of 43.18 Mb and scaffold N50 of 101.27 Mb) in NCBI. This indicated the good quality and high credibility of our Asiatic mouflon genome assembly. Because Asiatic mouflon is the wild ancestor of domestic sheep, the first chromosome-level assembly reported in this study will be of great importance in exploring the genetic mechanisms of sheep domestication. Also, the assembly of Asiatic mouflon can be integrated with assemblies of other wild sheep and domestic sheep (Additional file 2: Table S2) to conduct the pangenome analysis of the ovine species and exploit the the molecular mechanisms underlying genome evolution of the Ovis genus. We added these new contents to the Results and Discussion section in the revised version. Please see lines 89-99 and 619-620 and Table S2.

In addition, we also briefly discussed the advantage of the T2T assembly technology and the implications of future T2T assembly of domestic sheep and Asiatic mouflon on SV detection and domestication analyses. Please see lines 621-628.

3．Some caveats in the data should be pointed out as well. There are only one or very small sample sizes for some wild sheep or goat species, which can be due to the limitation to access a large sample size of some wild animals, in particular some wild protected animals. The bias and effect on SV identification and characterization due to small sample size should be discussed. In addition, the difference in sequencing depth may also lead to biases in SV characterization.
[2.3] We added some discussion about the potential effect on SV identification and characterization due to the small sample size (e.g., < 5 samples) and low sequencing depth (e.g., < 10×) of some wild sheep (e.g., urial, argali, European mouflon, and snow sheep with small sample size) and wild goat species (e.g., Iberian ibex, Nubian ibex, and markhor with small sample size; one Alpine ibex and five Siberian ibex individuals with low sequencing depth), and provided relevant reference papers [1-3] to support the discussion.

The difference in sequencing depth should not lead to obvious bias in SV characterization in this study, because the sequencing depth of most (787/813) modern sheep and goat samples is higher than 15×, which has been indicated as an adequate depth for accurate detection of SVs from short-read sequencing data [4]. Please see lines 751-769.

Reference:
1.Chen X, Schulz-Trieglaff O, Shaw R, Barnes B, Schlesinger F, Källberg M, et al. Manta: rapid detection of structural variants and indels for germline and cancer sequencing applications. Bioinformatics. 2016;32:1220–2.
2.Rausch T, Zichner T, Schlattl A, Stütz AM, Benes V, Korbel JO. DELLY: structural variant discovery by integrated paired-end and split-read analysis. Bioinformatics. 2012;28:i333–9.
3.Layer RM, Chiang C, Quinlan AR, Hall IM. LUMPY: a probabilistic framework for structural variant discovery. Genome Biol. 2014;15:R84.
4.Gong T, Hayes VM, Chan EKF. Detection of somatic structural variants from short-read next-generation sequencing data. Brief Bioinform. 2021;22(3):bbaa056.

4． As to the SV-based introgression analyses, it would be very necessary to justify the correct utilization of the method in the analyses. For example, how this method has been used in the similar analyses of introgression between other species, and why this method is suitable for the detection of introgression between wild and domestic sheep/goat species.
[2.4] Based on your comment and the comment from reviewer 1, we used a new method based on the allele frequency of each SV [1, 2] to perform introgression analyses for domestic sheep/goat populations and their congeneric wild relatives. This method can strictly exclude false positive signals of introgression resulting from common ancestor, and was also used to detect genetic introgression from wild cattle to domestic cattle populations [2] and from other Bos species to indicine cattle [1] based on SV variants. Therefore, we think the new method is suitable for the introgression analyses between wild and domestic sheep/goat species in this study. Please see the description of the new method in lines 1156-1169.

Reference:
1.Dai X, Bian P, Hu D, Luo F, Huang Y, Jiao S, et al. A Chinese indicine pangenome reveals a wealth of novel structural variants introgressed from other Bos species. Genome Res. 2023;33(8):1284-98.
2.Xia X, Zhang F, Li S, Luo X, Peng L, Dong Z, et al. Structural variation and introgression from wild populations in East Asian cattle genomes confer adaptation to local environment. Genome Biol. 2023;24(1):211.

Minor comments:
1.Too many references. I suggest to omit some unnecessary references.
[2.5] Following your comment, we omitted some unnecessary references, but due to the extensive new analyses and results in this round of revision, we also added some necessary references. Although the total number of references is quite high, the references in the main parts (e.g., exclude Methods section) of this manuscript is around 80.

2.The whole manuscript reads well, but some sentences could be improved by a native English speaker.
[2.6] The manuscript has been proofread and modified by a native English speaker.

3. Too many figures. Some parts in some figures could be moved to supplementary information.
[2.7] Based on the changed contents in this round of revision, we moved one main figure about the introgression results (previous Fig. 8) to supplementary information (Additional File 1: Fig. S26). We also deleted the expression results of SV-genes and nonSV-genes in Fig. 7A and B, and revised other main figures (Figs. 1-6).


Reviewer #3: Review of "Structural variant landscapes reveal convergent signature of evolution in sheep and goats" by Yang et al.
This study reports the outcomes of an in-depth analysis of structural variants across multiple breeds/origins for both sheep and goats. Additionally, long-read sequencing was conducted for the generation of an Asiatic mouflon de novo assembly. SVs were interrogated for characterization relating to genetic diversity, chromosomal distribution, gene links, QTLs, transposable elements, and impact on gene expression and regulatory elements. This allowed for the linkage with known phenotypes and evolutionary convergence. This data can be a basis for further hypothesis testing and comparison to other livestock species. Overall, the strengths of this manuscript include the breadth of sample inclusion and utilization of multiple SV callers to mitigate software biases. However, there are a few weaknesses: while the selection analysis was a large portion of the manuscript, it feels weak. A more sophisticated methodology could be undertaken as it relates to the FST outliers' analysis, and there was no control for demographic history.
Thank you for your comments. We have tried our best to address all your concerns, especially the method for selection tests. In the revised version, we used a combination of FST method (e.g., FST and associated P values) and DISV method to identify more credible selection signatures [1, 2], and employed the PBS method to control the potential influence of demographic history on selection tests [3].

PBS analysis is an estimate of the allele frequency change at a given locus in the history of a population since its divergence from another population. The concept behind this analysis is that if we consider two related populations and an outlier population (e.g., ancestral population), the allele frequency changes at any locus in these two populations should be equidistant (or have similar branch length) from the outlier. Therefore, loci which show high differentiation in allele frequency in only one of the related populations could be reflected by high population branch length (PBS score), and may be potential candidates for selective sweeps [4]. The inclusion of an outlier population (e.g., ancestral population in this study) in the PBS analysis enabled us to control the impact of demographic history on genome-wide selection test. Please see the point-to-point responses below.

Reference:
1.Li R, Gong M, Zhang X, Wang F, Liu Z, Zhang L, et al. A sheep pangenome reveals the spectrum of structural variations and their effects on tail phenotypes. Genome Res. 2023;33:463-77.
2.Bertolotti AC, Layer RM, Gundappa MK, Gallagher MD, Pehlivanoglu E, Nome T, et al. The structural variation landscape in 492 Atlantic salmon genomes. Nat Commun. 2020;11:5176.
3.Hämälä T, Savolainen O. Genomic Patterns of Local Adaptation under Gene Flow in Arabidopsis lyrata, Mol Biol Evol, 2019;36(11):2557–71.
4.Choudhury A, Hazelhurst S, Meintjes A, Achinike-Oduaran O, Aron S, Gamieldien J, et al. Population-specific common SNPs reflect demographic histories and highlight regions of genomic plasticity with functional relevance. BMC Genomics. 2014;15(1):437.

General Comments:
- Please review manuscript, figure legends, and Additional File for grammatical and spelling errors.
[3.1] We have carefully reviewed and revised the grammatical and spelling errors throughout the manuscript, figure legends, and Additional File. The manuscript has been also proofread and modified by a native English speaker.

- Ensure format of abbreviations are the same throughout (example: non-SV-genes vs. nonSV-genes).
[3.2] All “non-SV-genes” have been replaced with “nonSV-genes” throughout the manuscript.

- For some of the figures, it is advised to change the color palette to colorblind friendly, e.g., substituting green for a different color.
[3.3] The color green was substituted for another color based on the colors used in specific figures.

- An increase in background on goat and sheep divergence/comparison would be helpful.
[3.4] We added a brief description of the background regarding the divergence of the genus Ovis and Capra and the parallel domestication of sheep and goats in the Background section. Please see lines 56-60.

- The results section addressing the de novo assembly could be expanded, particularly as it relates to the methods.
[3.5] The previously missed results of de novo assembly that relates to the methods, such as genome survey, assembling process, kmer completeness, and annotation of repeats and genes, have been added to the relevant part of the Results section. Please see lines 101-114, 119-123 and 128-140.

- Why were hotspots intersected with QTLs? Was there an expectation that multiple variant loci would be selected for production traits?
[3.6] SV hotspots could be intersected with QTLs because structural variations are known to have large functional impacts on phenotypic variations of livestock. For the second question, we observed many cases in our results that multiple SV loci were selected for QTLs of particular production traits (Additional file 2: Table S11).

- Were the p-values from the GO output corrected for multiple testing?
[3.7] Yes, the p-values in the GO analyses were corrected for multiple testing. To this end, false discovery rate (FDR) was computed with the Benjamini-Hochberg method. We added this missed information to the relevant part of the Methods section, and revised the presentation of all relevant p-values to FDR values throughout the manuscript. Please see lines 1071-1073.

- For the de novo genome assembly statistics. Per the Earth Biogenome project's standards, it is recommended to include a kmer completeness analysis, aiming for greater than 90%.
[3.8] We conducted kmer analysis and obtained a kmer completeness of 93.15% for the Amuf_v1 assembly. Please see lines 120-121.

- For the genetic structure analysis. Please provide background information on why the values K=5 and K=6 were selected for plotting. Could you provide cross-validation values to justify.
[3.9] We estimated cross-validation error values for each K, and found that the values have little difference when K is from 4 to 10 for both sheep and goats. Thus, we chose the most probable K based on the known population structure of sheep and goats [reference 11 and 61 in the main text], which could have logically biological meaning. Please see Fig. 1D and E and Fig. S9.

- The deletion analysis feels a bit speculative. While an intronic deletion may be an effect, more data is needed. Is there specific binding sites that show evidence of conservation or other signals of enhancers?
[3.10] For the intronic deletion (i.e., DEL00034481) in BMPR1B gene in sheep, we found a peak at chr6:34,039,320–34,039,587 located within 5 Kb of its downstream region. Also, we identified a peak at chr2:218,821,915–218,822,146 located within 1 Kb of the upstream region of the intronic deletion (i.e., SV_w_15555) in BMPR2 gene in sheep. The findings indicated that the intronic deletions in BMPR1B and BMPR2 are likely to locate in the enhancer regions, thus could have functional implications for the prolificacy traits of sheep. The new results have been added to the relevant part of the Results section. Please see lines 472-474 and 528-530.

- Is the expression data is from different samples? If so, it is unclear how it is then integrated with the SV data
[3.11] Yes, the expression data is from different samples. Based on the comment from reviewer 1, we removed relevant contents about the impact of SVs on gene expression from the paper.

Specific Comments:
Line 63 : Recommend reordering words, placing "de novo" after "assembled a": "Here. We assembled a de novo high-quality…" .
[3.12] Revised as suggested. Please see line 68.

Line 65: Please be more explicit in what ">15x" reference.
[3.13] To make it more clear, “> 15×” has been revised to “sequencing depth > 15×”. Please see line 70.

Line 67-68: It is indeed a large and comprehensive dataset/analysis; however, it is recommended to temper the verbiage to being less absolute. E.g., "large dataset" and "one of the most comprehensive".
[3.14] The phrases “large dataset” and “one of the most comprehensive” have been revised to “dataset” and “a comprehensive”, respectively. Please see lines 72-73.

Line 69: The word "legacy" seems out of place. Suggest a different word choice or reworking sentence to remove.
[3.15] The word “legacy” has been revised to “selection signatures”. Please see line 74.

Line 102: What is the "PAV" distribution. Please spell out PAV before abbreviation.
[3.16] The full name of PAV, i.e., presence and absence variation, has been added. Please see line 150.

Lines 105-109: Any statistics available to quantify these statements?
[3.17] We added the statistics of collinearity (e.g., percentage) between these genomes and relevant reference paper [1] to support the statements. Please see lines 152 and 154.

Reference:
1.Liu D, Hunt M, Tsai IJ. Inferring synteny between genome assemblies: a systematic evaluation. BMC Bioinformatics. 2018;19(1):26.

Line 113: Can you break out the number of samples to quantify the number of wild and domestic?
[3.18] Revised as suggested.

Line 115: Please check sheep values. Per Supplemental Table S1 and S3, mean depth for sheep is 18.32 (15.02-30.11). Coverage values match.
[3.19] Revised.

Lines 116-117: Please goat values. Per Supplemental Table S1 and S3, mean depth values is 20.04 (5.97-41.06).
[3.20] Revised. Because the goat samples have been changed in the revised version, the mean depth value is 21.45× (5.97× – 41.06×).

Line 114 and 117: It is stated that a minimum depth of >15x for the study. However, there are a number of goat samples below that value.
[3.21] Here all the sheep samples and 255 out of 281 (i.e., 90.75%) goat samples have a sequencing depth > 15×. The sentence has been modified accordingly. Please see lines 163-164.

Line 149: To ease reading comprehension, please spell out deletion and duplication before abbreviation.
[3.22] Revised as suggested. Please see line 200 and 201.

Line 184: Suggest changing "…the numbers in the introns…" to "intronic".
[3.23] Revised.

Line 162: What is a "hotspot"? A clearer definition would be helpful.
[3.24] The definition of “SV hotspots” (i.e., the regions with the top 10% of SV breakpoints) has been added. Please see line 215.

Line 188: Were most of these genes with SVs in the intron? What was the proportion of intron/exon? How many genes had an SV overlapping with an exon?
[3.25] Yes, most of the genes had SVs in the intron for both sheep (7,776 genes, 65.65%) and goats (8,685 genes, 67.28%). The proportion of genes having SVs in the intron and exon is 5.17 for sheep and 5.03 for goats. There were 1,505 (12.71%) and 1,725 (13.36%) genes had SVs in the exon for sheep and goats, respectively. Please see lines 243-248.

Line 218: Why would the LD be high in the wild species? This is the opposite of what I would have expected.
[3.26] High LD levels observed in the wild sheep/goat species based on both SVs and SNPs may mainly result from recent decline of population sizes in the wild sheep/goat species and large differences in the sample sizes of wild and domestic sheep/goats used for LD analyses. We found one recent study of grape which reported similar higher level of LD in the wild species than that in the cultivated populations based on both SVs and SNPs [1].

Reference:
1.Zhou Y, Minio A, Massonnet M, Solares E, Lv Y, Beridze T, et al. The population genetics of structural variants in grapevine domestication. Nat Plants. 2019;5(9):965-79.

Line 219: Was the pi based on SVs or SNPs? Accordingly, what does the LD pattern look like with SNPs?
[3.27] The pi value was based on SVs, and we have modified the sentence to make it clear. Following your comment, we used SNP data to estimate genome-wide linkage disequilibrium values, and found similar LD pattern with that based on SV data for wild, native and improved sheep/goats. We have drawn a new supplementary figure and added the content to the relevant part of the Results section. Please see Fig. Sxx and lines 276-278.

Line 335: here is limited overlap between SNP and SV FST outlier. This is a sign of power, genotyping error, etc. Would expect a true selected signal to have a long haplotypic signal that is also well tagged by SNPs.
[3.28] The limited number of overlapped genes observed between SNP and SV FST outlier may be due to the power of method, genotyping error, and different characteristics of genetic markers (e.g., many selective signals could be solely detected by one type of marker). In the revised version, we used a more complicated method (i.e., PBS method), which can control the influence of demographic history, to identify selection signatures for prolificacy traits based on both SV and SNP markers. We think the identified selection signatures and candidate genes should be more credible than those in the previous version. The updated results showed that there were 580 SNP-based and 403 SV-based candidate genes for sheep prolificacy traits and 750 SNP-based and 282 SV-based candidate genes for goat prolificacy traits, among which 38 and 31 candidate genes were overlapped between the two types of markers for sheep and goats, respectively. Please see lines 380-386.

We agree with you that a haplotype-based method based on SNP data can help to find a true selected signal. However, we didn’t find applicable program that can be successfully operated for our SNP data in this round of revision.

Line 404: Space needed: "DEL00034481deletion" to "DEL00034481 deletion".
[3.29] Revised.

Line 407: IGV visualization is not true "validation", but nonetheless makes a clear representation of the SV.
[3.30] We replaced the word “validated” with “clearly visualized” in this sentence.

Line 533-534: Sentence starting with "These findings…". Should the sentence end with "… low expression than non-SV-genes"?
[3.31] Yes, the sentence should end with “… low expression than non-SV-genes”. However, following the comment of reviewer 1, we removed relevant contents about the impact of SVs on gene expression from the paper. Therefore, this sentence has been deleted in the revised version.

Line 539: What is the definition of Peak vs non-peak SV? This seems like useful data that could be used to annotate potential enhancer regions.
[3.32] The definitions of Peak-SVs and nonPeak-SVs have been already presented in the Methods section, that is SVs with at least 50% of their length overlapping with the merged peaks as “Peak-SVs” and the remaining SVs as “nonPeak-SVs”. To facilitate the understanding of Peak-SVs and nonPeak-SVs here, we added the definition of Peak-SVs (i.e., SVs with at least 50% of length overlapping with peaks) in this sentence. Please see lines 572-573.

As previous studies indicated that peak regions revealed from ATAC-Seq data can represent functional elements in the genome, peaks have been used to annotate promoter (or transcription start site) and putative enhancer [1, 2]. On the other hand, structural variations are known to have functional impacts on evolution, adaptation, and phenotypic traits. In this study, taking advantage of the same sheep samples of ATAC-Seq data and whole-genome sequencing data, we investigated potential enhancer regions based on a combination of peaks and SVs. We identified a total of 706,742 peaks in sheep genome. After excluding 60,621 peaks annotated as promoters, there were 646,121 peaks as potential enhancers, in which 75,449 were overlapped with SVs (i.e., at least 50% of SV length) and could be annotated with Peak-SVs. We considered the 75,449 candidate enhancers as the most probable enhancer regions in sheep genome. These new contents have been added to the relevant part of the Results section. Please see lines 576-586.

Reference:
1.Schwope R, Magris G, Miculan M, Paparelli E, Celii M, Tocci A, et al. Open chromatin in grapevine marks candidate CREs and with other chromatin features correlates with gene expression. Plant J. 2021;107(6):1631-47.
2.Cao X, Cheng J, Huang Y, Lan X, Lei C, Chen H. Comparative Enhancer Map of Cattle Muscle Genome Annotated by ATAC-Seq. Front Vet Sci. 2021;8:782409.

Line 584: Suggest reworking sentence starting with "The amount of…". E.g., "The high number of included genomes in our study (532 sheep and 442 goats) could ensure…".
[3.33] Revised as suggested.

Line 593: Include oxford comma following "genetic structure".
[3.34] Revised.

Line 634: Include oxford comma following "gene expression".
[3.35] Revised.

Line 650: Include oxford comma following "apple".
[3.36] Revised.

Line 654: Strike "good".
[3.37] The whole sentence was deleted because all relevant contents about the impact of SVs on gene expression have been removed from the paper based on the comment of reviewer 1.

Line 658: Change analysis to analyses.
[3.38] Revised.

Line 662: Include oxford comma following "conformation".
[3.39] Revised.

Line 673: Include oxford comma following "Delly".
[3.40] Revised.

Line 686: Include oxford comma following "graph".
[3.41] Revised.

Line 691: Strike "on".
[3.42] The word “on” has been deleted.

Lines 729-730: Please check values as they are different than those reported in Lines 115-118.
[3.43] We checked the values in the two places and relevant supplementary tables, and found that the value in lines 115-118 are incorrect. We revised the wrong values accordingly.

Line 738: Include oxford comma following "small intestine".
[3.44] Revised.

Line 815: It is recommended that the newest version of BUSCO (v5.4.7) and database (odb10) be utilized.
[3.45] Following your comment, we re-performed the BUSCO analysis using the recommended program version and database. The updated methods and results have been added to the relevant part of the Methods and Results section, respectively. Please see lines 856-858.

Line 934: Include oxford comma following "early development".
[3.46] Revised.

Line 1026: Hisat2 to HISAT2. DeSeq2 to DESeq2.
[3.47] Revised.

Line 1051: Hisat2 to HISAT2. Please include HISAT2 version.
[3.48] The whole paragraph was deleted because all relevant contents about the impact of SVs on gene expression have been removed from the paper based on the comment of reviewer 1. The version of HISAT2 has been added to the relevant part of the RNA-Seq data analysis in the previous paragraph (line 1145).

Line 1052: Please include versions of featureCounts and DESeq2. DeSeq2 to DESeq2.
[3.49] The whole paragraph was deleted because all relevant contents about the impact of SVs on gene expression have been removed from the paper based on the comment of reviewer 1. The versions of featureCounts and DESeq2 have been added to the relevant part of the RNA-Seq data analysis in the previous paragraph (line 1145).

Tables:
Table 2: The number of translocations are unlikely to be real. Are the duplicated/mapability issues in the assemblies used?
[3.50] We have explored recent papers, and found that the information about translocation was less reported among the SV types. Studies based on long-read sequencing usually reported a small number (e.g., dozens to hundreds) of translocations [e.g., 1]. Nevertheless, one recent study reported a quite large number (i.e., 61,682) of translocations in peach genomes based on short-read sequencing data [2].

We checked the issue of duplicates and confirmed that this does not exist in our data. We can’t check and modify mapability because it is an intrinsic characteristic of the program. Under current situation, we would like to keep the relevant results of translocation in this round of revision.

Reference:
1.Chen J, Liu Y, Liu M, Guo W, Wang Y, He Q, et al. Pangenome analysis reveals genomic variations associated with domestication traits in broomcorn millet. Nat Genet. 2023;55(12):2243-54.
2.Guo J, Cao K, Deng C, Li Y, Zhu G, Fang W, et al. An integrated peach genome structural variation map uncovers genes associated with fruit traits. Genome Biol. 2020;21(1):258.

Table 3: Have same justification (either right or centered) for both rows.
[3.51] Both rows have been center justified. Please see Table 3.

Figures:
Figure 1: Panels B and C: Please include accounted variance for each principal component.
[3.52] Accounted variance for each principal component has been added to Figs. 1B and C.

Figure 1: Panel D and E: As mentioned above, please provide justification for K selection and include cross-validation values for K values. Figure legend states K 2-5 and 2-6, when only K 2,5 and 2,6 are plotted.
[3.53] As we responded above, we estimated cross-validation error values for each K, and found that the values have little difference when K is from 4 to 10 for both sheep and goats. We then chose the most probable K based on the known population structure of sheep and goats [11, 54]. The cross-validation error values for corresponding K have been included in Fig. S9, and the figure legend also has been modified based on your comment. Please see Fig. 1D and E, Fig. S9 and line 1736.

Figure 2: Panel C: Why is the limit 1kb?
[3.54] Because more than 80% of the identified SVs are shorter than 1 kb (Table S7), we set the range of x axis in Fig. 2C as 50 - 1,000 bp. In the revised version, we added a supplementary figure (Fig. S3) to show the size distribution (1 kb - 1 Mb) of SVs by variant types in sheep and goats.

Figure 2: Panel E and F: Figures are difficult to discern differences and trends across the groups. Suggest a different format of plot to better delineate the groupings.
[3.55] To make the information in Fig. 2E and F easy to understand, we redrew the figure with an explicit pattern. The new Fig. 2E and F showed the differences in SV numbers among wild, native and improved groups for each SV type. Please see Fig. 2.

Figure 4: Panel H: Value for C. sibirica-C.nubiana is not centered in box.
[3.56] Revised. Please see Fig. 4H.

Figure Legends:
Line 1587: Additional fle to Additional File.
[3.57] Revised.

Line 1632: Additional fle to Additional File.
[3.58] Revised.

Line 1637: Additional fle to Additional File.
[3.59] Revised.

Line 1670: Additional fle to Additional File.
[3.60] Revised.

Line 1697: Additional fle to Additional File.
[3.61] Revised.

**Second round of review**

**Reviewer 1**

The authors made an extensive revision of their work, based on new analyses and improved methods. The Ms, which was already very good, has been greatly improved.
I am satisfied with all answers to my previous comments.
I only have suggestions for rephrasing 2 paragraphs and then a few minor points.

As there is no clear consensus on what differentiate parallelism from convergence, I do agree with authors who have now specified their understanding of both terms at both the phenotype and genotype levels. However, I found that their definition could be more explicit and would propose to rephrase L 413 -417 as follows “There is non consensus on the precise distinction between convergent and parallel evolution [36] and, considering that the two genera correspond to different lineages, we regarded the similarities between sheep and goats as resulting from convergent evolution (e.g., convergent selection). At the molecular level, following Woodhouse and Hufford [37] we considered that molecular parallelism occurred when convergent traits are caused by modification of the same genes."

L31-34. Given the previous definitions provided by the authors (the traits are convergent but there is parallelism at the molecular level when orthologous genes are involved), I would rephrase as follows:
"We detected signatures of selection involving the parallel evolution of orthologous SV-associated genes during domestication, local environmental adaptation and improvement. In particular, we found that fecundity traits experienced convergent selection targeting the gene BMPR1B, with the DEL00067921 deletion explaining ~10.4% of the phenotypic variation observed in goats. "


Minor points

L 63-66 : It is a general case that pioneering studies are based on less sampling ; however, each of the 2 studies cited analysed > 100 WGS (with > 20 wilds) with coverages > 12x. So I don’t think you can say ‘few samples’ and ‘low coverage’. I would remove the second part of the sentence ‘based only on …”.

L 418: genes that were parallel selected → remove ‘parallel’

L 523: ’to have experienced convergent selection’ → ’to have been under convergent selection’

L 715: the difference with Ref 12 in the number of common candidates detected among sheep and goats can also be due to differences in the methods used (e.g., stringency of the detection).

Results and Table 3: As the sampling and the SV discovery methods was unchanged for Ovis, I was not expecting the number of whole SV to change for sheep as it can be seen in Table 3. I probably missed something … could you explain that please ?

Fig 2C. remove the sheep and goats icons (both graphs present Ovis + Capra data)

Fig 2F. Error in x-axis legend (‘sheep’ instead of ‘goats’)

Fig 5E. Maybe ‘expected/observed’ would be more explicit than ‘control/input’ in the legend

Additional file 1- please check typos (significant l 67, candidate l 109 , etc…)

Fig S1A. The legend specifying the scale of the colour code (blue to dark red) is lacking

Table S7. Why the number of inversions of 50-100 bp is higher with less goats genomes analysed (80 vs 70 in Table S6 of the previous version)

**Reviewer 2**

The revised manuscript has resolved my previous comments and now improved very much. Although the revised manuscript removed the VarGoats genome data, the main conclusions were not affected. Moreover, following the reviewers’ comments, the authors supplemented some new analyses. So, I recommend to accept the revised manuscript for publication.

**Reviewer 3**

I thank the authors for diligently addressing all questions/comments to my satisfaction.

**Authors Response**

**Point-by-point responses to the reviewers’ comments:**

Reviewer #1: The authors made an extensive revision of their work, based on new analyses and improved methods. The Ms, which was already very good, has been greatly improved.

I am satisfied with all answers to my previous comments.

I only have suggestions for rephrasing 2 paragraphs and then a few minor points.

Thank you for your positive comments! We have addressed all your concerns. Please see the point-to-point responses detailed below.

As there is no clear consensus on what differentiate parallelism from convergence, I do agree with authors who have now specified their understanding of both terms at both the phenotype and genotype levels. However, I found that their definition could be more explicit and would propose to rephrase L 413 -417 as follows “There is non consensus on the precise distinction between convergent and parallel evolution [36] and, considering that the two genera correspond to different lineages, we regarded the similarities between sheep and goats as resulting from convergent evolution (e.g., convergent selection). At the molecular level, following Woodhouse and Hufford [37] we considered that molecular parallelism occurred when convergent traits are caused by modification of the same genes."

[1] Revised as suggested. Please see page 15.

L31-34. Given the previous definitions provided by the authors (the traits are convergent but there is parallelism at the molecular level when orthologous genes are involved), I would rephrase as follows:

"We detected signatures of selection involving the parallel evolution of orthologous SV-associated genes during domestication, local environmental adaptation and improvement. In particular, we found that fecundity traits experienced convergent selection targeting the gene BMPR1B, with the DEL00067921 deletion explaining ~10.4% of the phenotypic variation observed in goats. "

[2] Revised as suggested. Please see the ‘Results’ section in the Abstract.

Minor points

L 63-66 : It is a general case that pioneering studies are based on less sampling ; however, each of the 2 studies cited analysed > 100 WGS (with > 20 wilds) with coverages > 12x. So I don’t think you can say ‘few samples’ and ‘low coverage’. I would remove the second part of the sentence ‘based only on …”.

[3] Following your comment, the second part of the sentence “based only on ...” has been removed. Please see page 3.

L 418: genes that were parallel selected → remove ‘parallel’

[4] The word “parallel” has been removed. Please see page 15.

L 523: ’to have experienced convergent selection’ → ’to have been under convergent selection’

[5] Revised. Please see page 19.

L 715: the difference with Ref 12 in the number of common candidates detected among sheep and goats can also be due to differences in the methods used (e.g., stringency of the detection).

[6] We agree with your comment and have added this explanation to the sentence. Please see page 25.

Results and Table 3: As the sampling and the SV discovery methods was unchanged for Ovis, I was not expecting the number of whole SV to change for sheep as it can be seen in Table 3. I probably missed something … could you explain that please ?

[7] Good comment. The counted values of SV annotated locations for Ovis in Table 3 was slightly changed because of small counting errors in the first version of the manuscript. We have corrected these small errors in the second version, and then the number of whole SVs changed for sheep, although the sampling and the SV discovery method was unchanged.

Fig 2C. remove the sheep and goats icons (both graphs present Ovis + Capra data)

[8] Revised.

Fig 2F. Error in x-axis legend (‘sheep’ instead of ‘goats’)

[9] Revised.

Fig 5E. Maybe ‘expected/observed’ would be more explicit than ‘control/input’ in the legend

[10] Revised as suggested.

Additional file 1- please check typos (significant l 67, candidate l 109 , etc…)

[11] We carefully checked the previous Additional file 1 and corrected all the typos (i.e., assembly, transposable, significant, pathways and candidate in the titles of Tables S3, S19, S20, S21, S23, S39 and S40 in the section of the description of Additional file 2). It should be noted that the description of Additional files has been moved to the main text according to the editorial requirement.

Fig S1A. The legend specifying the scale of the colour code (blue to dark red) is lacking

[12] The legend for the colour code has been added to Fig. S1A.

Table S7. Why the number of inversions of 50-100 bp is higher with less goats genomes analysed (80 vs 70 in Table S6 of the previous version)

[13] Following your comment, we recalculated the numbers and found that the actual number of inversion of 50-100 bp is 84 for the dataset of 442 goats in the first version. We obtained 80 inversions of 50-100 bp with less number of 281 goats (Additional file 2: Table S7). The reason for the difference is that the script extracting the length categories of SVs had an error, which erroneously counted SVs among the length categories. We corrected this error in this updated revision. Please see the correct results for the 442 goat dataset below.

| **SV type** | **50-100bp** | **100bp-250bp** | **250bp-500bp** | **500bp-1kb** | **1-2kb** | **2-5kb** | **5-10kb** | **10-50kb** | **50-100kb** | **100-500kb** | **500kb-1Mb** | **Total** |
| --- | --- | --- | --- | --- | --- | --- | --- | --- | --- | --- | --- | --- |
| **Deletion** | 21,019 | 17,147 | 11,098 | 8,354 | 6,009 | 3,012 | 1,025 | 479 | 80 | 75 | 16 | 68,314 |
| **Duplication** | 807 | 3,291 | 1,151 | 694 | 559 | 194 | 94 | 114 | 24 | 13 | 1 | 6,942 |
| **Inversion** | 84 | 230 | 365 | 777 | 819 | 331 | 128 | 99 | 13 | 37 | 12 | 2,895 |
| **Insertion** | 1 | 0 | 0 | 0 | 0 | 0 | 0 | 0 | 0 | 0 | 0 | 1 |
| **Translocation** | - | - | - | - | - | - | - | - | - | - | - | - |
| **Total** | 21,911 | 20,668 | 12,614 | 9,825 | 7,387 | 3,537 | 1,247 | 692 | 117 | 125 | 29 | 78,152 |
